# MspI and Ile462Val Polymorphisms in CYP1A1 and Overall Cancer Risk: A Meta-Analysis

**DOI:** 10.1371/journal.pone.0085166

**Published:** 2013-12-31

**Authors:** Bin Wu, Kang Liu, Huaxing Huang, Jun Yuan, Wanqing Yuan, Shangqian Wang, Tingting Chen, Hu Zhao, Changjun Yin

**Affiliations:** 1 Department of Urology, The Affiliated Jiangyin Hospital of Southeast University Medical College, Wuxi, China; 2 Department of Urology, The First Affiliated Hospital of Nanjing Medical University, Nanjing, China; 3 The First Clinical Medical College, Nanjing Medical University, Nanjing, China; 4 Department of Orthopedics, The Affiliated Hospital of Medical College, Qingdao University, Qingdao, China; Baylor College of Medicine, United States of America

## Abstract

**Background:**

Cytochrome P450 1A1 (CYP1A1) is a member of the CYP1 family, which is a key enzyme in the metabolism of many endogenous substrates and exogenous carcinogens. To date, many studies have examined the association between CYP1A1 MspI and Ile462Val polymorphisms and cancer risk in various populations, but their results have been conflicting rather than consistent.

**Methods:**

To assess this relationship more precisely, a meta-analysis based on 198 publications was performed. Odds ratios (OR) and corresponding 95% confidence intervals (CIs) were used to assess the association. The statistical heterogeneity across studies was examined with a chi-square-based Q-test.

**Results:**

Overall, a significant elevated risk of cancer was associated with CYP1A1 MspI and Ile462Val polymorphisms for all genetic models studied. Further stratified analysis by cancer types revealed that the MspI polymorphism may increase the risk of lung cancer and cervical cancer whereas the Ile462Val polymorphism may contribute to a higher risk of lung cancer, leukemia, esophageal carcinoma, and prostate cancer. In the subgroup analysis by ethnicity, obvious associations were found in the Asian population for the MspI polymorphism while an increased risk of cancer was observed in Asians and Caucasians for the Ile462Val polymorphism.

**Conclusions:**

The results of this meta-analysis suggest that CYP1A1 MspI and Ile462Val polymorphisms contribute to increased cancer susceptibility among Asians. Additional comprehensive system analyses are required to validate this association and other related polymorphisms.

## Introduction

Cytochromes P450 (CYP450s) are heme-containing enzymes important to phase I-dependent metabolism of drugs and other xenobiotics [1]. Studies indicate that CYP enzymes participate in cellular functions such as the metabolism of eicosanoids, the biosynthesis of cholesterol and bile acids, synthesis and metabolism of steroids and vitamin D3, synthesis and degradation of biogenic amines, and the hydroxylation of retinoic acid and presumably other morphogens. However, the functions of several CYP enzymes remain unknown [2,3]. To date, many important single nucleotide polymorphisms (SNPs) have been identified in CYP genes, and such polymorphisms within these genes may play an important role in determining individual susceptibility to many cancers. Among CYPs involved in procarcinogen activation, Cytochrome P450 1A1 (CYP1A1) has been the most widely studied [4,5].

CYP1A1 is mainly expressed extrahepatically, especially in epithelial tissues, and it is critical for the metabolism of many endogenous substrates and exogenous carcinogens. Because of CYP450 1A1’s ability to catalyze the first step in the metabolism of polycyclic aromatic hydrocarbons (PAHs, also present in tobacco smoke), CYP1A1 may contribute to the formation of highly reactive intermediates [6,7] which can form DNA adducts, which, if unrepaired, can initiate or accelerate carcinogenesis [8]. 

Several polymorphisms of CYP1A1 have been described and current information can be found on the Human CYP allele nomenclature website (http://www.cypalleles.ki.se) [9]. Accumulating evidence suggests that genetic polymorphisms are related to individual variation in cancer susceptibility [10,11]. Susceptibility to cancer is determined by the activation of enzymes involved in carcinogen activation or deactivation. Such variations in genes encoding these enzymes could alter their expression and function, potentially influenced the balance between metabolic activation and detoxification of toxicants, leading to individual susceptibilities to cancer [12]. Two functional nonsynonymous polymorphisms have been recently studied. Specifically, a T-to-C mutation in the non-coding 3’-flanking region has been reported to cause the creation a new MspI restriction site (MspI polymorphism m1, T6235C, rs4646903). Another CYP1A1 polymorphism is a G-to-A transition (A4889G) in exon 7, resulting in the replacement of isoleucine (Ile) by valine (Val), which is a heme-binding site (Ile462Val polymorphism m2, A4889G, rs1048943) [13].

To date, a number of meta-analyses have been performed to explore the association between the MspI and Ile462Val polymorphisms of CYP1A1 and various cancers including prostate, ovarian, breast, lung, and colorectal cancer and leukemia, to name a few that occur in different ethnic populations [5,7,14-18]. However, a meta-analysis to investigate the relationship between CYP1A1 MspI and Ile462Val polymorphisms and overall cancer risk has not been performed. In the past twenty years, many molecular epidemiological studies have been conducted to investigate the association between CYP1A1 polymorphisms and cancer risk in humans. However, individual study limitations contributed to divergent conclusions among them. Hence, we performed a meta-analysis of all eligible studies to derive a more precise estimation of the relationship between the MspI and Ile462Val polymorphisms of CYP1A1 and an overall cancer risk.

## Materials and Methods

### Identification and Eligibility of Relevant Studies

All papers regarding the association between CYP1A1 polymorphisms and cancer risk published up to December 31, 2012 were identified through comprehensive searches using the PubMed database with the following terms and keywords: ‘‘cytochrome P450 1A1”, ‘‘CYP1A1’’ and ‘‘polymorphism’’, ‘‘variation’’, ‘‘mutation’’ and these terms were also paired with ‘‘cancer’’, ‘‘tumor’’ and ‘‘carcinoma’’. The search was limited to human studies and English language papers.

### Inclusion Criteria

For inclusion, the studies must have met the following criteria: (a) having information on the evaluation of the CYP1A1 polymorphism and cancer risk, (b) using a case-control design, and (c) containing complete information about all genotype frequencies. Exclusion criteria included (a) studies not focused on cancer or CYP1A1 research, (b) reviews, (c) reports without usable data, and (d) duplicate publications.

### Data Extraction

Information was carefully extracted from all qualified publications independently by two researchers (K Liu and SQ Wang) based on the inclusion criteria listed above. Disagreements were resolved through a discussion between the two researchers. The following information was collected from each included study using a standardized data collection protocol (Checklist **S1**): the first author’s name, the year of publication, ethnicity, country of origin, cancer type, genotyping method, source of control groups (population-based or hospital-based controls) and complete statistical data of all genotypes. Different ethnic descents were categorized as African, Asian, Caucasian, or Mixed (composed of different ethnic groups). Meanwhile, different case-controlled groups in one study were considered as independent studies.

### Statistical Methods

OR (odds ratios) and their 95% CIs (confidence intervals) were used to determine the strength of association between CYP1A1 MspI and Ile462Val polymorphisms and cancer risk. The percentage weight determined by the precision of its estimate of effect and, in the statistical software in STATA, is equal to the inverse of the variance. The risks (ORs) of cancer associated with CYP1A1 MspI and Ile462Val polymorphisms were estimated for each study. The statistical significance of the summary OR was determined with the Z-test. In our meta-analysis, we examined the association between allele C of the MspI polymorphism and the risk of cancer compared to that of allele T. Also, additive (CC vs. TT and CC vs. CT), recessive (CC vs. CT + TT), and dominant (CC + CT vs. TT) genetic models were investigated. The same method was applied to the Ile462Val polymorphism. Stratified analyses were also performed with respect to cancer type (if one cancer type contained less than two individual studies, it was classified as “other cancer”), ethnicity, source of controls and sample size (subjects exceeding 500 individuals in both case and control groups or not). Heterogeneity analysis was confirmed by the Chi-square-based Q-test. A P-value greater than 0.10 for the Q-test indicated a lack of heterogeneity among the studies, and then the fixed-effects model (the Mantel–Haenszel method) was used to calculate the summary OR estimate of each study. Otherwise, the random effects model (DerSimonian and Laird method) was used. The HWE in the control group was estimated by Fisher’s exact test and a P-value<0.05 was considered significant. An estimate of potential publication bias was performed using a funnel plot, in which the standard error of log (OR) of each study was plotted against its log (OR). An asymmetric plot suggests a possible publication bias. Funnel plot asymmetry was assessed by the method of Egger’s linear regression test, a linear regression approach to measure funnel plot asymmetry on the natural logarithmic scale of the OR. All statistical analyses were performed with the Stata software (version 12.1; StataCorp LP, College Station, TX), using two-sided P-values.

## Results

### Characteristics of Studies

**Figure 1:**
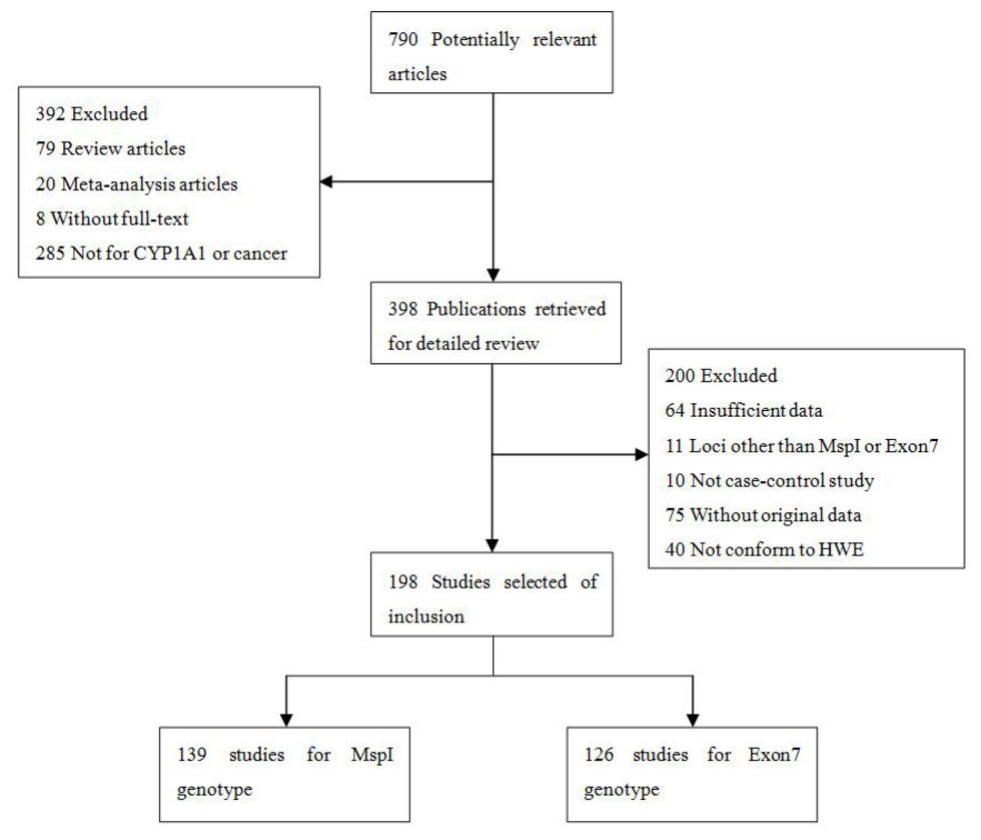
Studies identified with criteria for inclusion and exclusion.

Seven hundred and ninety potentially relevant citations were reviewed, and 198 publications (139 publications with 148 case-controlled studies for MspI and 126 publications with 134 case-controlled studies for Ile462Val) met the inclusion criteria and were selected in our meta-analysis. The study search process is depicted in Figure **1**. Detailed information about the 198 publications is listed in File **S1**.

In terms of the MspI polymorphism, 37783 cases and 50536 controls from 148 case-controlled studies were available. Study characteristics are summarized in Table **S3**. Among the 148 case–control studies, there were 55 studies of Caucasians, 59 studies of Asians, 7 studies of Africans and 27 studies of mixed descendents. There were 32 lung cancer studies, 29 breast cancer studies, 15 leukemia studies, 10 prostate cancer studies, 10 head and neck cancer studies, 9 colorectal cancer studies, 7 endometrial cancer studies, 6 cervical cancer studies, 5 ovarian cancer studies, 4 gastric cancer studies, 4 esophageal carcinoma studies, 3 hepatocellular cancer studies, 3 lymphoma studies and other cancers were categorized into the “others” group. Concerning source of controls, 74 were hospital based, 70 were population based and 4 were mixed. Furthermore, 46 studies were conducted with subjects≥500 in both case and control groups.

With respect to the Ile462Val polymorphism, 134 case–controlled studies were eligible (34466 cases and 44371 controls), comprising 67 studies with Asian populations, 45 studies with Caucasian populations, 3 studies with African populations and 19 studies with mixed populations. 52 studies were conducted on general populations, 52 were hospital based and 3 were mixed. There were 26 lung cancer studies, 25 breast cancer studies, 8 leukemia studies, 9 prostate cancer studies, 12 head and neck cancer studies, 8 colorectal cancer studies, 6 endometrial cancer studies, 3 cervical cancer studies, 4 ovarian cancer studies, 4 gastric cancer studies, 10 esophageal carcinoma studies, 3 hepatocellular cancer studies, 6 oral cancer studies and other cancers were categorized into the “others” group. Among the 134 case-control studies, 42 studies included a sample size≥500. Detailed study characteristics are summarized in Table **S4**.

Regarding genotyping methods, PCR–RFLP, TaqMan and allele-specific PCR methods were commonly used. For most studies, cancers were confirmed histologically or pathologically and all controls were chiefly matched for sex and age. In addition, the distribution of genotypes in the controls of all eligible studies was consistent with the Hardy-Weinberg equilibrium (HWE). 

### Quantitative Synthesis

The relationship between the MspI polymorphism and the risk of different kinds of cancer is summarized in Table **S1**. Overall, a significantly elevated risk of cancer was associated with the CYP1A1 C/C polymorphism for the allele contrast (C vs. T: OR = 1.15 CI = 1.09-1.22), the additive genetic model (C/C vs. T/T: OR = 1.33 CI = 1.17–1.51; C/C vs. C/T: OR = 1.14 CI = 1.03–1.27), the recessive genetic model (C/C vs. C/T+T/T: OR = 1.24 CI = 1.11–1.39) and the dominant genetic model(C/C + C/T vs. T/T: OR = 1.17 CI = 1.10–1.24). In the subgroup analysis by ethnicity, the results indicated that individuals with C/C genotype had a significantly higher cancer risk among Asians (C/C vs. T/T: OR = 1.45, CI = 1.24–1.69; C/C vs. C/T: OR = 1.17, CI = 1.04–1.32; recessive model: OR = 1.30, CI = 1.14–1.49; dominant model: OR = 1.26, CI = 1.13–1.39). When restricting the analysis to the source of controls, significant associations were found in mixed group (C/C vs. T/T: OR = 1.95, CI = 1.32–2.87; C/C vs. C/T: OR = 1.41, CI = 1.00–1.97; dominant model: OR= 1.43, CI = 1.19–1.71; recessive model: OR = 1.67, CI = 1.13–2.46). In the stratified analysis by cancer types, significant associations were found for lung cancer (C/C vs. T/T: OR = 1.43, CI = 1.16–1.78; dominant model: OR = 1.21, CI = 1.10–1.32; recessive model: OR = 1.32, CI = 1.07–1.62), cervical cancer (C/C vs. T/T: OR = 3.12, CI = 1.39–6.99; C/C vs. C/T: OR = 1.79, CI = 1.11–2.88; recessive model: OR = 2.48, CI = 1.41–4.36). In the stratified analysis by sample size (both cases and controls), significant associations were found for <500 (C/C vs. T/T: OR= 1.44, CI = 1.21–1.72; C/C vs. C/T: OR = 1.22, CI = 1.05–1.41; dominant model: OR = 1.23, CI = 1.12–1.36; recessive model: OR = 1.33, CI = 1.14–1.56).

Concerning the Ile462Val polymorphism data: the pooled ORs, along with their 95% CIs, are presented in detail in Table **S2**. Overall, a significantly increased risk of cancer was associated with the CYP1A1 G/G polymorphism for the allele contrast (G vs. A: OR = 1.18 CI = 1.12–1.25), the additive genetic model (G/G vs. A/A: OR =1.52 CI = 1.34–1.72; G/G vs. G/A: OR = 1.28 CI = 1.17–1.39), the recessive genetic model (G/G vs. G/A+A/A: OR = 1.42 CI = 1.27–1.60) and the dominant genetic model (G/G + G/A vs. A/A: OR = 1.18 CI = 1.11–1.26). A further analysis was performed on data stratified by ethnicity and an increased susceptibility was found in individuals with G/G genotype among Caucasians (G/G vs. A/A: OR = 1.90 CI = 1.45–2.51; G/G vs. G/A: OR = 1.53 CI = 1.19–1.99; dominant model: OR= 1.17 CI = 1.03–1.33; recessive model: OR= 1.74 CI = 1.34–2.26). Similar results were also observed among the Asians (G/G vs. A/A: OR = 1.46 CI = 1.26–1.68; G/G vs. G/A: OR = 1.24 CI = 1.12–1.36; dominant model: OR= 1.19 CI = 1.10–1.28; recessive model: OR= 1.38 CI = 1.21–1.57). In the stratified analysis by source of controls, significant associations were detected in all genetic models of HB (G/G vs. A/A: OR = 1.50 CI = 1.31–1.71; G/G vs. G/A: OR = 1.35 CI = 1.21–1.51; dominant model: OR = 1.15 CI = 1.07–1.25; recessive model: OR = 1.43 CI = 1.27–1.61) and PB (G/G vs. A/A: OR = 1.63 CI = 1.25–2.12; G/G vs. G/A: OR = 1.18 CI = 1.02–1.36; dominant model: OR = 1.22 CI = 1.08–1.38; recessive model: OR= 1.48 CI = 1.17–1.88). When restricting the analysis to cancer types, significant risks were found for lung cancer, leukemia, esophageal carcinoma and prostate cancer in all genetic models. Furthermore, in the stratified analysis according to sample size, the association was insignificant when the meta-analysis was restricted to larger studies. The per-allele OR of the G variant for more than 500 subjects was 1.05 (95% CI: 0.99–1.10), with corresponding results under dominant and recessive genetic models of 1.04 (95% CI: 0.98–1.11) and 1.15 (95% CI: 1.00–1.32), respectively.

### Test for Heterogeneity

Take the CYP1A1 Ile462Val genotype for example, there was significant heterogeneity for allele contrast (G vs. A: P <0.001), the additive genetic model comparison (G/G vs. G/A: P <0.001 and G/G vs. A/A: P <0.001), the dominant model comparison (G/G+G/A vs. A/A: P <0.001), and the recessive model comparison (G/G vs. G/A+A/A: P <0.001). Using a meta-regression analysis to explore the source of heterogeneity for dominant model comparisons (G/G+G/A vs. A/A) by ethnicity, cancer types, source of controls, and sample size, we observed that sample size (t = -3.3, P = 0.001) contributed to substantially altered heterogeneity, which could account for 100% of the heterogeneity source. Simultaneously, we found that cancer types (t = 0.58, P = 0.563), ethnicity (t =0.62, P = 0.534), or source of controls (t = -0.42, P = 0.677) did not contribute to the source of heterogeneity.

### Sensitivity Analysis

The sensitivity analysis was conducted by abandoning certain studies, such as the study that did not conform to HWE, the HWE in the control group was estimated by Fisher’s exact test. Studies before and after the process of individual study omission which had a P-value<0.05 were determined not to conform to HWE. After individual study omission, the corresponding pooled OR was not altered significantly. Sensitivity analysis thus confirmed that the meta-analysis results were statistically robust and that our results were reliable and stable.

### Publication Bias

**Figure 2:**
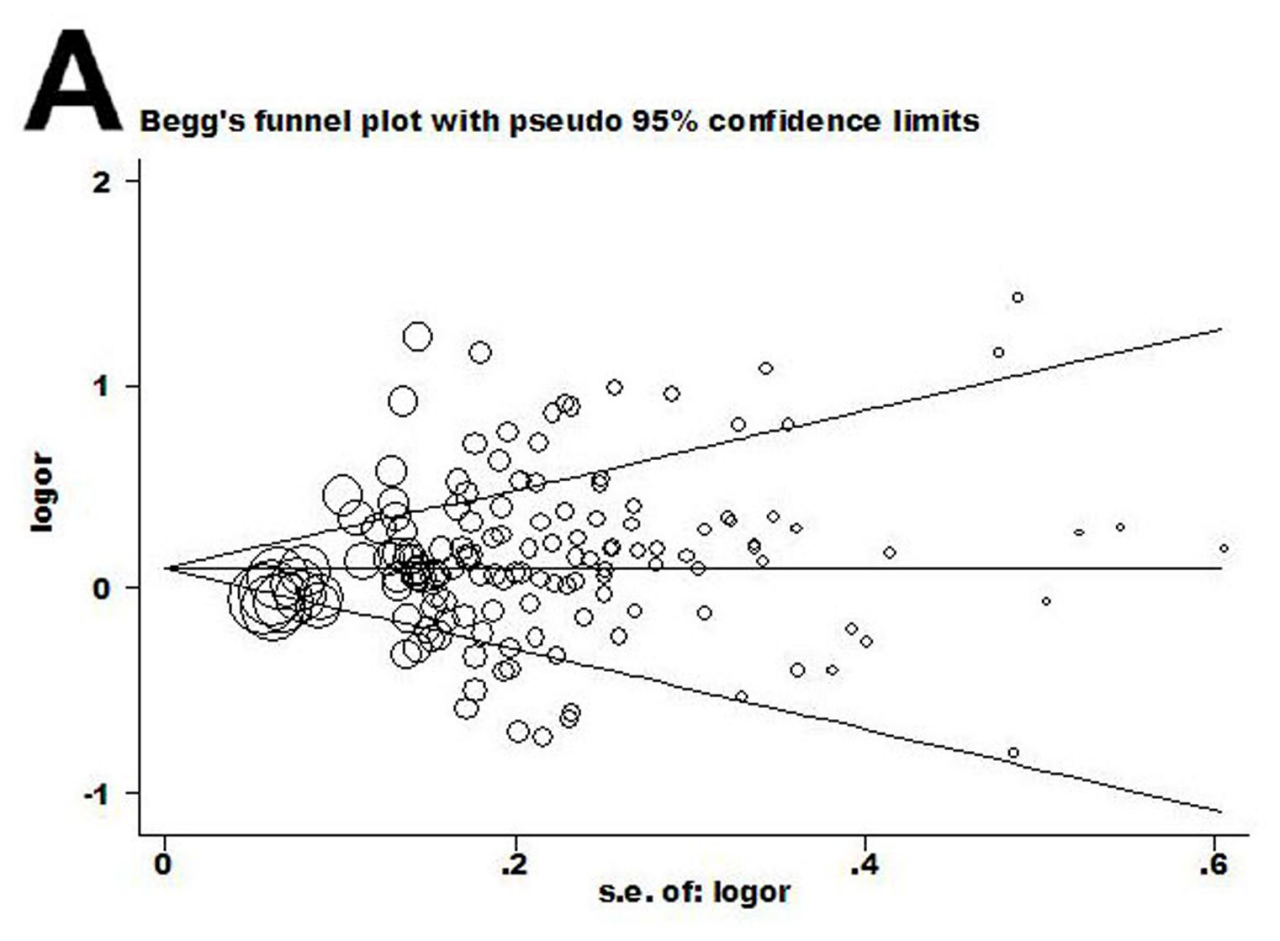
Begg’s funnel plot of publication bias in rs4646903 studies. (A) C allele vs. T allele. Each point represents a separate study for the indicated association. Log (OR), natural logarithm of OR. Horizontal line = mean effect size.

**Figure 3:**
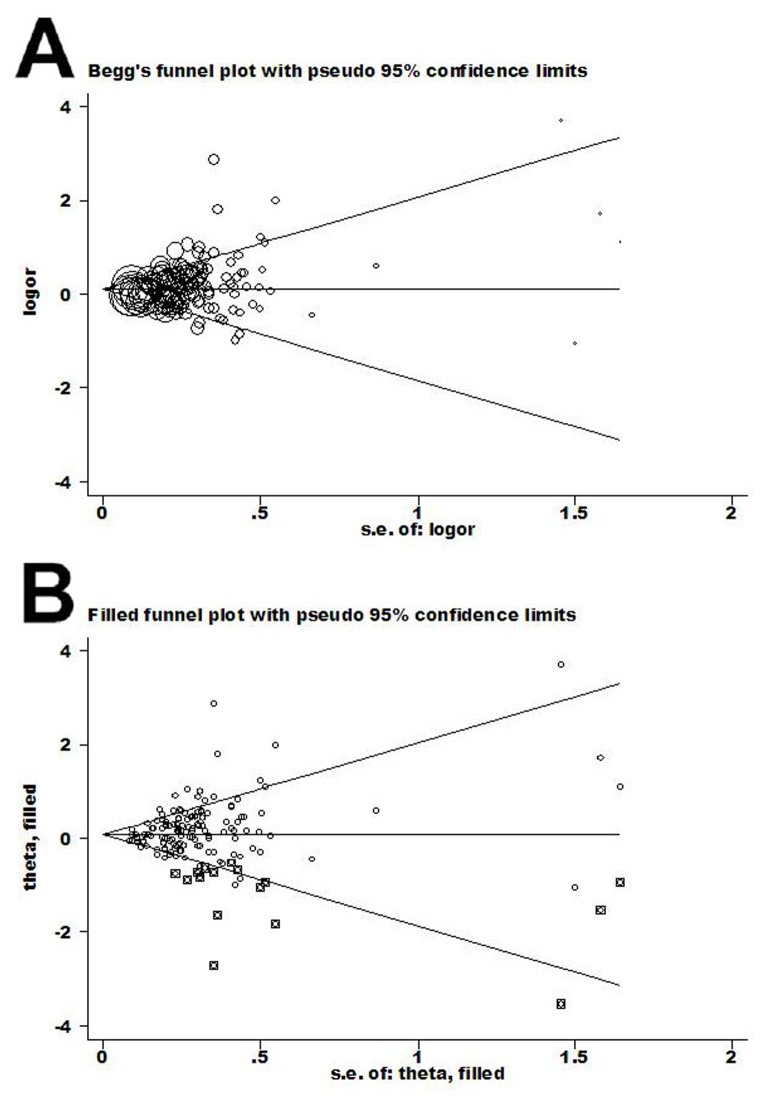
Begg’s funnel plot of publication bias in rs1048943 studies. (A) G/G+G/A vs. G/A. (B) trim-and-fill G/G+G/A vs. G/A. Each point represents a separate study for the indicated association. Log (OR), natural logarithm of OR. Horizontal line = mean effect size.

Begg’s funnel plot and Egger’s test were performed to evaluate the literature publication bias. Regarding the CYP1A1 MspI polymorphism (Figure **2**), the funnel plot shape for comparisons of the C and T allele of the CYP1A1 MspI polymorphism appeared symmetrical in all compared models. Then, the Egger’s test was adopted to provide statistical evidence of funnel plot symmetry. Data did not suggest evidence of publication bias (P = 0.232 for CC versus TT). For the Ile462Val genotype (Figure **3**), the shapes of the funnel plots seemed unsymmetrical in the dominant genetic model. However, the dominant model (G/G + G/A vs. A/A) had significant publication bias (t = 3.20 and P = 0.002). To adjust for this bias, a trim-and-fill method developed by Duval and Tweedie [19] was used to both identify and correct for funnel plot asymmetry arising from publication bias. We filled in the asymmetric outlying component of the funnel after estimating how many studies were in the asymmetric component using Stata software. Data revealed that 17 studies should be filled after iterations. We then estimated the true center of the funnel, the true mean, and 95% CI, based on the filled funnel plot. OR estimates and 95% CI in the fixed-effect model before and after trim-and-fill were 1.071, (1.034–1.110) and 1.084, (1.006–1.167). Also, for random-effect model, the results were 1.119, (1.079–1.160) and 1.181, (1.106–1.261). Meta-analysis with or without the trim-and-fill method did not offer different conclusions, indicating that our results were statistically robust.

## Discussion

CYP genes which are comprised of large families of endoplasmic and cytosolic enzymes, play a role in drug, steroid hormone, and procarcinogen metabolism. In humans, the CYPP450s complex (metalloproteins) contains more than 15 different enzymes [20]. Some CYP heme-thiolate enzymes participate in the detoxification and formation of reactive intermediates of thousands of chemicals that can damage DNA, lipids, and proteins. CYP expression can also affect the production of molecules derived from arachidonic acid, and alter various downstream signal-transduction pathways. Such changes can be precursors to malignancy [21]. CYP1A1 is a critical CYPP450 and studies suggest that a CYP1A1 polymorphism may be a risk factor for several malignancies even in the face of its role in detoxification of environmental carcinogens and metabolic activation of dietary compounds that protect against cancer. Therefore, the contribution of CYP1A1 to cancer progression or prevention may depend on the balance of procarcinogen activation/detoxification and dietary extrahepatic metabolism [9].

Previous studies about CYP1A1 polymorphisms and cancer risk have been inconclusive. To clarify any association, we conducted a meta-analysis of 198 publications. To the best of our knowledge, this is the first meta-analysis to evaluate the relationship between CYP1A1 polymorphisms and overall cancer risk. Our study also provides a subgroup analysis stratified by ethnicity, source of control, cancer type and sample size. Our results indicated that the MspI polymorphism C/C genotype was associated with an increased risk of cancers, especially for lung and cervical cancer among Asians and mixed populations, whereas the Ile462Val polymorphism G/G genotype was associated with an increased risk of lung cancer, leukemia, esophageal carcinoma, and prostate cancer among Caucasians and Asians.

Because tumor origin could influence the results from meta-analyses, we performed subgroup analyses by cancer type. We found that CYP1A1 MspI and Ile462Val polymorphisms correlated with increased lung cancer susceptibility. Also, the MspI polymorphism C/C genotype is associated with an increased risk of cervical cancer. Interestingly, Gutman and co-workers [22] reported that the CYP1A1 MspI C/C polymorphism is unlikely to be a major risk factor for cervical cancer which is contrary to our data from the meta-analysis. Similarly, a marked and noteworthy conflict emerges between the meta-analysis data and recent data from Wideroff’s [23] and Li’s laboratory [24]. Both researchers reported that the Ile462Val G/G polymorphism is not associated with an increased risk of esophageal carcinoma and prostate cancer but our data indicate that this G/G polymorphism may affect susceptibility to these very kinds of cancer. Several discrepancies such as these were discovered although the reason for this is unclear. Perhaps different cancers with different carcinogenic mechanisms and environmental exposures had disparate responses to CYP1A1 genotypes. Also, for some cancer subtypes, only a few studies existed, and these had limited sample sizes. Thus, some studies may have been underpowered to detect small, but meaningful, associations. Consequently, large-scale, detailed and mechanistic studies are needed to confirm these relationships.

In the subgroup analysis by ethnicity, the MspI C/C polymorphism was found to confer an increased cancer risk among Asians and mixed population but not Caucasians or Africans. For the Ile462Val G/G polymorphism, statistically significantly elevated cancer risks were observed in Asians and Caucasians but not in Africans or in mixed descendant populations. The exact mechanism for the ethnic discrepancy is uncertain but differences in underlying genetic backgrounds and social factors among different populations studied may be important. Ethnically diverse subjects may have unique cultures and life-styles that can contribute to different genetic characteristics and susceptibility to specific cancers. Also, in this meta-analysis, the sample size and numbers of studies in African groups and mixed groups were not adequate to evaluate any association. Finally, selection bias, different matching criteria and misclassifications of disease status and genotyping may have contributed to the discrepancy. Overall, our data do suggest genetic diversity among different ethnicities.

Other limitations of this study included heterogeneity, which can interfere with the interpretation of meta-analysis data. Although we minimized this likelihood by performing a careful search of published studies, using explicit criteria for a study’s inclusion and performing strict data extraction and analysis, significant interstudy heterogeneity nevertheless existed in nearly every comparison. In our meta-analysis, the sample size of studies among Africans and among several cancer types is small and limited. As a result, the sample size accounted for most of the heterogeneity source. However, we did not exclude the possibility that ethnic or tumor type differences may contribute to the relatively large heterogeneity. Simultaneously, heterogeneity may arise from differences in the selection of controls, as well as subject age distribution and lifestyle factors. In addition, lack of original data from reviewed studies limited our evaluation of potential interactions because the interactions between gene-to-gene, gene-to-environment, and even different polymorphic loci of the same gene that may modulate cancer risks. Finally, the quantity of published studies was insufficient for a comprehensive analysis, particularly for single types of cancer and Africans. A better analysis would include detailed individual data such as age and sex. Therefore, more studies with sufficient sample sizes and detailed information are warranted. Also, studies indexed by the selected databases were included for the meta-analysis, and some relevant published studies or unpublished studies with null results may have been overlooked which would bias our results.

In conclusion, although significant heterogeneity from included studies existed, our meta-analysis provided evidence to support an association between the CYP1A1 MspI and Ile462Val polymorphisms and increased cancer risk. The effects of the two genotypes of each CYP1A1 polymorphism are diverse according to subgroup analysis stratified by ethnicity, cancer type, and source of control. In the future, strict selection of patients, well-matched controls, standardized and unbiased methods and larger sample sizes are essential. Gene-gene and gene-environment interactions should also be considered as well as ethnic-specific studies to investigate the role of the two functional polymorphisms in Africans and specific cancer types.

## Supporting Information

Checklist S1 **PRISMA 2009 Checklist.**
(DOC)

File S1 **All eligible articles involved in this meta-analysis.**
(DOC)

Table S1 **Stratification analyses of the P value and 95% confidence interval for MspI polymorphism.**
(DOC)

Table S2 **Stratification analyses of the P value and 95% confidence interval for Ile462Val polymorphism.**
(DOC)

Table S3 **Characteristics of studies included in the meta-analysis for MspI polymorphism.** A generalized distribution of MspI genotype frequencies for each included study is listed.
(DOC)

Table S4 **Characteristics of studies included in the meta-analysis for Ile462Val polymorphism.** A generalized distribution of Ile462Val genotype frequencies for each included studies is listed.
(DOC)
