# Supplementary material for: MspI and Ile462Val Polymorphisms in CYP1A1 and Overall Cancer Risk: A Meta-Analysis
Source: PLoS One. 2013 Dec 31;8(12):e85166. doi: 10.1371/journal.pone.0085166 (PMC3877352; doi:10.1371/journal.pone.0085166)
Supplement: File S1 — All eligible articles involved in this meta-analysis. (DOC) [file pone.0085166.s002.doc]

**All eligible articles involved in this meta-analysis**

1. [Park SK](http://www.ncbi.nlm.nih.gov/pubmed?term=Park SK%5BAuthor%5D&cauthor=true&cauthor_uid=19168589), [Andreotti G](http://www.ncbi.nlm.nih.gov/pubmed?term=Andreotti G%5BAuthor%5D&cauthor=true&cauthor_uid=19168589), [Sakoda LC](http://www.ncbi.nlm.nih.gov/pubmed?term=Sakoda LC%5BAuthor%5D&cauthor=true&cauthor_uid=19168589), [Gao YT](http://www.ncbi.nlm.nih.gov/pubmed?term=Gao YT%5BAuthor%5D&cauthor=true&cauthor_uid=19168589), [Rashid A](http://www.ncbi.nlm.nih.gov/pubmed?term=Rashid A%5BAuthor%5D&cauthor=true&cauthor_uid=19168589), et al. (2009) Variants in hormone-related genes and the risk of biliary tract cancers and stones: a population-based study in China. Carcinogenesis 30:606-614.
2. Ozturk T, Kahraman OT, Toptas B, Kisakesen HI, Cakalir C, et al. (2011) The effect of CYP1A1 and GSTM1 gene polymorphisms in bladder cancer development in a Turkish population. In Vivo 25:663-668.
3. [Srivastava DS](http://www.ncbi.nlm.nih.gov/pubmed?term=Srivastava DS%5BAuthor%5D&cauthor=true&cauthor_uid=18200441), [Mandhani A](http://www.ncbi.nlm.nih.gov/pubmed?term=Mandhani A%5BAuthor%5D&cauthor=true&cauthor_uid=18200441), [Mittal RD](http://www.ncbi.nlm.nih.gov/pubmed?term=Mittal RD%5BAuthor%5D&cauthor=true&cauthor_uid=18200441) (2008) Genetic polymorphisms of cytochrome P450 CYP1A1 (*2A) and microsomal epoxide hydrolase gene, interactions with tobacco-users, and susceptibility to bladder cancer: a study from North India. Arch Toxicol 82:633-639.
4. [Ambrosone CB](http://www.ncbi.nlm.nih.gov/pubmed?term=Ambrosone CB%5BAuthor%5D&cauthor=true&cauthor_uid=7627950), [Freudenheim JL](http://www.ncbi.nlm.nih.gov/pubmed?term=Freudenheim JL%5BAuthor%5D&cauthor=true&cauthor_uid=7627950), [Graham S](http://www.ncbi.nlm.nih.gov/pubmed?term=Graham S%5BAuthor%5D&cauthor=true&cauthor_uid=7627950), [Marshall JR](http://www.ncbi.nlm.nih.gov/pubmed?term=Marshall JR%5BAuthor%5D&cauthor=true&cauthor_uid=7627950), [Vena JE](http://www.ncbi.nlm.nih.gov/pubmed?term=Vena JE%5BAuthor%5D&cauthor=true&cauthor_uid=7627950), et al. (1995) Cytochrome P4501A1 and glutathione S-transferase (M1) genetic polymorphisms and postmenopausal breast cancer risk. Cancer Res 55:3483-3485.
5. [Bailey LR](http://www.ncbi.nlm.nih.gov/pubmed?term=Bailey LR%5BAuthor%5D&cauthor=true&cauthor_uid=9426059), [Roodi N](http://www.ncbi.nlm.nih.gov/pubmed?term=Roodi N%5BAuthor%5D&cauthor=true&cauthor_uid=9426059), [Verrier CS](http://www.ncbi.nlm.nih.gov/pubmed?term=Verrier CS%5BAuthor%5D&cauthor=true&cauthor_uid=9426059), [Yee CJ](http://www.ncbi.nlm.nih.gov/pubmed?term=Yee CJ%5BAuthor%5D&cauthor=true&cauthor_uid=9426059), [Dupont WD](http://www.ncbi.nlm.nih.gov/pubmed?term=Dupont WD%5BAuthor%5D&cauthor=true&cauthor_uid=9426059), et al. (1998) Breast cancer and CYPIA1, GSTM1, and GSTT1 polymorphisms: evidence of a lack of association in Caucasians and African Americans. Cancer Res 58:65-70.
6. [Basham VM](http://www.ncbi.nlm.nih.gov/pubmed?term=Basham VM%5BAuthor%5D&cauthor=true&cauthor_uid=11698341), [Pharoah PD](http://www.ncbi.nlm.nih.gov/pubmed?term=Pharoah PD%5BAuthor%5D&cauthor=true&cauthor_uid=11698341), [Healey CS](http://www.ncbi.nlm.nih.gov/pubmed?term=Healey CS%5BAuthor%5D&cauthor=true&cauthor_uid=11698341), [Luben RN](http://www.ncbi.nlm.nih.gov/pubmed?term=Luben RN%5BAuthor%5D&cauthor=true&cauthor_uid=11698341), [Day NE](http://www.ncbi.nlm.nih.gov/pubmed?term=Day NE%5BAuthor%5D&cauthor=true&cauthor_uid=11698341), et al. (2001) Polymorphisms in CYP1A1 and smoking: no association with breast cancer risk. Carcinogenesis 22:1797-1800.
7. [Boyapati SM](http://www.ncbi.nlm.nih.gov/pubmed?term=Boyapati SM%5BAuthor%5D&cauthor=true&cauthor_uid=15856430), [Shu XO](http://www.ncbi.nlm.nih.gov/pubmed?term=Shu XO%5BAuthor%5D&cauthor=true&cauthor_uid=15856430), [Gao YT](http://www.ncbi.nlm.nih.gov/pubmed?term=Gao YT%5BAuthor%5D&cauthor=true&cauthor_uid=15856430), [Cai Q](http://www.ncbi.nlm.nih.gov/pubmed?term=Cai Q%5BAuthor%5D&cauthor=true&cauthor_uid=15856430), [Jin F](http://www.ncbi.nlm.nih.gov/pubmed?term=Jin F%5BAuthor%5D&cauthor=true&cauthor_uid=15856430), et al. (2005) Polymorphisms in CYP1A1 and breast carcinoma risk in a population-based case-control study of Chinese women. [Cancer](http://www.ncbi.nlm.nih.gov/pubmed/?term=Polymorphisms+in+CYP1A1+and+breast+carcinoma+risk+in+a+population-based+case-control+study+of+Chinese+women) 103:2228-2235.
8. [Chacko P](http://www.ncbi.nlm.nih.gov/pubmed?term=Chacko P%5BAuthor%5D&cauthor=true&cauthor_uid=15725614), [Joseph T](http://www.ncbi.nlm.nih.gov/pubmed?term=Joseph T%5BAuthor%5D&cauthor=true&cauthor_uid=15725614), [Mathew BS](http://www.ncbi.nlm.nih.gov/pubmed?term=Mathew BS%5BAuthor%5D&cauthor=true&cauthor_uid=15725614), [Rajan B](http://www.ncbi.nlm.nih.gov/pubmed?term=Rajan B%5BAuthor%5D&cauthor=true&cauthor_uid=15725614), [Pillai MR](http://www.ncbi.nlm.nih.gov/pubmed?term=Pillai MR%5BAuthor%5D&cauthor=true&cauthor_uid=15725614) (2005) Role of xenobiotic metabolizing gene polymorphisms in breast cancer susceptibility and treatment outcome. [Mutat Res](http://www.ncbi.nlm.nih.gov/pubmed/?term=Role+of+xenobiotic+metabolizing+gene+polymorphisms+in+breast+cancer+susceptibility+and+treatment+outcome) 581:153-163.
9. [Hefler LA](http://www.ncbi.nlm.nih.gov/pubmed?term=Hefler LA%5BAuthor%5D&cauthor=true&cauthor_uid=15241822), [Tempfer CB](http://www.ncbi.nlm.nih.gov/pubmed?term=Tempfer CB%5BAuthor%5D&cauthor=true&cauthor_uid=15241822), [Grimm C](http://www.ncbi.nlm.nih.gov/pubmed?term=Grimm C%5BAuthor%5D&cauthor=true&cauthor_uid=15241822), [Lebrecht A](http://www.ncbi.nlm.nih.gov/pubmed?term=Lebrecht A%5BAuthor%5D&cauthor=true&cauthor_uid=15241822), [Ulbrich E](http://www.ncbi.nlm.nih.gov/pubmed?term=Ulbrich E%5BAuthor%5D&cauthor=true&cauthor_uid=15241822), et al. (2004) Estrogen-metabolizing gene polymorphisms in the assessment of breast carcinoma risk and fibroadenoma risk in Caucasian women. [Cancer](http://www.ncbi.nlm.nih.gov/pubmed/?term=Estrogen-metabolizing+gene+polymorphisms+in+the+assessment+of+breast+carcinoma+risk+and+fibroadenoma+risk+in+Caucasian+women) 101:264-269.
10. [Huang CS](http://www.ncbi.nlm.nih.gov/pubmed?term=Huang CS%5BAuthor%5D&cauthor=true&cauthor_uid=10468307), [Shen CY](http://www.ncbi.nlm.nih.gov/pubmed?term=Shen CY%5BAuthor%5D&cauthor=true&cauthor_uid=10468307), [Chang KJ](http://www.ncbi.nlm.nih.gov/pubmed?term=Chang KJ%5BAuthor%5D&cauthor=true&cauthor_uid=10468307), [Hsu SM](http://www.ncbi.nlm.nih.gov/pubmed?term=Hsu SM%5BAuthor%5D&cauthor=true&cauthor_uid=10468307), [Chern HD](http://www.ncbi.nlm.nih.gov/pubmed?term=Chern HD%5BAuthor%5D&cauthor=true&cauthor_uid=10468307) (1999) Cytochrome P4501A1 polymorphism as a susceptibility factor for breast cancer in postmenopausal Chinese women in Taiwan. [Br J Cancer](http://www.ncbi.nlm.nih.gov/pubmed/?term=Cytochrome+P4501A1+polymorphism+as+a+susceptibility+factor+for+breast+cancer+in+postmenopausal+Chinese+women+in+Taiwan) 80:1838-1843.
11. [Justenhoven C](http://www.ncbi.nlm.nih.gov/pubmed?term=Justenhoven C%5BAuthor%5D&cauthor=true&cauthor_uid=17588204), [Hamann U](http://www.ncbi.nlm.nih.gov/pubmed?term=Hamann U%5BAuthor%5D&cauthor=true&cauthor_uid=17588204), [Schubert F](http://www.ncbi.nlm.nih.gov/pubmed?term=Schubert F%5BAuthor%5D&cauthor=true&cauthor_uid=17588204), [Zapatka M](http://www.ncbi.nlm.nih.gov/pubmed?term=Zapatka M%5BAuthor%5D&cauthor=true&cauthor_uid=17588204), [Pierl CB](http://www.ncbi.nlm.nih.gov/pubmed?term=Pierl CB%5BAuthor%5D&cauthor=true&cauthor_uid=17588204), et al. (2008) Breast cancer: a candidate gene approach across the estrogen metabolic pathway. [Breast Cancer Res Treat](http://www.ncbi.nlm.nih.gov/pubmed/?term=Breast+cancer%3A+a+candidate+gene+approach+across+the+estrogen+metabolic+pathway) 108:137-149.
12. [Kato I](http://www.ncbi.nlm.nih.gov/pubmed?term=Kato I%5BAuthor%5D&cauthor=true&cauthor_uid=19679043), [Cichon M](http://www.ncbi.nlm.nih.gov/pubmed?term=Cichon M%5BAuthor%5D&cauthor=true&cauthor_uid=19679043), [Yee CL](http://www.ncbi.nlm.nih.gov/pubmed?term=Yee CL%5BAuthor%5D&cauthor=true&cauthor_uid=19679043), [Land S](http://www.ncbi.nlm.nih.gov/pubmed?term=Land S%5BAuthor%5D&cauthor=true&cauthor_uid=19679043), [Korczak JF](http://www.ncbi.nlm.nih.gov/pubmed?term=Korczak JF%5BAuthor%5D&cauthor=true&cauthor_uid=19679043) (2009) African American-preponderant single nucleotide polymorphisms (SNPs) and risk of breast cancer. [Cancer Epidemiol](http://www.ncbi.nlm.nih.gov/pubmed/?term=African+American-preponderant+single+nucleotide+polymorphisms+(SNPs)+and+risk+of+breast+cancer) 33:24-30.
13. [Khvostova EP](http://www.ncbi.nlm.nih.gov/pubmed?term=Khvostova EP%5BAuthor%5D&cauthor=true&cauthor_uid=21977969), [Pustylnyak VO](http://www.ncbi.nlm.nih.gov/pubmed?term=Pustylnyak VO%5BAuthor%5D&cauthor=true&cauthor_uid=21977969), [Gulyaeva LF](http://www.ncbi.nlm.nih.gov/pubmed?term=Gulyaeva LF%5BAuthor%5D&cauthor=true&cauthor_uid=21977969) (2012) Genetic polymorphism of estrogen metabolizing enzymes in Siberian women with breast cancer. [Genet Test Mol Biomarkers](http://www.ncbi.nlm.nih.gov/pubmed/?term=Genetic+polymorphism+of+estrogen+metabolizing+enzymes+in+Siberian+women+with+breast+cancer) 16:167-173.
14. [Kiruthiga PV](http://www.ncbi.nlm.nih.gov/pubmed?term=Kiruthiga PV%5BAuthor%5D&cauthor=true&cauthor_uid=22292665), [Kannan MR](http://www.ncbi.nlm.nih.gov/pubmed?term=Kannan MR%5BAuthor%5D&cauthor=true&cauthor_uid=22292665), [Saraswathi C](http://www.ncbi.nlm.nih.gov/pubmed?term=Saraswathi C%5BAuthor%5D&cauthor=true&cauthor_uid=22292665), [Pandian SK](http://www.ncbi.nlm.nih.gov/pubmed?term=Pandian SK%5BAuthor%5D&cauthor=true&cauthor_uid=22292665), [Devi KP](http://www.ncbi.nlm.nih.gov/pubmed?term=Devi KP%5BAuthor%5D&cauthor=true&cauthor_uid=22292665) (2011) CYP1A1 gene polymorphisms: lack of association with breast cancer susceptibility in the southern region (Madurai) of India. [Asian Pac J Cancer Prev](http://www.ncbi.nlm.nih.gov/pubmed/?term=CYP1A1+gene+polymorphisms%3A+lack+of+association+with+breast+cancer+susceptibility+in+the+southern+region+(Madurai)+of+India) 12:2133-2138.
15. [Krajinovic M](http://www.ncbi.nlm.nih.gov/pubmed?term=Krajinovic M%5BAuthor%5D&cauthor=true&cauthor_uid=11291049), [Ghadirian P](http://www.ncbi.nlm.nih.gov/pubmed?term=Ghadirian P%5BAuthor%5D&cauthor=true&cauthor_uid=11291049), [Richer C](http://www.ncbi.nlm.nih.gov/pubmed?term=Richer C%5BAuthor%5D&cauthor=true&cauthor_uid=11291049), [Sinnett H](http://www.ncbi.nlm.nih.gov/pubmed?term=Sinnett H%5BAuthor%5D&cauthor=true&cauthor_uid=11291049), [Gandini S](http://www.ncbi.nlm.nih.gov/pubmed?term=Gandini S%5BAuthor%5D&cauthor=true&cauthor_uid=11291049), et al. (2001) Genetic susceptibility to breast cancer in French-Canadians: role of carcinogen-metabolizing enzymes and gene-environment interactions. [Int J Cancer](http://www.ncbi.nlm.nih.gov/pubmed/?term=Genetic+susceptibility+to+breast+cancer+in+French-Canadians%3A+role+of+carcinogen-metabolizing+enzymes+and+gene-environment+interactions) 92:220-225.
16. [Le Marchand L](http://www.ncbi.nlm.nih.gov/pubmed?term=Le Marchand L%5BAuthor%5D&cauthor=true&cauthor_uid=16103451), [Donlon T](http://www.ncbi.nlm.nih.gov/pubmed?term=Donlon T%5BAuthor%5D&cauthor=true&cauthor_uid=16103451), [Kolonel LN](http://www.ncbi.nlm.nih.gov/pubmed?term=Kolonel LN%5BAuthor%5D&cauthor=true&cauthor_uid=16103451), [Henderson BE](http://www.ncbi.nlm.nih.gov/pubmed?term=Henderson BE%5BAuthor%5D&cauthor=true&cauthor_uid=16103451), [Wilkens LR](http://www.ncbi.nlm.nih.gov/pubmed?term=Wilkens LR%5BAuthor%5D&cauthor=true&cauthor_uid=16103451) (2005) Estrogen metabolism-related genes and breast cancer risk: the multiethnic cohort study. [Cancer Epidemiol Biomarkers Prev](http://www.ncbi.nlm.nih.gov/pubmed/?term=Estrogen+metabolism-related+genes+and+breast+cancer+risk%3A+the+multiethnic+cohort+study) 14:1998-2003.
17. [Li Y](http://www.ncbi.nlm.nih.gov/pubmed?term=Li Y%5BAuthor%5D&cauthor=true&cauthor_uid=15642161), [Millikan RC](http://www.ncbi.nlm.nih.gov/pubmed?term=Millikan RC%5BAuthor%5D&cauthor=true&cauthor_uid=15642161), [Bell DA](http://www.ncbi.nlm.nih.gov/pubmed?term=Bell DA%5BAuthor%5D&cauthor=true&cauthor_uid=15642161), [Cui L](http://www.ncbi.nlm.nih.gov/pubmed?term=Cui L%5BAuthor%5D&cauthor=true&cauthor_uid=15642161), [Tse CK](http://www.ncbi.nlm.nih.gov/pubmed?term=Tse CK%5BAuthor%5D&cauthor=true&cauthor_uid=15642161), et al. (2005) Polychlorinated biphenyls, cytochrome P450 1A1 (CYP1A1) polymorphisms, and breast cancer risk among African American women and white women in North Carolina: a population-based case-control study. [Breast Cancer Res](http://www.ncbi.nlm.nih.gov/pubmed/?term=Polychlorinated+biphenyls%2C+cytochrome+P450+1A1+(CYP1A1)+polymorphisms%2C+and+breast+cancer+risk+among+African+American+women+and+white+women+in+North+Carolina%3A+a+population-based+case-control+study) 7:R12-18.
18. [MARIE-GENICA Consortium on Genetic Susceptibility for Menopausal Hormone Therapy Related Breast Cancer Risk](http://www.ncbi.nlm.nih.gov/pubmed?term=MARIE-GENICA Consortium on Genetic Susceptibility for Menopausal Hormone Therapy Related Breast Cancer Risk%5BCorporate Author%5D) (2010) Genetic polymorphisms in phase I and phase II enzymes and breast cancer risk associated with menopausal hormone therapy in postmenopausal women. [Breast Cancer Res Treat](http://www.ncbi.nlm.nih.gov/pubmed/?term=Genetic+polymorphisms+in+phase+I+and+phase+II+enzymes+and+breast+cancer+risk+associated+with+menopausal+hormone+therapy+in+postmenopausal+women) 119:463-474.
19. [Miyoshi Y](http://www.ncbi.nlm.nih.gov/pubmed?term=Miyoshi Y%5BAuthor%5D&cauthor=true&cauthor_uid=12100112), [Takahashi Y](http://www.ncbi.nlm.nih.gov/pubmed?term=Takahashi Y%5BAuthor%5D&cauthor=true&cauthor_uid=12100112), [Egawa C](http://www.ncbi.nlm.nih.gov/pubmed?term=Egawa C%5BAuthor%5D&cauthor=true&cauthor_uid=12100112), [Noguchi S](http://www.ncbi.nlm.nih.gov/pubmed?term=Noguchi S%5BAuthor%5D&cauthor=true&cauthor_uid=12100112) (2002) Breast cancer risk associated with CYP1A1 genetic polymorphisms in Japanese women. [Breast J](http://www.ncbi.nlm.nih.gov/pubmed/12100112) 8:209-215.
20. [Moreno-Galván M](http://www.ncbi.nlm.nih.gov/pubmed?term=Moreno-Galván M%5BAuthor%5D&cauthor=true&cauthor_uid=20878621), [Herrera-González NE](http://www.ncbi.nlm.nih.gov/pubmed?term=Herrera-González NE%5BAuthor%5D&cauthor=true&cauthor_uid=20878621), [Robles-Pérez V](http://www.ncbi.nlm.nih.gov/pubmed?term=Robles-Pérez V%5BAuthor%5D&cauthor=true&cauthor_uid=20878621), [Velasco-Rodríguez JC](http://www.ncbi.nlm.nih.gov/pubmed?term=Velasco-Rodríguez JC%5BAuthor%5D&cauthor=true&cauthor_uid=20878621), [Tapia-Conyer R](http://www.ncbi.nlm.nih.gov/pubmed?term=Tapia-Conyer R%5BAuthor%5D&cauthor=true&cauthor_uid=20878621), et al. (2010) Impact of CYP1A1 and COMT genotypes on breast cancer risk in Mexican women: a pilot study. [Int J Biol Markers](http://www.ncbi.nlm.nih.gov/pubmed/?term=30.%09Impact+of+CYP1A1+and+COMT+genotypes+on+breast+cancer+risk+in+Mexican+women%3A+a+pilot+study.) 25:157-163.
21. [Naushad SM](http://www.ncbi.nlm.nih.gov/pubmed?term=Naushad SM%5BAuthor%5D&cauthor=true&cauthor_uid=21792634), [Reddy CA](http://www.ncbi.nlm.nih.gov/pubmed?term=Reddy CA%5BAuthor%5D&cauthor=true&cauthor_uid=21792634), [Rupasree Y](http://www.ncbi.nlm.nih.gov/pubmed?term=Rupasree Y%5BAuthor%5D&cauthor=true&cauthor_uid=21792634), [Pavani A](http://www.ncbi.nlm.nih.gov/pubmed?term=Pavani A%5BAuthor%5D&cauthor=true&cauthor_uid=21792634), [Digumarti RR](http://www.ncbi.nlm.nih.gov/pubmed?term=Digumarti RR%5BAuthor%5D&cauthor=true&cauthor_uid=21792634), et al. (2010) Cross-talk between one-carbon metabolism and xenobiotic metabolism: implications on oxidative DNA damage and susceptibility to breast cancer. [Cell Biochem Biophys](http://www.ncbi.nlm.nih.gov/pubmed/?term=Cross-talk+between+one-carbon+metabolism+and+xenobiotic+metabolism%3A+implications+on+oxidative+DNA+damage+and+susceptibility+to+breast+cancer) 61:715-723.
22. [Ociepa-Zawal M](http://www.ncbi.nlm.nih.gov/pubmed?term=Ociepa-Zawal M%5BAuthor%5D&cauthor=true&cauthor_uid=20088394), [Rubiś B](http://www.ncbi.nlm.nih.gov/pubmed?term=Rubiś B%5BAuthor%5D&cauthor=true&cauthor_uid=20088394), [Filas V](http://www.ncbi.nlm.nih.gov/pubmed?term=Filas V%5BAuthor%5D&cauthor=true&cauthor_uid=20088394), [Breborowicz J](http://www.ncbi.nlm.nih.gov/pubmed?term=Breborowicz J%5BAuthor%5D&cauthor=true&cauthor_uid=20088394), [Trzeciak WH](http://www.ncbi.nlm.nih.gov/pubmed?term=Trzeciak WH%5BAuthor%5D&cauthor=true&cauthor_uid=20088394) (2009) Studies on CYP1A1, CYP1B1 and CYP3A4 gene polymorphisms in breast cancer patients. [Ginekol Pol](http://www.ncbi.nlm.nih.gov/pubmed/20088394) 80:819-823.
23. [Okobia M](http://www.ncbi.nlm.nih.gov/pubmed?term=Okobia M%5BAuthor%5D&cauthor=true&cauthor_uid=16254684), [Bunker C](http://www.ncbi.nlm.nih.gov/pubmed?term=Bunker C%5BAuthor%5D&cauthor=true&cauthor_uid=16254684), [Zmuda J](http://www.ncbi.nlm.nih.gov/pubmed?term=Zmuda J%5BAuthor%5D&cauthor=true&cauthor_uid=16254684), [Kammerer C](http://www.ncbi.nlm.nih.gov/pubmed?term=Kammerer C%5BAuthor%5D&cauthor=true&cauthor_uid=16254684), [Vogel V](http://www.ncbi.nlm.nih.gov/pubmed?term=Vogel V%5BAuthor%5D&cauthor=true&cauthor_uid=16254684), et al. (2005) Cytochrome P4501A1 genetic polymorphisms and breast cancer risk in Nigerian women. [Breast Cancer Res Treat](http://www.ncbi.nlm.nih.gov/pubmed/?term=Cytochrome+P4501A1+genetic+polymorphisms+and+breast+cancer+risk+in+Nigerian+women) 94:285-293.
24. [Sangrajrang S](http://www.ncbi.nlm.nih.gov/pubmed?term=Sangrajrang S%5BAuthor%5D&cauthor=true&cauthor_uid=19415745), [Sato Y](http://www.ncbi.nlm.nih.gov/pubmed?term=Sato Y%5BAuthor%5D&cauthor=true&cauthor_uid=19415745), [Sakamoto H](http://www.ncbi.nlm.nih.gov/pubmed?term=Sakamoto H%5BAuthor%5D&cauthor=true&cauthor_uid=19415745), [Ohnami S](http://www.ncbi.nlm.nih.gov/pubmed?term=Ohnami S%5BAuthor%5D&cauthor=true&cauthor_uid=19415745), [Laird NM](http://www.ncbi.nlm.nih.gov/pubmed?term=Laird NM%5BAuthor%5D&cauthor=true&cauthor_uid=19415745), et al. (2009) Genetic polymorphisms of estrogen metabolizing enzyme and breast cancer risk in Thai women. [Int J Cancer](http://www.ncbi.nlm.nih.gov/pubmed/19415745) 125:837-843.
25. [Shen Y](http://www.ncbi.nlm.nih.gov/pubmed?term=Shen Y%5BAuthor%5D&cauthor=true&cauthor_uid=16492926), [Li DK](http://www.ncbi.nlm.nih.gov/pubmed?term=Li DK%5BAuthor%5D&cauthor=true&cauthor_uid=16492926), [Wu J](http://www.ncbi.nlm.nih.gov/pubmed?term=Wu J%5BAuthor%5D&cauthor=true&cauthor_uid=16492926), [Zhang Z](http://www.ncbi.nlm.nih.gov/pubmed?term=Zhang Z%5BAuthor%5D&cauthor=true&cauthor_uid=16492926), [Gao E](http://www.ncbi.nlm.nih.gov/pubmed?term=Gao E%5BAuthor%5D&cauthor=true&cauthor_uid=16492926) (2006) Joint effects of the CYP1A1 MspI, ERalpha PvuII, and ERalpha XbaI polymorphisms on the risk of breast cancer: results from a population-based case-control study in Shanghai, China. [Cancer Epidemiol Biomarkers Prev](http://www.ncbi.nlm.nih.gov/pubmed/?term=Joint+effects+of+the+CYP1A1+MspI%2C+ERalpha+PvuII%2C+and+ERalpha+XbaI+polymorphisms+on+the+risk+of+breast+cancer%3A+results+from+a+population-based+case-control+study+in+Shanghai%2C+China) 15:342-347.
26. [Shimada N](http://www.ncbi.nlm.nih.gov/pubmed?term=Shimada N%5BAuthor%5D&cauthor=true&cauthor_uid=19229255), [Iwasaki M](http://www.ncbi.nlm.nih.gov/pubmed?term=Iwasaki M%5BAuthor%5D&cauthor=true&cauthor_uid=19229255), [Kasuga Y](http://www.ncbi.nlm.nih.gov/pubmed?term=Kasuga Y%5BAuthor%5D&cauthor=true&cauthor_uid=19229255), [Yokoyama S](http://www.ncbi.nlm.nih.gov/pubmed?term=Yokoyama S%5BAuthor%5D&cauthor=true&cauthor_uid=19229255), [Onuma H](http://www.ncbi.nlm.nih.gov/pubmed?term=Onuma H%5BAuthor%5D&cauthor=true&cauthor_uid=19229255), et al. (2009) Genetic polymorphisms in estrogen metabolism and breast cancer risk in case-control studies in Japanese, Japanese Brazilians and non-Japanese Brazilians. [J Hum Genet](http://www.ncbi.nlm.nih.gov/pubmed/19229255) 54:209-215.
27. [Shin A](http://www.ncbi.nlm.nih.gov/pubmed?term=Shin A%5BAuthor%5D&cauthor=true&cauthor_uid=17603290), [Kang D](http://www.ncbi.nlm.nih.gov/pubmed?term=Kang D%5BAuthor%5D&cauthor=true&cauthor_uid=17603290), [Choi JY](http://www.ncbi.nlm.nih.gov/pubmed?term=Choi JY%5BAuthor%5D&cauthor=true&cauthor_uid=17603290), [Lee KM](http://www.ncbi.nlm.nih.gov/pubmed?term=Lee KM%5BAuthor%5D&cauthor=true&cauthor_uid=17603290), [Park SK](http://www.ncbi.nlm.nih.gov/pubmed?term=Park SK%5BAuthor%5D&cauthor=true&cauthor_uid=17603290), et al. (2007) Cytochrome P450 1A1 (CYP1A1) polymorphisms and breast cancer risk in Korean women. [Exp Mol Med](http://www.ncbi.nlm.nih.gov/pubmed/17603290) 39:361-366.
28. [Sillanpää P](http://www.ncbi.nlm.nih.gov/pubmed?term=Sillanpää P%5BAuthor%5D&cauthor=true&cauthor_uid=17063266), [Heikinheimo L](http://www.ncbi.nlm.nih.gov/pubmed?term=Heikinheimo L%5BAuthor%5D&cauthor=true&cauthor_uid=17063266), [Kataja V](http://www.ncbi.nlm.nih.gov/pubmed?term=Kataja V%5BAuthor%5D&cauthor=true&cauthor_uid=17063266), [Eskelinen M](http://www.ncbi.nlm.nih.gov/pubmed?term=Eskelinen M%5BAuthor%5D&cauthor=true&cauthor_uid=17063266), [Kosma VM](http://www.ncbi.nlm.nih.gov/pubmed?term=Kosma VM%5BAuthor%5D&cauthor=true&cauthor_uid=17063266), et al. (2007) CYP1A1 and CYP1B1 genetic polymorphisms, smoking and breast cancer risk in a Finnish Caucasian population. [Breast Cancer Res Treat](http://www.ncbi.nlm.nih.gov/pubmed/?term=CYP1A1+and+CYP1B1+genetic+polymorphisms%2C+smoking+and+breast+cancer+risk+in+a+Finnish+Caucasian+population) 104:287-297.
29. [Singh N](http://www.ncbi.nlm.nih.gov/pubmed?term=Singh N%5BAuthor%5D&cauthor=true&cauthor_uid=18351133), [Mitra AK](http://www.ncbi.nlm.nih.gov/pubmed?term=Mitra AK%5BAuthor%5D&cauthor=true&cauthor_uid=18351133), [Garg VK](http://www.ncbi.nlm.nih.gov/pubmed?term=Garg VK%5BAuthor%5D&cauthor=true&cauthor_uid=18351133), [Agarwal A](http://www.ncbi.nlm.nih.gov/pubmed?term=Agarwal A%5BAuthor%5D&cauthor=true&cauthor_uid=18351133), [Sharma M](http://www.ncbi.nlm.nih.gov/pubmed?term=Sharma M%5BAuthor%5D&cauthor=true&cauthor_uid=18351133), et al. (2007) Association of CYP1A1 polymorphisms with breast cancer in North Indian women. [Oncol Res](http://www.ncbi.nlm.nih.gov/pubmed/18351133) 16:587-597.
30. [Singh V](http://www.ncbi.nlm.nih.gov/pubmed?term=Singh V%5BAuthor%5D&cauthor=true&cauthor_uid=16807674), [Rastogi N](http://www.ncbi.nlm.nih.gov/pubmed?term=Rastogi N%5BAuthor%5D&cauthor=true&cauthor_uid=16807674), [Sinha A](http://www.ncbi.nlm.nih.gov/pubmed?term=Sinha A%5BAuthor%5D&cauthor=true&cauthor_uid=16807674), [Kumar A](http://www.ncbi.nlm.nih.gov/pubmed?term=Kumar A%5BAuthor%5D&cauthor=true&cauthor_uid=16807674), [Mathur N](http://www.ncbi.nlm.nih.gov/pubmed?term=Mathur N%5BAuthor%5D&cauthor=true&cauthor_uid=16807674), et al. (2007) A study on the association of cytochrome-P450 1A1 polymorphism and breast cancer risk in north Indian women. [Breast Cancer Res Treat](http://www.ncbi.nlm.nih.gov/pubmed/16807674) 101:73-81.
31. [Syamala VS](http://www.ncbi.nlm.nih.gov/pubmed?term=Syamala VS%5BAuthor%5D&cauthor=true&cauthor_uid=19863350), [Syamala V](http://www.ncbi.nlm.nih.gov/pubmed?term=Syamala V%5BAuthor%5D&cauthor=true&cauthor_uid=19863350), [Sheeja VR](http://www.ncbi.nlm.nih.gov/pubmed?term=Sheeja VR%5BAuthor%5D&cauthor=true&cauthor_uid=19863350), [Kuttan R](http://www.ncbi.nlm.nih.gov/pubmed?term=Kuttan R%5BAuthor%5D&cauthor=true&cauthor_uid=19863350), [Balakrishnan R](http://www.ncbi.nlm.nih.gov/pubmed?term=Balakrishnan R%5BAuthor%5D&cauthor=true&cauthor_uid=19863350), et al. (2010) Possible risk modification by polymorphisms of estrogen metabolizing genes in familial breast cancer susceptibility in an Indian population. [Cancer Invest](http://www.ncbi.nlm.nih.gov/pubmed/?term=Possible+risk+modification+by+polymorphisms+of+estrogen+metabolizing+genes+in+familial+breast+cancer+susceptibility+in+an+Indian+population) 28:304-311.
32. [Taioli E](http://www.ncbi.nlm.nih.gov/pubmed?term=Taioli E%5BAuthor%5D&cauthor=true&cauthor_uid=10337002), [Bradlow HL](http://www.ncbi.nlm.nih.gov/pubmed?term=Bradlow HL%5BAuthor%5D&cauthor=true&cauthor_uid=10337002), [Garbers SV](http://www.ncbi.nlm.nih.gov/pubmed?term=Garbers SV%5BAuthor%5D&cauthor=true&cauthor_uid=10337002), [Sepkovic DW](http://www.ncbi.nlm.nih.gov/pubmed?term=Sepkovic DW%5BAuthor%5D&cauthor=true&cauthor_uid=10337002), [Osborne MP](http://www.ncbi.nlm.nih.gov/pubmed?term=Osborne MP%5BAuthor%5D&cauthor=true&cauthor_uid=10337002), et al. (1999) Role of estradiol metabolism and CYP1A1 polymorphisms in breast cancer risk. [Cancer Detect Prev](http://www.ncbi.nlm.nih.gov/pubmed/10337002) 23:232-237.
33. [Taioli E](http://www.ncbi.nlm.nih.gov/pubmed?term=Taioli E%5BAuthor%5D&cauthor=true&cauthor_uid=7641189), [Trachman J](http://www.ncbi.nlm.nih.gov/pubmed?term=Trachman J%5BAuthor%5D&cauthor=true&cauthor_uid=7641189), [Chen X](http://www.ncbi.nlm.nih.gov/pubmed?term=Chen X%5BAuthor%5D&cauthor=true&cauthor_uid=7641189), [Toniolo P](http://www.ncbi.nlm.nih.gov/pubmed?term=Toniolo P%5BAuthor%5D&cauthor=true&cauthor_uid=7641189), [Garte SJ](http://www.ncbi.nlm.nih.gov/pubmed?term=Garte SJ%5BAuthor%5D&cauthor=true&cauthor_uid=7641189) (1995) A CYP1A1 restriction fragment length polymorphism is associated with breast cancer in African-American women. [Cancer Res](http://www.ncbi.nlm.nih.gov/pubmed/?term=A+CYP1A1+restriction+fragment+length+polymorphism+is+associated+with+breast+cancer+in+African-American+women) 55:3757-3758.
34. [Wang Q](http://www.ncbi.nlm.nih.gov/pubmed?term=Wang Q%5BAuthor%5D&cauthor=true&cauthor_uid=21438753), [Li H](http://www.ncbi.nlm.nih.gov/pubmed?term=Li H%5BAuthor%5D&cauthor=true&cauthor_uid=21438753), [Tao P](http://www.ncbi.nlm.nih.gov/pubmed?term=Tao P%5BAuthor%5D&cauthor=true&cauthor_uid=21438753), [Wang YP](http://www.ncbi.nlm.nih.gov/pubmed?term=Wang YP%5BAuthor%5D&cauthor=true&cauthor_uid=21438753), [Yuan P](http://www.ncbi.nlm.nih.gov/pubmed?term=Yuan P%5BAuthor%5D&cauthor=true&cauthor_uid=21438753), et al. (2011) Soy isoflavones, CYP1A1, CYP1B1, and COMT polymorphisms, and breast cancer: a case-control study in southwestern China. [DNA Cell Biol](http://www.ncbi.nlm.nih.gov/pubmed/?term=Soy+isoflavones%2C+CYP1A1%2C+CYP1B1%2C+and+COMT+polymorphisms%2C+and+breast+cancer%3A+a+case-control+study+in+southwestern+China) 30:585-595.
35. [Fontana X](http://www.ncbi.nlm.nih.gov/pubmed?term=Fontana X%5BAuthor%5D&cauthor=true&cauthor_uid=9726005), [Peyrottes I](http://www.ncbi.nlm.nih.gov/pubmed?term=Peyrottes I%5BAuthor%5D&cauthor=true&cauthor_uid=9726005), [Rossi C](http://www.ncbi.nlm.nih.gov/pubmed?term=Rossi C%5BAuthor%5D&cauthor=true&cauthor_uid=9726005), [Leblanc-Talent P](http://www.ncbi.nlm.nih.gov/pubmed?term=Leblanc-Talent P%5BAuthor%5D&cauthor=true&cauthor_uid=9726005), [Ettore F](http://www.ncbi.nlm.nih.gov/pubmed?term=Ettore F%5BAuthor%5D&cauthor=true&cauthor_uid=9726005), et al. (1998) Study of the frequencies of CYP1A1 gene polymorphisms and glutathione S-transferase mu1 gene in primary breast cancers: an update with an additional 114 cases. [Mutat Res](http://www.ncbi.nlm.nih.gov/pubmed?term=study of the frequencies of cyp1a1 gene polymorphisms and glutathione s-transferase mu1 gene in primary breast cancers an update with an additional 114 causes&cmd=correctspelling) 403:45-53.
36. [Gutman G](http://www.ncbi.nlm.nih.gov/pubmed?term=Gutman G%5BAuthor%5D&cauthor=true&cauthor_uid=20009880), [Morad T](http://www.ncbi.nlm.nih.gov/pubmed?term=Morad T%5BAuthor%5D&cauthor=true&cauthor_uid=20009880), [Peleg B](http://www.ncbi.nlm.nih.gov/pubmed?term=Peleg B%5BAuthor%5D&cauthor=true&cauthor_uid=20009880), [Peretz C](http://www.ncbi.nlm.nih.gov/pubmed?term=Peretz C%5BAuthor%5D&cauthor=true&cauthor_uid=20009880), [Bar-Am A](http://www.ncbi.nlm.nih.gov/pubmed?term=Bar-Am A%5BAuthor%5D&cauthor=true&cauthor_uid=20009880), et al. (2009) CYP1A1 and CYP2D6 gene polymorphisms in Israeli Jewish women with cervical cancer. [Int J Gynecol Cancer](http://www.ncbi.nlm.nih.gov/pubmed/?term=CYP1A1+and+CYP2D6+gene+polymorphisms+in+Israeli+Jewish+women+with+cervical+cancer) 19:1300-1302.
37. [Joseph T](http://www.ncbi.nlm.nih.gov/pubmed?term=Joseph T%5BAuthor%5D&cauthor=true&cauthor_uid=16360200), [Chacko P](http://www.ncbi.nlm.nih.gov/pubmed?term=Chacko P%5BAuthor%5D&cauthor=true&cauthor_uid=16360200), [Wesley R](http://www.ncbi.nlm.nih.gov/pubmed?term=Wesley R%5BAuthor%5D&cauthor=true&cauthor_uid=16360200), [Jayaprakash PG](http://www.ncbi.nlm.nih.gov/pubmed?term=Jayaprakash PG%5BAuthor%5D&cauthor=true&cauthor_uid=16360200), [James FV](http://www.ncbi.nlm.nih.gov/pubmed?term=James FV%5BAuthor%5D&cauthor=true&cauthor_uid=16360200), et al. (2006) Germline genetic polymorphisms of CYP1A1, GSTM1 and GSTT1 genes in Indian cervical cancer: associations with tumor progression, age and human papillomavirus infection. [Gynecol Oncol](http://www.ncbi.nlm.nih.gov/pubmed/?term=Germline+genetic+polymorphisms+of+CYP1A1%2C+GSTM1+and+GSTT1+genes+in+Indian+cervical+cancer%3A+associations+with+tumor+progression%2C+age+and+human+papillomavirus+infection) 101:411-417.
38. [Juárez-Cedillo T](http://www.ncbi.nlm.nih.gov/pubmed?term=Juárez-Cedillo T%5BAuthor%5D&cauthor=true&cauthor_uid=17512722), [Vallejo M](http://www.ncbi.nlm.nih.gov/pubmed?term=Vallejo M%5BAuthor%5D&cauthor=true&cauthor_uid=17512722), [Fragoso JM](http://www.ncbi.nlm.nih.gov/pubmed?term=Fragoso JM%5BAuthor%5D&cauthor=true&cauthor_uid=17512722), [Hernández-Hernández DM](http://www.ncbi.nlm.nih.gov/pubmed?term=Hernández-Hernández DM%5BAuthor%5D&cauthor=true&cauthor_uid=17512722), [Rodríguez-Pérez JM](http://www.ncbi.nlm.nih.gov/pubmed?term=Rodríguez-Pérez JM%5BAuthor%5D&cauthor=true&cauthor_uid=17512722), et al. (2007) The risk of developing cervical cancer in Mexican women is associated to CYP1A1 MspI polymorphism. [Eur J Cancer](http://www.ncbi.nlm.nih.gov/pubmed/?term=The+risk+of+developing+cervical+cancer+in+Mexican+women+is+associated+to+CYP1A1+MspI+polymorphism) 43:1590-1595.
39. [Nishino K](http://www.ncbi.nlm.nih.gov/pubmed?term=Nishino K%5BAuthor%5D&cauthor=true&cauthor_uid=19012698), [Sekine M](http://www.ncbi.nlm.nih.gov/pubmed?term=Sekine M%5BAuthor%5D&cauthor=true&cauthor_uid=19012698), [Kodama S](http://www.ncbi.nlm.nih.gov/pubmed?term=Kodama S%5BAuthor%5D&cauthor=true&cauthor_uid=19012698), [Sudo N](http://www.ncbi.nlm.nih.gov/pubmed?term=Sudo N%5BAuthor%5D&cauthor=true&cauthor_uid=19012698), [Aoki Y](http://www.ncbi.nlm.nih.gov/pubmed?term=Aoki Y%5BAuthor%5D&cauthor=true&cauthor_uid=19012698), et al. (2008) Cigarette smoking and glutathione S-transferase M1 polymorphism associated with risk for uterine cervical cancer. [J Obstet Gynaecol Res](http://www.ncbi.nlm.nih.gov/pubmed/19012698) 34:994-1001.
40. [Sugawara T](http://www.ncbi.nlm.nih.gov/pubmed?term=Sugawara T%5BAuthor%5D&cauthor=true&cauthor_uid=14675315), [Nomura E](http://www.ncbi.nlm.nih.gov/pubmed?term=Nomura E%5BAuthor%5D&cauthor=true&cauthor_uid=14675315), [Sagawa T](http://www.ncbi.nlm.nih.gov/pubmed?term=Sagawa T%5BAuthor%5D&cauthor=true&cauthor_uid=14675315), [Sakuragi N](http://www.ncbi.nlm.nih.gov/pubmed?term=Sakuragi N%5BAuthor%5D&cauthor=true&cauthor_uid=14675315), [Fujimoto S](http://www.ncbi.nlm.nih.gov/pubmed?term=Fujimoto S%5BAuthor%5D&cauthor=true&cauthor_uid=14675315) (2003) CYP1A1 polymorphism and risk of gynecological malignancy in Japan. [Int J Gynecol Cancer](http://www.ncbi.nlm.nih.gov/pubmed/14675315) 13:785-790.
41. [Cleary SP](http://www.ncbi.nlm.nih.gov/pubmed?term=Cleary SP%5BAuthor%5D&cauthor=true&cauthor_uid=20937634), [Cotterchio M](http://www.ncbi.nlm.nih.gov/pubmed?term=Cotterchio M%5BAuthor%5D&cauthor=true&cauthor_uid=20937634), [Shi E](http://www.ncbi.nlm.nih.gov/pubmed?term=Shi E%5BAuthor%5D&cauthor=true&cauthor_uid=20937634), [Gallinger S](http://www.ncbi.nlm.nih.gov/pubmed?term=Gallinger S%5BAuthor%5D&cauthor=true&cauthor_uid=20937634), [Harper P](http://www.ncbi.nlm.nih.gov/pubmed?term=Harper P%5BAuthor%5D&cauthor=true&cauthor_uid=20937634) (2010) Cigarette smoking, genetic variants in carcinogen-metabolizing enzymes, and colorectal cancer risk. [Am J Epidemiol](http://www.ncbi.nlm.nih.gov/pubmed/?term=Cigarette+smoking%2C+genetic+variants+in+carcinogen-metabolizing+enzymes%2C+and+colorectal+cancer+risk) 172:1000-1014.
42. [Hou L](http://www.ncbi.nlm.nih.gov/pubmed?term=Hou L%5BAuthor%5D&cauthor=true&cauthor_uid=15731166), [Chatterjee N](http://www.ncbi.nlm.nih.gov/pubmed?term=Chatterjee N%5BAuthor%5D&cauthor=true&cauthor_uid=15731166), [Huang WY](http://www.ncbi.nlm.nih.gov/pubmed?term=Huang WY%5BAuthor%5D&cauthor=true&cauthor_uid=15731166), [Baccarelli A](http://www.ncbi.nlm.nih.gov/pubmed?term=Baccarelli A%5BAuthor%5D&cauthor=true&cauthor_uid=15731166), [Yadavalli S](http://www.ncbi.nlm.nih.gov/pubmed?term=Yadavalli S%5BAuthor%5D&cauthor=true&cauthor_uid=15731166), et al. (2005) CYP1A1 Val462 and NQO1 Ser187 polymorphisms, cigarette use, and risk for colorectal adenoma. [Carcinogenesis](http://www.ncbi.nlm.nih.gov/pubmed/?term=CYP1A1+Val462+and+NQO1+Ser187+polymorphisms%2C+cigarette+use%2C+and+risk+for+colorectal+adenoma) 26:1122-1128.
43. [Inoue H](http://www.ncbi.nlm.nih.gov/pubmed?term=Inoue H%5BAuthor%5D&cauthor=true&cauthor_uid=10919645), [Kiyohara C](http://www.ncbi.nlm.nih.gov/pubmed?term=Kiyohara C%5BAuthor%5D&cauthor=true&cauthor_uid=10919645), [Marugame T](http://www.ncbi.nlm.nih.gov/pubmed?term=Marugame T%5BAuthor%5D&cauthor=true&cauthor_uid=10919645), [Shinomiya S](http://www.ncbi.nlm.nih.gov/pubmed?term=Shinomiya S%5BAuthor%5D&cauthor=true&cauthor_uid=10919645), [Tsuji E](http://www.ncbi.nlm.nih.gov/pubmed?term=Tsuji E%5BAuthor%5D&cauthor=true&cauthor_uid=10919645), et al. (2000) Cigarette smoking, CYP1A1 MspI and GSTM1 genotypes, and colorectal adenomas. [Cancer Res](http://www.ncbi.nlm.nih.gov/pubmed/?term=Cigarette+smoking%2C+CYP1A1+MspI+and+GSTM1+genotypes%2C+and+colorectal+adenomas.) 60:3749-3752.
44. [Kobayashi M](http://www.ncbi.nlm.nih.gov/pubmed?term=Kobayashi M%5BAuthor%5D&cauthor=true&cauthor_uid=19452301), [Otani T](http://www.ncbi.nlm.nih.gov/pubmed?term=Otani T%5BAuthor%5D&cauthor=true&cauthor_uid=19452301), [Iwasaki M](http://www.ncbi.nlm.nih.gov/pubmed?term=Iwasaki M%5BAuthor%5D&cauthor=true&cauthor_uid=19452301), [Natsukawa S](http://www.ncbi.nlm.nih.gov/pubmed?term=Natsukawa S%5BAuthor%5D&cauthor=true&cauthor_uid=19452301), [Shaura K](http://www.ncbi.nlm.nih.gov/pubmed?term=Shaura K%5BAuthor%5D&cauthor=true&cauthor_uid=19452301), et al. (2009) Association between dietary heterocyclic amine levels, genetic polymorphisms of NAT2, CYP1A1, and CYP1A2 and risk of colorectal cancer: a hospital-based case-control study in Japan. [Scand J Gastroenterol](http://www.ncbi.nlm.nih.gov/pubmed/?term=Association+between+dietary+heterocyclic+amine+levels%2C+genetic+polymorphisms+of+NAT2%2C+CYP1A1%2C+and+CYP1A2+and+risk+of+colorectal+cancer%3A+a+hospital-based+case-control+study+in+Japan) 44:952-959.
45. [Little J](http://www.ncbi.nlm.nih.gov/pubmed?term=Little J%5BAuthor%5D&cauthor=true&cauthor_uid=16823842), [Sharp L](http://www.ncbi.nlm.nih.gov/pubmed?term=Sharp L%5BAuthor%5D&cauthor=true&cauthor_uid=16823842), [Masson LF](http://www.ncbi.nlm.nih.gov/pubmed?term=Masson LF%5BAuthor%5D&cauthor=true&cauthor_uid=16823842), [Brockton NT](http://www.ncbi.nlm.nih.gov/pubmed?term=Brockton NT%5BAuthor%5D&cauthor=true&cauthor_uid=16823842), [Cotton SC](http://www.ncbi.nlm.nih.gov/pubmed?term=Cotton SC%5BAuthor%5D&cauthor=true&cauthor_uid=16823842), et al. (2006) Colorectal cancer and genetic polymorphisms of CYP1A1, GSTM1 and GSTT1: a case-control study in the Grampian region of Scotland. [Int J Cancer](http://www.ncbi.nlm.nih.gov/pubmed/?term=Colorectal+cancer+and+genetic+polymorphisms+of+CYP1A1%2C+GSTM1+and+GSTT1%3A+a+case-control+study+in+the+Grampian+region+of+Scotland) 119:2155-2164.
46. [Nisa H](http://www.ncbi.nlm.nih.gov/pubmed?term=Nisa H%5BAuthor%5D&cauthor=true&cauthor_uid=20534171), [Kono S](http://www.ncbi.nlm.nih.gov/pubmed?term=Kono S%5BAuthor%5D&cauthor=true&cauthor_uid=20534171), [Yin G](http://www.ncbi.nlm.nih.gov/pubmed?term=Yin G%5BAuthor%5D&cauthor=true&cauthor_uid=20534171), [Toyomura K](http://www.ncbi.nlm.nih.gov/pubmed?term=Toyomura K%5BAuthor%5D&cauthor=true&cauthor_uid=20534171), [Nagano J](http://www.ncbi.nlm.nih.gov/pubmed?term=Nagano J%5BAuthor%5D&cauthor=true&cauthor_uid=20534171), et al. (2010) Cigarette smoking, genetic polymorphisms and colorectal cancer risk: the Fukuoka Colorectal Cancer Study. [BMC Cancer](http://www.ncbi.nlm.nih.gov/pubmed/20534171) 10:274.
47. [Pereira Serafim PV](http://www.ncbi.nlm.nih.gov/pubmed?term=Pereira Serafim PV%5BAuthor%5D&cauthor=true&cauthor_uid=18409146), [Cotrim Guerreiro da Silva ID](http://www.ncbi.nlm.nih.gov/pubmed?term=Cotrim Guerreiro da Silva ID%5BAuthor%5D&cauthor=true&cauthor_uid=18409146), [Manoukias Forones N](http://www.ncbi.nlm.nih.gov/pubmed?term=Manoukias Forones N%5BAuthor%5D&cauthor=true&cauthor_uid=18409146) (2008) Relationship between genetic polymorphism of CYP1A1 at codon 462 (Ile462Val) in colorectal cancer. [Int J Biol Markers](http://www.ncbi.nlm.nih.gov/pubmed/?term=Relationship+between+genetic+polymorphism+of+CYP1A1+at+codon+462+(Ile462Val)+in+colorectal+cancer) 23:18-23.
48. [Rudolph A](http://www.ncbi.nlm.nih.gov/pubmed?term=Rudolph A%5BAuthor%5D&cauthor=true&cauthor_uid=21490239), [Sainz J](http://www.ncbi.nlm.nih.gov/pubmed?term=Sainz J%5BAuthor%5D&cauthor=true&cauthor_uid=21490239), [Hein R](http://www.ncbi.nlm.nih.gov/pubmed?term=Hein R%5BAuthor%5D&cauthor=true&cauthor_uid=21490239), [Hoffmeister M](http://www.ncbi.nlm.nih.gov/pubmed?term=Hoffmeister M%5BAuthor%5D&cauthor=true&cauthor_uid=21490239), [Frank B](http://www.ncbi.nlm.nih.gov/pubmed?term=Frank B%5BAuthor%5D&cauthor=true&cauthor_uid=21490239), et al. (2011) Modification of menopausal hormone therapy-associated colorectal cancer risk by polymorphisms in sex steroid signaling, metabolism and transport related genes. [Endocr Relat Cancer](http://www.ncbi.nlm.nih.gov/pubmed/?term=Modification+of+menopausal+hormone+therapy-associated+colorectal+cancer+risk+by+polymorphisms+in+sex+steroid+signaling%2C+metabolism+and+transport+related+genes) 18:371-384.
49. [Sivaraman L](http://www.ncbi.nlm.nih.gov/pubmed?term=Sivaraman L%5BAuthor%5D&cauthor=true&cauthor_uid=7913406), [Leatham MP](http://www.ncbi.nlm.nih.gov/pubmed?term=Leatham MP%5BAuthor%5D&cauthor=true&cauthor_uid=7913406), [Yee J](http://www.ncbi.nlm.nih.gov/pubmed?term=Yee J%5BAuthor%5D&cauthor=true&cauthor_uid=7913406), [Wilkens LR](http://www.ncbi.nlm.nih.gov/pubmed?term=Wilkens LR%5BAuthor%5D&cauthor=true&cauthor_uid=7913406), [Lau AF](http://www.ncbi.nlm.nih.gov/pubmed?term=Lau AF%5BAuthor%5D&cauthor=true&cauthor_uid=7913406), et al. (1994) CYP1A1 genetic polymorphisms and in situ colorectal cancer. [Cancer Res](http://www.ncbi.nlm.nih.gov/pubmed/?term=CYP1A1+genetic+polymorphisms+and+in+situ+colorectal+cancer) 54:3692-3695.
50. [Slattery ML](http://www.ncbi.nlm.nih.gov/pubmed?term=Slattery ML%5BAuthor%5D&cauthor=true&cauthor_uid=15496536), [Samowtiz W](http://www.ncbi.nlm.nih.gov/pubmed?term=Samowtiz W%5BAuthor%5D&cauthor=true&cauthor_uid=15496536), [Ma K](http://www.ncbi.nlm.nih.gov/pubmed?term=Ma K%5BAuthor%5D&cauthor=true&cauthor_uid=15496536), [Murtaugh M](http://www.ncbi.nlm.nih.gov/pubmed?term=Murtaugh M%5BAuthor%5D&cauthor=true&cauthor_uid=15496536), [Sweeney C](http://www.ncbi.nlm.nih.gov/pubmed?term=Sweeney C%5BAuthor%5D&cauthor=true&cauthor_uid=15496536), et al. (2004) CYP1A1, cigarette smoking, and colon and rectal cancer. [Am J Epidemiol](http://www.ncbi.nlm.nih.gov/pubmed/15496536) 160:842-852.
51. [Ye Z](http://www.ncbi.nlm.nih.gov/pubmed?term=Ye Z%5BAuthor%5D&cauthor=true&cauthor_uid=12210502), [Parry JM](http://www.ncbi.nlm.nih.gov/pubmed?term=Parry JM%5BAuthor%5D&cauthor=true&cauthor_uid=12210502) (2002) Genetic polymorphisms in the cytochrome P450 1A1, glutathione S-transferase M1 and T1, and susceptibility to colon cancer. [Teratog Carcinog Mutagen](http://www.ncbi.nlm.nih.gov/pubmed/?term=Genetic+polymorphisms+in+the+cytochrome+P450+1A1%2C+glutathione+S-transferase+M1+and+T1%2C+and+susceptibility+to+colon+cancer) 22:385-392.
52. [Yeh CC](http://www.ncbi.nlm.nih.gov/pubmed?term=Yeh CC%5BAuthor%5D&cauthor=true&cauthor_uid=17191090), [Sung FC](http://www.ncbi.nlm.nih.gov/pubmed?term=Sung FC%5BAuthor%5D&cauthor=true&cauthor_uid=17191090), [Tang R](http://www.ncbi.nlm.nih.gov/pubmed?term=Tang R%5BAuthor%5D&cauthor=true&cauthor_uid=17191090), [Chang-Chieh CR](http://www.ncbi.nlm.nih.gov/pubmed?term=Chang-Chieh CR%5BAuthor%5D&cauthor=true&cauthor_uid=17191090), [Hsieh LL](http://www.ncbi.nlm.nih.gov/pubmed?term=Hsieh LL%5BAuthor%5D&cauthor=true&cauthor_uid=17191090) (2007) Association between polymorphisms of biotransformation and DNA-repair genes and risk of colorectal cancer in Taiwan. [J Biomed Sci](http://www.ncbi.nlm.nih.gov/pubmed/?term=Association+between+polymorphisms+of+biotransformation+and+DNA-repair+genes+and+risk+of+colorectal+cancer+in+Taiwan) 14:183-193.
53. [Yoshida K](http://www.ncbi.nlm.nih.gov/pubmed?term=Yoshida K%5BAuthor%5D&cauthor=true&cauthor_uid=18159984), [Osawa K](http://www.ncbi.nlm.nih.gov/pubmed?term=Osawa K%5BAuthor%5D&cauthor=true&cauthor_uid=18159984), [Kasahara M](http://www.ncbi.nlm.nih.gov/pubmed?term=Kasahara M%5BAuthor%5D&cauthor=true&cauthor_uid=18159984), [Miyaishi A](http://www.ncbi.nlm.nih.gov/pubmed?term=Miyaishi A%5BAuthor%5D&cauthor=true&cauthor_uid=18159984), [Nakanishi K](http://www.ncbi.nlm.nih.gov/pubmed?term=Nakanishi K%5BAuthor%5D&cauthor=true&cauthor_uid=18159984), et al. (2007) Association of CYP1A1, CYP1A2, GSTM1 and NAT2 gene polymorphisms with colorectal cancer and smoking. [Asian Pac J Cancer Prev](http://www.ncbi.nlm.nih.gov/pubmed/18159984) 8:438-444.
54. [Ashton KA](http://www.ncbi.nlm.nih.gov/pubmed?term=Ashton KA%5BAuthor%5D&cauthor=true&cauthor_uid=20381444), [Proietto A](http://www.ncbi.nlm.nih.gov/pubmed?term=Proietto A%5BAuthor%5D&cauthor=true&cauthor_uid=20381444), [Otton G](http://www.ncbi.nlm.nih.gov/pubmed?term=Otton G%5BAuthor%5D&cauthor=true&cauthor_uid=20381444), [Symonds I](http://www.ncbi.nlm.nih.gov/pubmed?term=Symonds I%5BAuthor%5D&cauthor=true&cauthor_uid=20381444), [McEvoy M](http://www.ncbi.nlm.nih.gov/pubmed?term=McEvoy M%5BAuthor%5D&cauthor=true&cauthor_uid=20381444), et al. (2010)Polymorphisms in genes of the steroid hormone biosynthesis and metabolism pathways and endometrial cancer risk. [Cancer Epidemiol](http://www.ncbi.nlm.nih.gov/pubmed/20381444) 34:328-337.
55. [Doherty JA](http://www.ncbi.nlm.nih.gov/pubmed?term=Doherty JA%5BAuthor%5D&cauthor=true&cauthor_uid=15734958), [Weiss NS](http://www.ncbi.nlm.nih.gov/pubmed?term=Weiss NS%5BAuthor%5D&cauthor=true&cauthor_uid=15734958), [Freeman RJ](http://www.ncbi.nlm.nih.gov/pubmed?term=Freeman RJ%5BAuthor%5D&cauthor=true&cauthor_uid=15734958), [Dightman DA](http://www.ncbi.nlm.nih.gov/pubmed?term=Dightman DA%5BAuthor%5D&cauthor=true&cauthor_uid=15734958), [Thornton PJ](http://www.ncbi.nlm.nih.gov/pubmed?term=Thornton PJ%5BAuthor%5D&cauthor=true&cauthor_uid=15734958), et al. (2005) Genetic factors in catechol estrogen metabolism in relation to the risk of endometrial cancer. [Cancer Epidemiol Biomarkers Prev](http://www.ncbi.nlm.nih.gov/pubmed/15734958) 14:357-366.
56. [Esteller M](http://www.ncbi.nlm.nih.gov/pubmed?term=Esteller M%5BAuthor%5D&cauthor=true&cauthor_uid=9155064), [García A](http://www.ncbi.nlm.nih.gov/pubmed?term=García A%5BAuthor%5D&cauthor=true&cauthor_uid=9155064), [Martínez-Palones JM](http://www.ncbi.nlm.nih.gov/pubmed?term=Martínez-Palones JM%5BAuthor%5D&cauthor=true&cauthor_uid=9155064), [Xercavins J](http://www.ncbi.nlm.nih.gov/pubmed?term=Xercavins J%5BAuthor%5D&cauthor=true&cauthor_uid=9155064), [Reventós J](http://www.ncbi.nlm.nih.gov/pubmed?term=Reventós J%5BAuthor%5D&cauthor=true&cauthor_uid=9155064) (1997) Susceptibility to endometrial cancer: influence of allelism at p53, glutathione S-transferase (GSTM1 and GSTT1) and cytochrome P-450 (CYP1A1) loci. [Br J Cancer](http://www.ncbi.nlm.nih.gov/pubmed/?term=Susceptibility+to+endometrial+cancer%3A+influence+of+allelism+at+p53%2C+glutathione+S-transferase+(GSTM1+and+GSTT1)+and+cytochrome+P-450+(CYP1A1)+loci) 75:1385-1388.
57. [Gulyaeva LF](http://www.ncbi.nlm.nih.gov/pubmed?term=Gulyaeva LF%5BAuthor%5D&cauthor=true&cauthor_uid=18497059), [Mikhailova ON](http://www.ncbi.nlm.nih.gov/pubmed?term=Mikhailova ON%5BAuthor%5D&cauthor=true&cauthor_uid=18497059), [PustyInyak VO](http://www.ncbi.nlm.nih.gov/pubmed?term=PustyInyak VO%5BAuthor%5D&cauthor=true&cauthor_uid=18497059), [Kim IV 4th](http://www.ncbi.nlm.nih.gov/pubmed?term=Kim IV 4th%5BAuthor%5D&cauthor=true&cauthor_uid=18497059), [Gerasimov AV](http://www.ncbi.nlm.nih.gov/pubmed?term=Gerasimov AV%5BAuthor%5D&cauthor=true&cauthor_uid=18497059), et al. (2008) Comparative analysis of SNP in estrogen-metabolizing enzymes for ovarian, endometrial, and breast cancers in Novosibirsk, Russia. [Adv Exp Med Biol](http://www.ncbi.nlm.nih.gov/pubmed/?term=Comparative+analysis+of+SNP+in+estrogen-metabolizing+enzymes+for+ovarian%2C+endometrial%2C+and+breast+cancers+in+Novosibirsk%2C+Russia) 617:359-366.
58. [Hirata H](http://www.ncbi.nlm.nih.gov/pubmed?term=Hirata H%5BAuthor%5D&cauthor=true&cauthor_uid=18318428), [Hinoda Y](http://www.ncbi.nlm.nih.gov/pubmed?term=Hinoda Y%5BAuthor%5D&cauthor=true&cauthor_uid=18318428), [Okayama N](http://www.ncbi.nlm.nih.gov/pubmed?term=Okayama N%5BAuthor%5D&cauthor=true&cauthor_uid=18318428), [Suehiro Y](http://www.ncbi.nlm.nih.gov/pubmed?term=Suehiro Y%5BAuthor%5D&cauthor=true&cauthor_uid=18318428), [Kawamoto K](http://www.ncbi.nlm.nih.gov/pubmed?term=Kawamoto K%5BAuthor%5D&cauthor=true&cauthor_uid=18318428), et al. (2008) CYP1A1, SULT1A1, and SULT1E1 polymorphisms are risk factors for endometrial cancer susceptibility. [Cancer](http://www.ncbi.nlm.nih.gov/pubmed/?term=CYP1A1%2C+SULT1A1%2C+and+SULT1E1+polymorphisms+are+risk+factors+for+endometrial+cancer+susceptibility) 112:1964-1973.
59. [McGrath M](http://www.ncbi.nlm.nih.gov/pubmed?term=McGrath M%5BAuthor%5D&cauthor=true&cauthor_uid=17717632), [Hankinson SE](http://www.ncbi.nlm.nih.gov/pubmed?term=Hankinson SE%5BAuthor%5D&cauthor=true&cauthor_uid=17717632), [De Vivo I](http://www.ncbi.nlm.nih.gov/pubmed?term=De Vivo I%5BAuthor%5D&cauthor=true&cauthor_uid=17717632) (2007) Cytochrome P450 1A1, cigarette smoking, and risk of endometrial cancer (United States). [Cancer Causes Control](http://www.ncbi.nlm.nih.gov/pubmed/?term=Cytochrome+P450+1A1%2C+cigarette+smoking%2C+and+risk+of+endometrial+cancer+(United+States).) 18:1123-1130.
60. [Hori H](http://www.ncbi.nlm.nih.gov/pubmed?term=Hori H%5BAuthor%5D&cauthor=true&cauthor_uid=9451664), [Kawano T](http://www.ncbi.nlm.nih.gov/pubmed?term=Kawano T%5BAuthor%5D&cauthor=true&cauthor_uid=9451664), [Endo M](http://www.ncbi.nlm.nih.gov/pubmed?term=Endo M%5BAuthor%5D&cauthor=true&cauthor_uid=9451664), [Yuasa Y](http://www.ncbi.nlm.nih.gov/pubmed?term=Yuasa Y%5BAuthor%5D&cauthor=true&cauthor_uid=9451664) (1997) Genetic polymorphisms of tobacco- and alcohol-related metabolizing enzymes and human esophageal squamous cell carcinoma susceptibility. [J Clin Gastroenterol](http://www.ncbi.nlm.nih.gov/pubmed?term=genetic polymorphisms of tobacco and alcohol-related metabolizing enzymes and human esophageal squamous cell carcinoma susceptibility&cmd=correctspelling) 25:568-575.
61. [Jain M](http://www.ncbi.nlm.nih.gov/pubmed?term=Jain M%5BAuthor%5D&cauthor=true&cauthor_uid=18074679), [Kumar S](http://www.ncbi.nlm.nih.gov/pubmed?term=Kumar S%5BAuthor%5D&cauthor=true&cauthor_uid=18074679), [Ghoshal UC](http://www.ncbi.nlm.nih.gov/pubmed?term=Ghoshal UC%5BAuthor%5D&cauthor=true&cauthor_uid=18074679), [Mittal B](http://www.ncbi.nlm.nih.gov/pubmed?term=Mittal B%5BAuthor%5D&cauthor=true&cauthor_uid=18074679) (2007) CYP1A1 Msp1 T/C polymorphism in esophageal cancer: no association and risk modulation. [Oncol Res](http://www.ncbi.nlm.nih.gov/pubmed/?term=CYP1A1+Msp1+T%2FC+polymorphism+in+esophageal+cancer%3A+no+association+and+risk+modulation) 16:437-443.
62. [Malik MA](http://www.ncbi.nlm.nih.gov/pubmed?term=Malik MA%5BAuthor%5D&cauthor=true&cauthor_uid=20661821), [Upadhyay R](http://www.ncbi.nlm.nih.gov/pubmed?term=Upadhyay R%5BAuthor%5D&cauthor=true&cauthor_uid=20661821), [Mittal RD](http://www.ncbi.nlm.nih.gov/pubmed?term=Mittal RD%5BAuthor%5D&cauthor=true&cauthor_uid=20661821), [Zargar SA](http://www.ncbi.nlm.nih.gov/pubmed?term=Zargar SA%5BAuthor%5D&cauthor=true&cauthor_uid=20661821), [Mittal B](http://www.ncbi.nlm.nih.gov/pubmed?term=Mittal B%5BAuthor%5D&cauthor=true&cauthor_uid=20661821) (2010) Association of xenobiotic metabolizing enzymes genetic polymorphisms with esophageal cancer in Kashmir Valley and influence of environmental factors. [Nutr Cancer](http://www.ncbi.nlm.nih.gov/pubmed/?term=Association+of+xenobiotic+metabolizing+enzymes+genetic+polymorphisms+with+esophageal+cancer+in+Kashmir+Valley+and+influence+of+environmental+factors) 62:734-742.
63. [Morita S](http://www.ncbi.nlm.nih.gov/pubmed?term=Morita S%5BAuthor%5D&cauthor=true&cauthor_uid=9139841), [Yano M](http://www.ncbi.nlm.nih.gov/pubmed?term=Yano M%5BAuthor%5D&cauthor=true&cauthor_uid=9139841), [Shiozaki H](http://www.ncbi.nlm.nih.gov/pubmed?term=Shiozaki H%5BAuthor%5D&cauthor=true&cauthor_uid=9139841), [Tsujinaka T](http://www.ncbi.nlm.nih.gov/pubmed?term=Tsujinaka T%5BAuthor%5D&cauthor=true&cauthor_uid=9139841), [Ebisui C](http://www.ncbi.nlm.nih.gov/pubmed?term=Ebisui C%5BAuthor%5D&cauthor=true&cauthor_uid=9139841), et al. (1997) CYP1A1, CYP2E1 and GSTM1 polymorphisms are not associated with susceptibility to squamous-cell carcinoma of the esophagus. [Int J Cancer](http://www.ncbi.nlm.nih.gov/pubmed/9139841) 71:192-195.
64. [Nimura Y](http://www.ncbi.nlm.nih.gov/pubmed?term=Nimura Y%5BAuthor%5D&cauthor=true&cauthor_uid=9307183), [Yokoyama S](http://www.ncbi.nlm.nih.gov/pubmed?term=Yokoyama S%5BAuthor%5D&cauthor=true&cauthor_uid=9307183), [Fujimori M](http://www.ncbi.nlm.nih.gov/pubmed?term=Fujimori M%5BAuthor%5D&cauthor=true&cauthor_uid=9307183), [Aoki T](http://www.ncbi.nlm.nih.gov/pubmed?term=Aoki T%5BAuthor%5D&cauthor=true&cauthor_uid=9307183), [Adachi W](http://www.ncbi.nlm.nih.gov/pubmed?term=Adachi W%5BAuthor%5D&cauthor=true&cauthor_uid=9307183), et al. (1997) Genotyping of the CYP1A1 and GSTM1 genes in esophageal carcinoma patients with special reference to smoking. [Cancer](http://www.ncbi.nlm.nih.gov/pubmed/?term=Genotyping+of+the+CYP1A1+and+GSTM1+genes+in+esophageal+carcinoma+patients+with+special+reference+to+smoking) 80:852-857.
65. [van Lieshout EM](http://www.ncbi.nlm.nih.gov/pubmed?term=van Lieshout EM%5BAuthor%5D&cauthor=true&cauthor_uid=9973204), [Roelofs HM](http://www.ncbi.nlm.nih.gov/pubmed?term=Roelofs HM%5BAuthor%5D&cauthor=true&cauthor_uid=9973204), [Dekker S](http://www.ncbi.nlm.nih.gov/pubmed?term=Dekker S%5BAuthor%5D&cauthor=true&cauthor_uid=9973204), [Mulder CJ](http://www.ncbi.nlm.nih.gov/pubmed?term=Mulder CJ%5BAuthor%5D&cauthor=true&cauthor_uid=9973204), [Wobbes T](http://www.ncbi.nlm.nih.gov/pubmed?term=Wobbes T%5BAuthor%5D&cauthor=true&cauthor_uid=9973204), et al. (1999) Polymorphic expression of the glutathione S-transferase P1 gene and its susceptibility to Barrett's esophagus and esophageal carcinoma. [Cancer Res](http://www.ncbi.nlm.nih.gov/pubmed/?term=Polymorphic+expression+of+the+glutathione+S-transferase+P1+gene+and+its+susceptibility+to+Barrett's+esophagus+and+esophageal+carcinoma) 59:586-589.
66. [Wang AH](http://www.ncbi.nlm.nih.gov/pubmed?term=Wang AH%5BAuthor%5D&cauthor=true&cauthor_uid=15052670), [Sun CS](http://www.ncbi.nlm.nih.gov/pubmed?term=Sun CS%5BAuthor%5D&cauthor=true&cauthor_uid=15052670), [Li LS](http://www.ncbi.nlm.nih.gov/pubmed?term=Li LS%5BAuthor%5D&cauthor=true&cauthor_uid=15052670), [Huang JY](http://www.ncbi.nlm.nih.gov/pubmed?term=Huang JY%5BAuthor%5D&cauthor=true&cauthor_uid=15052670), [Chen QS](http://www.ncbi.nlm.nih.gov/pubmed?term=Chen QS%5BAuthor%5D&cauthor=true&cauthor_uid=15052670), et al. (2004) Genetic susceptibility and environmental factors of esophageal cancer in Xi'an. [World J Gastroenterol](http://www.ncbi.nlm.nih.gov/pubmed/15052670) 10:940-944.
67. [Wang AH](http://www.ncbi.nlm.nih.gov/pubmed?term=Wang AH%5BAuthor%5D&cauthor=true&cauthor_uid=11833070), [Sun CS](http://www.ncbi.nlm.nih.gov/pubmed?term=Sun CS%5BAuthor%5D&cauthor=true&cauthor_uid=11833070), [Li LS](http://www.ncbi.nlm.nih.gov/pubmed?term=Li LS%5BAuthor%5D&cauthor=true&cauthor_uid=11833070), [Huang JY](http://www.ncbi.nlm.nih.gov/pubmed?term=Huang JY%5BAuthor%5D&cauthor=true&cauthor_uid=11833070), [Chen QS](http://www.ncbi.nlm.nih.gov/pubmed?term=Chen QS%5BAuthor%5D&cauthor=true&cauthor_uid=11833070) (2002) Relationship of tobacco smoking CYP1A1 GSTM1 gene polymorphism and esophageal cancer in Xi'an. [World J Gastroenterol](http://www.ncbi.nlm.nih.gov/pubmed/?term=Relationship+of+tobacco+smoking+CYP1A1+GSTM1+gene+polymorphism+and+esophageal+cancer+in+Xi'an) 8:49-53.
68. [Wang D](http://www.ncbi.nlm.nih.gov/pubmed?term=Wang D%5BAuthor%5D&cauthor=true&cauthor_uid=22088806), [Su M](http://www.ncbi.nlm.nih.gov/pubmed?term=Su M%5BAuthor%5D&cauthor=true&cauthor_uid=22088806), [Tian D](http://www.ncbi.nlm.nih.gov/pubmed?term=Tian D%5BAuthor%5D&cauthor=true&cauthor_uid=22088806), [Liang S](http://www.ncbi.nlm.nih.gov/pubmed?term=Liang S%5BAuthor%5D&cauthor=true&cauthor_uid=22088806), [Zhang J](http://www.ncbi.nlm.nih.gov/pubmed?term=Zhang J%5BAuthor%5D&cauthor=true&cauthor_uid=22088806) (2012) Associations between CYP1A1 and CYP2E1 polymorphisms and susceptibility to esophageal cancer in Chaoshan and Taihang areas of China. [Cancer Epidemiol](http://www.ncbi.nlm.nih.gov/pubmed/?term=Associations+between+CYP1A1+and+CYP2E1+polymorphisms+and+susceptibility+to+esophageal+cancer+in+Chaoshan+and+Taihang+areas+of+China) 36:276-282.
69. [Wang LD](http://www.ncbi.nlm.nih.gov/pubmed?term=Wang LD%5BAuthor%5D&cauthor=true&cauthor_uid=12854128), [Zheng S](http://www.ncbi.nlm.nih.gov/pubmed?term=Zheng S%5BAuthor%5D&cauthor=true&cauthor_uid=12854128), [Liu B](http://www.ncbi.nlm.nih.gov/pubmed?term=Liu B%5BAuthor%5D&cauthor=true&cauthor_uid=12854128), [Zhou JX](http://www.ncbi.nlm.nih.gov/pubmed?term=Zhou JX%5BAuthor%5D&cauthor=true&cauthor_uid=12854128), [Li YJ](http://www.ncbi.nlm.nih.gov/pubmed?term=Li YJ%5BAuthor%5D&cauthor=true&cauthor_uid=12854128), et al. (2003) CYP1A1, GSTs and mEH polymorphisms and susceptibility to esophageal carcinoma: study of population from a high- incidence area in north China. [World J Gastroenterol](http://www.ncbi.nlm.nih.gov/pubmed?term=cyp1a1, gsts and meh polymorphisms and susceptibility to esophageal carcinoma study of population from a high incidence area in north china&cmd=correctspelling) 9:1394-1397.
70. [Wu MT](http://www.ncbi.nlm.nih.gov/pubmed?term=Wu MT%5BAuthor%5D&cauthor=true&cauthor_uid=12189551), [Lee JM](http://www.ncbi.nlm.nih.gov/pubmed?term=Lee JM%5BAuthor%5D&cauthor=true&cauthor_uid=12189551), [Wu DC](http://www.ncbi.nlm.nih.gov/pubmed?term=Wu DC%5BAuthor%5D&cauthor=true&cauthor_uid=12189551), [Ho CK](http://www.ncbi.nlm.nih.gov/pubmed?term=Ho CK%5BAuthor%5D&cauthor=true&cauthor_uid=12189551), [Wang YT](http://www.ncbi.nlm.nih.gov/pubmed?term=Wang YT%5BAuthor%5D&cauthor=true&cauthor_uid=12189551), et al. Genetic polymorphisms of cytochrome P4501A1 and oesophageal squamous-cell carcinoma in Taiwan. [Br J Cancer](http://www.ncbi.nlm.nih.gov/pubmed/?term=Genetic+polymorphisms+of+cytochrome+P4501A1+and+oesophageal+squamous-cell+carcinoma+in+Taiwan) 87:529-532.
71. [Kimura A](http://www.ncbi.nlm.nih.gov/pubmed?term=Kimura A%5BAuthor%5D&cauthor=true&cauthor_uid=18990008), [Tsuchiya Y](http://www.ncbi.nlm.nih.gov/pubmed?term=Tsuchiya Y%5BAuthor%5D&cauthor=true&cauthor_uid=18990008), [Lang I](http://www.ncbi.nlm.nih.gov/pubmed?term=Lang I%5BAuthor%5D&cauthor=true&cauthor_uid=18990008), [Zoltan S](http://www.ncbi.nlm.nih.gov/pubmed?term=Zoltan S%5BAuthor%5D&cauthor=true&cauthor_uid=18990008), [Nakadaira H](http://www.ncbi.nlm.nih.gov/pubmed?term=Nakadaira H%5BAuthor%5D&cauthor=true&cauthor_uid=18990008), et al. (2008) Effect of genetic predisposition on the risk of gallbladder cancer in Hungary. [Asian Pac J Cancer Prev](http://www.ncbi.nlm.nih.gov/pubmed/?term=Effect+of+genetic+predisposition+on+the+risk+of+gallbladder+cancer+in+Hungary) 9:391-396.
72. [Pandey SN](http://www.ncbi.nlm.nih.gov/pubmed?term=Pandey SN%5BAuthor%5D&cauthor=true&cauthor_uid=18287863), [Choudhuri G](http://www.ncbi.nlm.nih.gov/pubmed?term=Choudhuri G%5BAuthor%5D&cauthor=true&cauthor_uid=18287863), [Mittal B](http://www.ncbi.nlm.nih.gov/pubmed?term=Mittal B%5BAuthor%5D&cauthor=true&cauthor_uid=18287863) (2008) Association of CYP1A1 Msp1 polymorphism with tobacco-related risk of gallbladder cancer in a north Indian population. [Eur J Cancer Prev](http://www.ncbi.nlm.nih.gov/pubmed/?term=Association+of+CYP1A1+Msp1+polymorphism+with+tobacco-related+risk+of+gallbladder+cancer+in+a+north+Indian+population) 17:77-81.
73. [Tsuchiya Y](http://www.ncbi.nlm.nih.gov/pubmed?term=Tsuchiya Y%5BAuthor%5D&cauthor=true&cauthor_uid=17531965), [Kiyohara C](http://www.ncbi.nlm.nih.gov/pubmed?term=Kiyohara C%5BAuthor%5D&cauthor=true&cauthor_uid=17531965), [Sato T](http://www.ncbi.nlm.nih.gov/pubmed?term=Sato T%5BAuthor%5D&cauthor=true&cauthor_uid=17531965), [Nakamura K](http://www.ncbi.nlm.nih.gov/pubmed?term=Nakamura K%5BAuthor%5D&cauthor=true&cauthor_uid=17531965), [Kimura A](http://www.ncbi.nlm.nih.gov/pubmed?term=Kimura A%5BAuthor%5D&cauthor=true&cauthor_uid=17531965), et al. (2007) Polymorphisms of cytochrome P450 1A1, glutathione S-transferase class mu, and tumour protein p53 genes and the risk of developing gallbladder cancer in Japanese. [Clin Biochem](http://www.ncbi.nlm.nih.gov/pubmed/?term=Polymorphisms+of+cytochrome+P450+1A1%2C+glutathione+S-transferase+class+mu%2C+and+tumour+protein+p53+genes+and+the+risk+of+developing+gallbladder+cancer+in+Japanese) 40:881-886.
74. [Agudo A](http://www.ncbi.nlm.nih.gov/pubmed?term=Agudo A%5BAuthor%5D&cauthor=true&cauthor_uid=17164366), [Sala N](http://www.ncbi.nlm.nih.gov/pubmed?term=Sala N%5BAuthor%5D&cauthor=true&cauthor_uid=17164366), [Pera G](http://www.ncbi.nlm.nih.gov/pubmed?term=Pera G%5BAuthor%5D&cauthor=true&cauthor_uid=17164366), [Capellá G](http://www.ncbi.nlm.nih.gov/pubmed?term=Capellá G%5BAuthor%5D&cauthor=true&cauthor_uid=17164366), [Berenguer A](http://www.ncbi.nlm.nih.gov/pubmed?term=Berenguer A%5BAuthor%5D&cauthor=true&cauthor_uid=17164366), et al. (2006) Polymorphisms in metabolic genes related to tobacco smoke and the risk of gastric cancer in the European prospective investigation into cancer and nutrition. [Cancer Epidemiol Biomarkers Prev](http://www.ncbi.nlm.nih.gov/pubmed/?term=Polymorphisms+in+metabolic+genes+related+to+tobacco+smoke+and+the+risk+of+gastric+cancer+in+the+European+prospective+investigation+into+cancer+and+nutrition) 15:2427-2434.
75. [Kobayashi M](http://www.ncbi.nlm.nih.gov/pubmed?term=Kobayashi M%5BAuthor%5D&cauthor=true&cauthor_uid=20047124), [Otani T](http://www.ncbi.nlm.nih.gov/pubmed?term=Otani T%5BAuthor%5D&cauthor=true&cauthor_uid=20047124), [Iwasaki M](http://www.ncbi.nlm.nih.gov/pubmed?term=Iwasaki M%5BAuthor%5D&cauthor=true&cauthor_uid=20047124), [Natsukawa S](http://www.ncbi.nlm.nih.gov/pubmed?term=Natsukawa S%5BAuthor%5D&cauthor=true&cauthor_uid=20047124), [Shaura K](http://www.ncbi.nlm.nih.gov/pubmed?term=Shaura K%5BAuthor%5D&cauthor=true&cauthor_uid=20047124), et al. (2009) Association between dietary heterocyclic amine levels, genetic polymorphisms of NAT2, CYP1A1, and CYP1A2 and risk of stomach cancer: a hospital-based case-control study in Japan. [Gastric Cancer](http://www.ncbi.nlm.nih.gov/pubmed/?term=Association+between+dietary+heterocyclic+amine+levels%2C+genetic+polymorphisms+of+NAT2%2C+CYP1A1%2C+and+CYP1A2+and+risk+of+stomach+cancer%3A+a+hospital-based+case-control+study+in+Japan) 12:198-205.
76. [Li H](http://www.ncbi.nlm.nih.gov/pubmed?term=Li H%5BAuthor%5D&cauthor=true&cauthor_uid=16270381), [Chen XL](http://www.ncbi.nlm.nih.gov/pubmed?term=Chen XL%5BAuthor%5D&cauthor=true&cauthor_uid=16270381), [Li HQ](http://www.ncbi.nlm.nih.gov/pubmed?term=Li HQ%5BAuthor%5D&cauthor=true&cauthor_uid=16270381) (2005) Polymorphism of CYPIA1 and GSTM1 genes associated with susceptibility of gastric cancer in Shandong Province of China. [World J Gastroenterol](http://www.ncbi.nlm.nih.gov/pubmed/?term=Polymorphism+of+CYPIA1+and+GSTM1+genes+associated+with+susceptibility+of+gastric+cancer+in+Shandong+Province+of+China) 11:5757-5762.
77. [Luo YP](http://www.ncbi.nlm.nih.gov/pubmed?term=Luo YP%5BAuthor%5D&cauthor=true&cauthor_uid=20878561), [Chen HC](http://www.ncbi.nlm.nih.gov/pubmed?term=Chen HC%5BAuthor%5D&cauthor=true&cauthor_uid=20878561), [Khan MA](http://www.ncbi.nlm.nih.gov/pubmed?term=Khan MA%5BAuthor%5D&cauthor=true&cauthor_uid=20878561), [Chen FZ](http://www.ncbi.nlm.nih.gov/pubmed?term=Chen FZ%5BAuthor%5D&cauthor=true&cauthor_uid=20878561), [Wan XX](http://www.ncbi.nlm.nih.gov/pubmed?term=Wan XX%5BAuthor%5D&cauthor=true&cauthor_uid=20878561), et al. (2011) Genetic polymorphisms of metabolic enzymes-CYP1A1, CYP2D6, GSTM1, and GSTT1, and gastric carcinoma susceptibility. [Tumour Biol](http://www.ncbi.nlm.nih.gov/pubmed/?term=Genetic+polymorphisms+of+metabolic+enzymes-CYP1A1%2C+CYP2D6%2C+GSTM1%2C+and+GSTT1%2C+and+gastric+carcinoma+susceptibility) 32:215-222.
78. [Ma JX](http://www.ncbi.nlm.nih.gov/pubmed?term=Ma JX%5BAuthor%5D&cauthor=true&cauthor_uid=16337337), [Zhang KL](http://www.ncbi.nlm.nih.gov/pubmed?term=Zhang KL%5BAuthor%5D&cauthor=true&cauthor_uid=16337337), [Liu X](http://www.ncbi.nlm.nih.gov/pubmed?term=Liu X%5BAuthor%5D&cauthor=true&cauthor_uid=16337337), [Ma YL](http://www.ncbi.nlm.nih.gov/pubmed?term=Ma YL%5BAuthor%5D&cauthor=true&cauthor_uid=16337337), [Pei LN](http://www.ncbi.nlm.nih.gov/pubmed?term=Pei LN%5BAuthor%5D&cauthor=true&cauthor_uid=16337337), et al. (2006) Concurrent expression of aryl hydrocarbon receptor and CYP1A1 but not CYP1A1 MspI polymorphism is correlated with gastric cancers raised in Dalian, China. [Cancer Lett](http://www.ncbi.nlm.nih.gov/pubmed/16337337) 240:253-260.
79. [Malik MA](http://www.ncbi.nlm.nih.gov/pubmed?term=Malik MA%5BAuthor%5D&cauthor=true&cauthor_uid=19521675), [Upadhyay R](http://www.ncbi.nlm.nih.gov/pubmed?term=Upadhyay R%5BAuthor%5D&cauthor=true&cauthor_uid=19521675), [Mittal RD](http://www.ncbi.nlm.nih.gov/pubmed?term=Mittal RD%5BAuthor%5D&cauthor=true&cauthor_uid=19521675), [Zargar SA](http://www.ncbi.nlm.nih.gov/pubmed?term=Zargar SA%5BAuthor%5D&cauthor=true&cauthor_uid=19521675), [Modi DR](http://www.ncbi.nlm.nih.gov/pubmed?term=Modi DR%5BAuthor%5D&cauthor=true&cauthor_uid=19521675) et al. (2009) Role of xenobiotic-metabolizing enzyme gene polymorphisms and interactions with environmental factors in susceptibility to gastric cancer in Kashmir Valley. [J Gastrointest Cancer](http://www.ncbi.nlm.nih.gov/pubmed/?term=Role+of+xenobiotic-metabolizing+enzyme+gene+polymorphisms+and+interactions+with+environmental+factors+in+susceptibility+to+gastric+cancer+in+Kashmir+Valley) 40:26-32.
80. [Shen J](http://www.ncbi.nlm.nih.gov/pubmed?term=Shen J%5BAuthor%5D&cauthor=true&cauthor_uid=16273625), [Wang RT](http://www.ncbi.nlm.nih.gov/pubmed?term=Wang RT%5BAuthor%5D&cauthor=true&cauthor_uid=16273625), [Xu YC](http://www.ncbi.nlm.nih.gov/pubmed?term=Xu YC%5BAuthor%5D&cauthor=true&cauthor_uid=16273625), [Wang LW](http://www.ncbi.nlm.nih.gov/pubmed?term=Wang LW%5BAuthor%5D&cauthor=true&cauthor_uid=16273625), [Wang XR](http://www.ncbi.nlm.nih.gov/pubmed?term=Wang XR%5BAuthor%5D&cauthor=true&cauthor_uid=16273625) (2005) Interaction models of CYP1A1, GSTM1 polymorphisms and tobacco smoking in intestinal gastric cancer. [World J Gastroenterol](http://www.ncbi.nlm.nih.gov/pubmed/?term=Interaction+models+of+CYP1A1%2C+GSTM1+polymorphisms+and+tobacco+smoking+in+intestinal+gastric+cancer) 11:6056-6060.
81. [Cheng YJ](http://www.ncbi.nlm.nih.gov/pubmed?term=Cheng YJ%5BAuthor%5D&cauthor=true&cauthor_uid=12582034), [Chien YC](http://www.ncbi.nlm.nih.gov/pubmed?term=Chien YC%5BAuthor%5D&cauthor=true&cauthor_uid=12582034), [Hildesheim A](http://www.ncbi.nlm.nih.gov/pubmed?term=Hildesheim A%5BAuthor%5D&cauthor=true&cauthor_uid=12582034), [Hsu MM](http://www.ncbi.nlm.nih.gov/pubmed?term=Hsu MM%5BAuthor%5D&cauthor=true&cauthor_uid=12582034), [Chen IH](http://www.ncbi.nlm.nih.gov/pubmed?term=Chen IH%5BAuthor%5D&cauthor=true&cauthor_uid=12582034) et al. (2003) No association between genetic polymorphisms of CYP1A1, GSTM1, GSTT1, GSTP1, NAT2, and nasopharyngeal carcinoma in Taiwan. [Cancer Epidemiol Biomarkers Prev](http://www.ncbi.nlm.nih.gov/pubmed/?term=No+association+between+genetic+polymorphisms+of+CYP1A1%2C+GSTM1%2C+GSTT1%2C+GSTP1%2C+NAT2%2C+and+nasopharyngeal+carcinoma+in+Taiwan) 12:179-180.
82. [Gronau S](http://www.ncbi.nlm.nih.gov/pubmed?term=Gronau S%5BAuthor%5D&cauthor=true&cauthor_uid=12748560), [Koenig-Greger D](http://www.ncbi.nlm.nih.gov/pubmed?term=Koenig-Greger D%5BAuthor%5D&cauthor=true&cauthor_uid=12748560), [Jerg M](http://www.ncbi.nlm.nih.gov/pubmed?term=Jerg M%5BAuthor%5D&cauthor=true&cauthor_uid=12748560), [Riechelmann H](http://www.ncbi.nlm.nih.gov/pubmed?term=Riechelmann H%5BAuthor%5D&cauthor=true&cauthor_uid=12748560) (2003) Gene polymorphisms in detoxification enzymes as susceptibility factor for head and neck cancer? [Otolaryngol Head Neck Surg](http://www.ncbi.nlm.nih.gov/pubmed/12748560) 128:674-680.
83. [Harth V](http://www.ncbi.nlm.nih.gov/pubmed?term=Harth V%5BAuthor%5D&cauthor=true&cauthor_uid=18569591), [Schafer M](http://www.ncbi.nlm.nih.gov/pubmed?term=Schafer M%5BAuthor%5D&cauthor=true&cauthor_uid=18569591), [Abel J](http://www.ncbi.nlm.nih.gov/pubmed?term=Abel J%5BAuthor%5D&cauthor=true&cauthor_uid=18569591), [Maintz L](http://www.ncbi.nlm.nih.gov/pubmed?term=Maintz L%5BAuthor%5D&cauthor=true&cauthor_uid=18569591), [Neuhaus T](http://www.ncbi.nlm.nih.gov/pubmed?term=Neuhaus T%5BAuthor%5D&cauthor=true&cauthor_uid=18569591) et al. (2008) Head and neck squamous-cell cancer and its association with polymorphic enzymes of xenobiotic metabolism and repair. [J Toxicol Environ Health A](http://www.ncbi.nlm.nih.gov/pubmed/?term=Head+and+neck+squamous-cell+cancer+and+its+association+with+polymorphic+enzymes+of+xenobiotic+metabolism+and+repair) 71:887-897.
84. [Ko Y](http://www.ncbi.nlm.nih.gov/pubmed?term=Ko Y%5BAuthor%5D&cauthor=true&cauthor_uid=11389067), [Abel J](http://www.ncbi.nlm.nih.gov/pubmed?term=Abel J%5BAuthor%5D&cauthor=true&cauthor_uid=11389067), [Harth V](http://www.ncbi.nlm.nih.gov/pubmed?term=Harth V%5BAuthor%5D&cauthor=true&cauthor_uid=11389067), [Bröde P](http://www.ncbi.nlm.nih.gov/pubmed?term=Bröde P%5BAuthor%5D&cauthor=true&cauthor_uid=11389067), [Antony C](http://www.ncbi.nlm.nih.gov/pubmed?term=Antony C%5BAuthor%5D&cauthor=true&cauthor_uid=11389067) et al. (2001) Association of CYP1B1 codon 432 mutant allele in head and neck squamous cell cancer is reflected by somatic mutations of p53 in tumor tissue. [Cancer Res](http://www.ncbi.nlm.nih.gov/pubmed/?term=Association+of+CYP1B1+codon+432+mutant+allele+in+head+and+neck+squamous+cell+cancer+is+reflected+by+somatic+mutations+of+p53+in+tumor+tissue) 61:4398-4404.
85. [Matthias C](http://www.ncbi.nlm.nih.gov/pubmed?term=Matthias C%5BAuthor%5D&cauthor=true&cauthor_uid=10022746), [Bockmühl U](http://www.ncbi.nlm.nih.gov/pubmed?term=Bockmühl U%5BAuthor%5D&cauthor=true&cauthor_uid=10022746), [Jahnke V](http://www.ncbi.nlm.nih.gov/pubmed?term=Jahnke V%5BAuthor%5D&cauthor=true&cauthor_uid=10022746), [Jones PW](http://www.ncbi.nlm.nih.gov/pubmed?term=Jones PW%5BAuthor%5D&cauthor=true&cauthor_uid=10022746), [Hayes JD](http://www.ncbi.nlm.nih.gov/pubmed?term=Hayes JD%5BAuthor%5D&cauthor=true&cauthor_uid=10022746) et al. (1998) Polymorphism in cytochrome P450 CYP2D6, CYP1A1, CYP2E1 and glutathione S-transferase, GSTM1, GSTM3, GSTT1 and susceptibility to tobacco-related cancers: studies in upper aerodigestive tract cancers. [Pharmacogenetics](http://www.ncbi.nlm.nih.gov/pubmed/?term=Polymorphism+in+cytochrome+P450+CYP2D6%2C+CYP1A1%2C+CYP2E1+and+glutathione+S-transferase%2C+GSTM1%2C+GSTM3%2C+GSTT1+and+susceptibility+to+tobacco-related+cancers%3A+studies+in+upper+aerodigestive+tract+cancers) 8:91-100.
86. [McWilliams JE](http://www.ncbi.nlm.nih.gov/pubmed?term=McWilliams JE%5BAuthor%5D&cauthor=true&cauthor_uid=10941163), [Evans AJ](http://www.ncbi.nlm.nih.gov/pubmed?term=Evans AJ%5BAuthor%5D&cauthor=true&cauthor_uid=10941163), [Beer TM](http://www.ncbi.nlm.nih.gov/pubmed?term=Beer TM%5BAuthor%5D&cauthor=true&cauthor_uid=10941163), [Andersen PE](http://www.ncbi.nlm.nih.gov/pubmed?term=Andersen PE%5BAuthor%5D&cauthor=true&cauthor_uid=10941163), [Cohen JI](http://www.ncbi.nlm.nih.gov/pubmed?term=Cohen JI%5BAuthor%5D&cauthor=true&cauthor_uid=10941163) et al. (2000) Genetic polymorphisms in head and neck cancer risk. [Head Neck](http://www.ncbi.nlm.nih.gov/pubmed/10941163) 22:609-617.
87. [Morita S](http://www.ncbi.nlm.nih.gov/pubmed?term=Morita S%5BAuthor%5D&cauthor=true&cauthor_uid=10048967), [Yano M](http://www.ncbi.nlm.nih.gov/pubmed?term=Yano M%5BAuthor%5D&cauthor=true&cauthor_uid=10048967), [Tsujinaka T](http://www.ncbi.nlm.nih.gov/pubmed?term=Tsujinaka T%5BAuthor%5D&cauthor=true&cauthor_uid=10048967), [Akiyama Y](http://www.ncbi.nlm.nih.gov/pubmed?term=Akiyama Y%5BAuthor%5D&cauthor=true&cauthor_uid=10048967), [Taniguchi M](http://www.ncbi.nlm.nih.gov/pubmed?term=Taniguchi M%5BAuthor%5D&cauthor=true&cauthor_uid=10048967) et al. (1999) Genetic polymorphisms of drug-metabolizing enzymes and susceptibility to head-and-neck squamous-cell carcinoma. [Int J Cancer](http://www.ncbi.nlm.nih.gov/pubmed/10048967) 80:685-688.
88. [Olshan AF](http://www.ncbi.nlm.nih.gov/pubmed?term=Olshan AF%5BAuthor%5D&cauthor=true&cauthor_uid=10698480), [Weissler MC](http://www.ncbi.nlm.nih.gov/pubmed?term=Weissler MC%5BAuthor%5D&cauthor=true&cauthor_uid=10698480), [Watson MA](http://www.ncbi.nlm.nih.gov/pubmed?term=Watson MA%5BAuthor%5D&cauthor=true&cauthor_uid=10698480), [Bell DA](http://www.ncbi.nlm.nih.gov/pubmed?term=Bell DA%5BAuthor%5D&cauthor=true&cauthor_uid=10698480) (2000) GSTM1, GSTT1, GSTP1, CYP1A1, and NAT1 polymorphisms, tobacco use, and the risk of head and neck cancer. [Cancer Epidemiol Biomarkers Prev](http://www.ncbi.nlm.nih.gov/pubmed/?term=GSTM1%2C+GSTT1%2C+GSTP1%2C+CYP1A1%2C+and+NAT1+polymorphisms%2C+tobacco+use%2C+and+the+risk+of+head+and+neck+cancer) 9:185-191.
89. [Sam SS](http://www.ncbi.nlm.nih.gov/pubmed?term=Sam SS%5BAuthor%5D&cauthor=true&cauthor_uid=18767181), [Thomas V](http://www.ncbi.nlm.nih.gov/pubmed?term=Thomas V%5BAuthor%5D&cauthor=true&cauthor_uid=18767181), [Reddy SK](http://www.ncbi.nlm.nih.gov/pubmed?term=Reddy SK%5BAuthor%5D&cauthor=true&cauthor_uid=18767181), [Surianarayanan G](http://www.ncbi.nlm.nih.gov/pubmed?term=Surianarayanan G%5BAuthor%5D&cauthor=true&cauthor_uid=18767181), [Chandrasekaran A](http://www.ncbi.nlm.nih.gov/pubmed?term=Chandrasekaran A%5BAuthor%5D&cauthor=true&cauthor_uid=18767181) (2008) CYP1A1 polymorphisms and the risk of upper aerodigestive tract cancers in an Indian population. [Head Neck](http://www.ncbi.nlm.nih.gov/pubmed/18767181) 30:1566-1574.
90. [Sharma R](http://www.ncbi.nlm.nih.gov/pubmed?term=Sharma R%5BAuthor%5D&cauthor=true&cauthor_uid=20491576), [Ahuja M](http://www.ncbi.nlm.nih.gov/pubmed?term=Ahuja M%5BAuthor%5D&cauthor=true&cauthor_uid=20491576), [Panda NK](http://www.ncbi.nlm.nih.gov/pubmed?term=Panda NK%5BAuthor%5D&cauthor=true&cauthor_uid=20491576), [Khullar M](http://www.ncbi.nlm.nih.gov/pubmed?term=Khullar M%5BAuthor%5D&cauthor=true&cauthor_uid=20491576) (2010) Combined effect of smoking and polymorphisms in tobacco carcinogen-metabolizing enzymes CYP1A1 and GSTM1 on the head and neck cancer risk in North Indians. [DNA Cell Biol](http://www.ncbi.nlm.nih.gov/pubmed/?term=Combined+effect+of+smoking+and+polymorphisms+in+tobacco+carcinogen-metabolizing+enzymes+CYP1A1+and+GSTM1+on+the+head+and+neck+cancer+risk+in+North+Indians) 29:441-448.
91. [Singh AP](http://www.ncbi.nlm.nih.gov/pubmed?term=Singh AP%5BAuthor%5D&cauthor=true&cauthor_uid=19639480), [Shah PP](http://www.ncbi.nlm.nih.gov/pubmed?term=Shah PP%5BAuthor%5D&cauthor=true&cauthor_uid=19639480), [Ruwali M](http://www.ncbi.nlm.nih.gov/pubmed?term=Ruwali M%5BAuthor%5D&cauthor=true&cauthor_uid=19639480), [Mathur N](http://www.ncbi.nlm.nih.gov/pubmed?term=Mathur N%5BAuthor%5D&cauthor=true&cauthor_uid=19639480), [Pant MC](http://www.ncbi.nlm.nih.gov/pubmed?term=Pant MC%5BAuthor%5D&cauthor=true&cauthor_uid=19639480) et al. (2009) Polymorphism in cytochrome P4501A1 is significantly associated with head and neck cancer risk. [Cancer Invest](http://www.ncbi.nlm.nih.gov/pubmed/19639480) 27:869-876.
92. [Tai J](http://www.ncbi.nlm.nih.gov/pubmed?term=Tai J%5BAuthor%5D&cauthor=true&cauthor_uid=19963139), [Yang M](http://www.ncbi.nlm.nih.gov/pubmed?term=Yang M%5BAuthor%5D&cauthor=true&cauthor_uid=19963139), [Ni X](http://www.ncbi.nlm.nih.gov/pubmed?term=Ni X%5BAuthor%5D&cauthor=true&cauthor_uid=19963139), [Yu D](http://www.ncbi.nlm.nih.gov/pubmed?term=Yu D%5BAuthor%5D&cauthor=true&cauthor_uid=19963139), [Fang J](http://www.ncbi.nlm.nih.gov/pubmed?term=Fang J%5BAuthor%5D&cauthor=true&cauthor_uid=19963139) et al. (2010) Genetic polymorphisms in cytochrome P450 genes are associated with an increased risk of squamous cell carcinoma of the larynx and hypo.pharynx in a Chinese population. [Cancer Genet Cytogenet](http://www.ncbi.nlm.nih.gov/pubmed/?term=Genetic+polymorphisms+in+cytochrome+P450+genes+are+associated+with+an+increased+risk+of+squamous+cell+carcinoma+of+the+larynx+and+hypopharynx+in+a+Chinese+population) 196:76-82.
93. [Varzim G](http://www.ncbi.nlm.nih.gov/pubmed?term=Varzim G%5BAuthor%5D&cauthor=true&cauthor_uid=14639127), [Monteiro E](http://www.ncbi.nlm.nih.gov/pubmed?term=Monteiro E%5BAuthor%5D&cauthor=true&cauthor_uid=14639127), [Silva RA](http://www.ncbi.nlm.nih.gov/pubmed?term=Silva RA%5BAuthor%5D&cauthor=true&cauthor_uid=14639127), [Fernandes J](http://www.ncbi.nlm.nih.gov/pubmed?term=Fernandes J%5BAuthor%5D&cauthor=true&cauthor_uid=14639127), [Lopes C](http://www.ncbi.nlm.nih.gov/pubmed?term=Lopes C%5BAuthor%5D&cauthor=true&cauthor_uid=14639127) (2003) CYP1A1 and XRCC1 gene polymorphisms in SCC of the larynx. [Eur J Cancer Prev](http://www.ncbi.nlm.nih.gov/pubmed/?term=CYP1A1+and+XRCC1+gene+polymorphisms+in+SCC+of+the+larynx) 12:495-499.
94. [Li R](http://www.ncbi.nlm.nih.gov/pubmed?term=Li R%5BAuthor%5D&cauthor=true&cauthor_uid=19110417), [Shugart YY](http://www.ncbi.nlm.nih.gov/pubmed?term=Shugart YY%5BAuthor%5D&cauthor=true&cauthor_uid=19110417), [Zhou W](http://www.ncbi.nlm.nih.gov/pubmed?term=Zhou W%5BAuthor%5D&cauthor=true&cauthor_uid=19110417), [An Y](http://www.ncbi.nlm.nih.gov/pubmed?term=An Y%5BAuthor%5D&cauthor=true&cauthor_uid=19110417), [Yang Y](http://www.ncbi.nlm.nih.gov/pubmed?term=Yang Y%5BAuthor%5D&cauthor=true&cauthor_uid=19110417) et al. (2009) Common genetic variations of the cytochrome P450 1A1 gene and risk of hepatocellular carcinoma in a Chinese population. [Eur J Cancer](http://www.ncbi.nlm.nih.gov/pubmed/?term=Common+genetic+variations+of+the+cytochrome+P450+1A1+gene+and+risk+of+hepatocellular+carcinoma+in+a+Chinese+population) 45:1239-1247.
95. [Yin PH](http://www.ncbi.nlm.nih.gov/pubmed?term=Yin PH%5BAuthor%5D&cauthor=true&cauthor_uid=15341023), [Lee HC](http://www.ncbi.nlm.nih.gov/pubmed?term=Lee HC%5BAuthor%5D&cauthor=true&cauthor_uid=15341023), [Chau GY](http://www.ncbi.nlm.nih.gov/pubmed?term=Chau GY%5BAuthor%5D&cauthor=true&cauthor_uid=15341023), [Liu TY](http://www.ncbi.nlm.nih.gov/pubmed?term=Liu TY%5BAuthor%5D&cauthor=true&cauthor_uid=15341023), [Liu HC](http://www.ncbi.nlm.nih.gov/pubmed?term=Liu HC%5BAuthor%5D&cauthor=true&cauthor_uid=15341023) et al. (2004) Polymorphisms of estrogen-metabolizing genes and risk of hepatocellular carcinoma in Taiwan females. [Cancer Lett](http://www.ncbi.nlm.nih.gov/pubmed/?term=Polymorphisms+of+estrogen-metabolizing+genes+and+risk+of+hepatocellular+carcinoma+in+Taiwan+females) 212:195-201.
96. [Yu MW](http://www.ncbi.nlm.nih.gov/pubmed?term=Yu MW%5BAuthor%5D&cauthor=true&cauthor_uid=10408872), [Chiu YH](http://www.ncbi.nlm.nih.gov/pubmed?term=Chiu YH%5BAuthor%5D&cauthor=true&cauthor_uid=10408872), [Yang SY](http://www.ncbi.nlm.nih.gov/pubmed?term=Yang SY%5BAuthor%5D&cauthor=true&cauthor_uid=10408872), [Santella RM](http://www.ncbi.nlm.nih.gov/pubmed?term=Santella RM%5BAuthor%5D&cauthor=true&cauthor_uid=10408872), [Chern HD](http://www.ncbi.nlm.nih.gov/pubmed?term=Chern HD%5BAuthor%5D&cauthor=true&cauthor_uid=10408872) et al. (1999) Cytochrome P450 1A1 genetic polymorphisms and risk of hepatocellular carcinoma among chronic hepatitis B carriers. [Br J Cancer](http://www.ncbi.nlm.nih.gov/pubmed/10408872) 80:598-603.
97. [Yuan X](http://www.ncbi.nlm.nih.gov/pubmed?term=Yuan X%5BAuthor%5D&cauthor=true&cauthor_uid=19064581), [Zhou G](http://www.ncbi.nlm.nih.gov/pubmed?term=Zhou G%5BAuthor%5D&cauthor=true&cauthor_uid=19064581), [Zhai Y](http://www.ncbi.nlm.nih.gov/pubmed?term=Zhai Y%5BAuthor%5D&cauthor=true&cauthor_uid=19064581), [Xie W](http://www.ncbi.nlm.nih.gov/pubmed?term=Xie W%5BAuthor%5D&cauthor=true&cauthor_uid=19064581), [Cui Y](http://www.ncbi.nlm.nih.gov/pubmed?term=Cui Y%5BAuthor%5D&cauthor=true&cauthor_uid=19064581) et al. (2008) Lack of association between the functional polymorphisms in the estrogen-metabolizing genes and risk for hepatocellular carcinoma. [Cancer Epidemiol Biomarkers Prev](http://www.ncbi.nlm.nih.gov/pubmed/?term=Lack+of+association+between+the+functional+polymorphisms+in+the+estrogen-metabolizing+genes+and+risk+for+hepatocellular+carcinoma) 17:3621-3627.
98. [Aydin-Sayitoglu M](http://www.ncbi.nlm.nih.gov/pubmed?term=Aydin-Sayitoglu M%5BAuthor%5D&cauthor=true&cauthor_uid=16493615), [Hatirnaz O](http://www.ncbi.nlm.nih.gov/pubmed?term=Hatirnaz O%5BAuthor%5D&cauthor=true&cauthor_uid=16493615), [Erensoy N](http://www.ncbi.nlm.nih.gov/pubmed?term=Erensoy N%5BAuthor%5D&cauthor=true&cauthor_uid=16493615), [Ozbek U](http://www.ncbi.nlm.nih.gov/pubmed?term=Ozbek U%5BAuthor%5D&cauthor=true&cauthor_uid=16493615) (2006) Role of CYP2D6, CYP1A1, CYP2E1, GSTT1, and GSTM1 genes in the susceptibility to acute leukemias. [Am J Hematol](http://www.ncbi.nlm.nih.gov/pubmed/?term=Role+of+CYP2D6%2C+CYP1A1%2C+CYP2E1%2C+GSTT1%2C+and+GSTM1+genes+in+the+susceptibility+to+acute+leukemias) 81:162-170.
99. [Bolufer P](http://www.ncbi.nlm.nih.gov/pubmed?term=Bolufer P%5BAuthor%5D&cauthor=true&cauthor_uid=17339179), [Collado M](http://www.ncbi.nlm.nih.gov/pubmed?term=Collado M%5BAuthor%5D&cauthor=true&cauthor_uid=17339179), [Barragán E](http://www.ncbi.nlm.nih.gov/pubmed?term=Barragán E%5BAuthor%5D&cauthor=true&cauthor_uid=17339179), [Cervera J](http://www.ncbi.nlm.nih.gov/pubmed?term=Cervera J%5BAuthor%5D&cauthor=true&cauthor_uid=17339179), [Calasanz MJ](http://www.ncbi.nlm.nih.gov/pubmed?term=Calasanz MJ%5BAuthor%5D&cauthor=true&cauthor_uid=17339179) et al. (2007) The potential effect of gender in combination with common genetic polymorphisms of drug-metabolizing enzymes on the risk of developing acute leukemia. [Haematologica](http://www.ncbi.nlm.nih.gov/pubmed/?term=The+potential+effect+of+gender+in+combination+with+common+genetic+polymorphisms+of+drug-metabolizing+enzymes+on+the+risk+of+developing+acute+leukemia) 92:308-314.
100. [Bonaventure A](http://www.ncbi.nlm.nih.gov/pubmed?term=Bonaventure A%5BAuthor%5D&cauthor=true&cauthor_uid=22200898), [Goujon-Bellec S](http://www.ncbi.nlm.nih.gov/pubmed?term=Goujon-Bellec S%5BAuthor%5D&cauthor=true&cauthor_uid=22200898), [Rudant J](http://www.ncbi.nlm.nih.gov/pubmed?term=Rudant J%5BAuthor%5D&cauthor=true&cauthor_uid=22200898), [Orsi L](http://www.ncbi.nlm.nih.gov/pubmed?term=Orsi L%5BAuthor%5D&cauthor=true&cauthor_uid=22200898), [Leverger G](http://www.ncbi.nlm.nih.gov/pubmed?term=Leverger G%5BAuthor%5D&cauthor=true&cauthor_uid=22200898) et al. (2012) Maternal smoking during pregnancy, genetic polymorphisms of metabolic enzymes, and childhood acute leukemia: the ESCALE study (SFCE). [Cancer Causes Control](http://www.ncbi.nlm.nih.gov/pubmed/?term=Maternal+smoking+during+pregnancy%2C+genetic+polymorphisms+of+metabolic+enzymes%2C+and+childhood+acute+leukemia%3A+the+ESCALE+study+(SFCE)) 23:329-345.
101. [Canalle R](http://www.ncbi.nlm.nih.gov/pubmed?term=Canalle R%5BAuthor%5D&cauthor=true&cauthor_uid=14991750), [Burim RV](http://www.ncbi.nlm.nih.gov/pubmed?term=Burim RV%5BAuthor%5D&cauthor=true&cauthor_uid=14991750), [Tone LG](http://www.ncbi.nlm.nih.gov/pubmed?term=Tone LG%5BAuthor%5D&cauthor=true&cauthor_uid=14991750), [Takahashi CS](http://www.ncbi.nlm.nih.gov/pubmed?term=Takahashi CS%5BAuthor%5D&cauthor=true&cauthor_uid=14991750) (2004) Genetic polymorphisms and susceptibility to childhood acute lymphoblastic leukemia. [Environ Mol Mutagen](http://www.ncbi.nlm.nih.gov/pubmed/14991750) 43:100-109.
102. [Clavel J](http://www.ncbi.nlm.nih.gov/pubmed?term=Clavel J%5BAuthor%5D&cauthor=true&cauthor_uid=16284498), [Bellec S](http://www.ncbi.nlm.nih.gov/pubmed?term=Bellec S%5BAuthor%5D&cauthor=true&cauthor_uid=16284498), [Rebouissou S](http://www.ncbi.nlm.nih.gov/pubmed?term=Rebouissou S%5BAuthor%5D&cauthor=true&cauthor_uid=16284498), [Ménégaux F](http://www.ncbi.nlm.nih.gov/pubmed?term=Ménégaux F%5BAuthor%5D&cauthor=true&cauthor_uid=16284498), [Feunteun J](http://www.ncbi.nlm.nih.gov/pubmed?term=Feunteun J%5BAuthor%5D&cauthor=true&cauthor_uid=16284498) et al. (2005) Childhood leukaemia, polymorphisms of metabolism enzyme genes, and interactions with maternal tobacco, coffee and alcohol consumption during pregnancy. [Eur J Cancer Prev](http://www.ncbi.nlm.nih.gov/pubmed/?term=Childhood+leukaemia%2C+polymorphisms+of+metabolism+enzyme+genes%2C+and+interactions+with+maternal+tobacco%2C+coffee+and+alcohol+consumption+during+pregnancy) 14:531-540.
103. [Gallegos-Arreola MP](http://www.ncbi.nlm.nih.gov/pubmed?term=Gallegos-Arreola MP%5BAuthor%5D&cauthor=true&cauthor_uid=15528152), [Batista-González CM](http://www.ncbi.nlm.nih.gov/pubmed?term=Batista-González CM%5BAuthor%5D&cauthor=true&cauthor_uid=15528152), [Delgado-Lamas JL](http://www.ncbi.nlm.nih.gov/pubmed?term=Delgado-Lamas JL%5BAuthor%5D&cauthor=true&cauthor_uid=15528152), [Figuera LE](http://www.ncbi.nlm.nih.gov/pubmed?term=Figuera LE%5BAuthor%5D&cauthor=true&cauthor_uid=15528152), [Puebla-Pérez AM](http://www.ncbi.nlm.nih.gov/pubmed?term=Puebla-Pérez AM%5BAuthor%5D&cauthor=true&cauthor_uid=15528152) et al. (2004) Cytochrome P4501A1 polymorphism is associated with susceptibility to acute lymphoblastic leukemia in adult Mexican patients. [Blood Cells Mol Dis](http://www.ncbi.nlm.nih.gov/pubmed/?term=Cytochrome+P4501A1+polymorphism+is+associated+with+susceptibility+to+acute+lymphoblastic+leukemia+in+adult+Mexican+patients) 33:326-329.
104. [Joseph T](http://www.ncbi.nlm.nih.gov/pubmed?term=Joseph T%5BAuthor%5D&cauthor=true&cauthor_uid=15382273), [Kusumakumary P](http://www.ncbi.nlm.nih.gov/pubmed?term=Kusumakumary P%5BAuthor%5D&cauthor=true&cauthor_uid=15382273), [Chacko P](http://www.ncbi.nlm.nih.gov/pubmed?term=Chacko P%5BAuthor%5D&cauthor=true&cauthor_uid=15382273), [Abraham A](http://www.ncbi.nlm.nih.gov/pubmed?term=Abraham A%5BAuthor%5D&cauthor=true&cauthor_uid=15382273), [Radhakrishna Pillai M](http://www.ncbi.nlm.nih.gov/pubmed?term=Radhakrishna Pillai M%5BAuthor%5D&cauthor=true&cauthor_uid=15382273) (2004) Genetic polymorphism of CYP1A1, CYP2D6, GSTM1 and GSTT1 and susceptibility to acute lymphoblastic leukaemia in Indian children. [Pediatr Blood Cancer](http://www.ncbi.nlm.nih.gov/pubmed/15382273) 43:560-567.
105. [Kim HN](http://www.ncbi.nlm.nih.gov/pubmed?term=Kim HN%5BAuthor%5D&cauthor=true&cauthor_uid=21942242), [Kim NY](http://www.ncbi.nlm.nih.gov/pubmed?term=Kim NY%5BAuthor%5D&cauthor=true&cauthor_uid=21942242), [Yu L](http://www.ncbi.nlm.nih.gov/pubmed?term=Yu L%5BAuthor%5D&cauthor=true&cauthor_uid=21942242), [Tran HT](http://www.ncbi.nlm.nih.gov/pubmed?term=Tran HT%5BAuthor%5D&cauthor=true&cauthor_uid=21942242), [Kim YK](http://www.ncbi.nlm.nih.gov/pubmed?term=Kim YK%5BAuthor%5D&cauthor=true&cauthor_uid=21942242) et al. (2012) Association of GSTT1 polymorphism with acute myeloid leukemia risk is dependent on smoking status. [Leuk Lymphoma](http://www.ncbi.nlm.nih.gov/pubmed/?term=Association+of+GSTT1+polymorphism+with+acute+myeloid+leukemia+risk+is+dependent+on+smoking+status) 53:681-687.
106. [Krajinovic M](http://www.ncbi.nlm.nih.gov/pubmed?term=Krajinovic M%5BAuthor%5D&cauthor=true&cauthor_uid=10029576), [Labuda D](http://www.ncbi.nlm.nih.gov/pubmed?term=Labuda D%5BAuthor%5D&cauthor=true&cauthor_uid=10029576), [Richer C](http://www.ncbi.nlm.nih.gov/pubmed?term=Richer C%5BAuthor%5D&cauthor=true&cauthor_uid=10029576), [Karimi S](http://www.ncbi.nlm.nih.gov/pubmed?term=Karimi S%5BAuthor%5D&cauthor=true&cauthor_uid=10029576), [Sinnett D](http://www.ncbi.nlm.nih.gov/pubmed?term=Sinnett D%5BAuthor%5D&cauthor=true&cauthor_uid=10029576) (1999) Susceptibility to childhood acute lymphoblastic leukemia: influence of CYP1A1, CYP2D6, GSTM1, and GSTT1 genetic polymorphisms. [Blood](http://www.ncbi.nlm.nih.gov/pubmed/10029576) 93:1496-1501.
107. [Lee KM](http://www.ncbi.nlm.nih.gov/pubmed?term=Lee KM%5BAuthor%5D&cauthor=true&cauthor_uid=18691756), [Ward MH](http://www.ncbi.nlm.nih.gov/pubmed?term=Ward MH%5BAuthor%5D&cauthor=true&cauthor_uid=18691756), [Han S](http://www.ncbi.nlm.nih.gov/pubmed?term=Han S%5BAuthor%5D&cauthor=true&cauthor_uid=18691756), [Ahn HS](http://www.ncbi.nlm.nih.gov/pubmed?term=Ahn HS%5BAuthor%5D&cauthor=true&cauthor_uid=18691756), [Kang HJ](http://www.ncbi.nlm.nih.gov/pubmed?term=Kang HJ%5BAuthor%5D&cauthor=true&cauthor_uid=18691756) et al. (2009) Paternal smoking, genetic polymorphisms in CYP1A1 and childhood leukemia risk. [Leuk Res](http://www.ncbi.nlm.nih.gov/pubmed/18691756) 33:250-258.
108. [Majumdar S](http://www.ncbi.nlm.nih.gov/pubmed?term=Majumdar S%5BAuthor%5D&cauthor=true&cauthor_uid=18287869), [Mondal BC](http://www.ncbi.nlm.nih.gov/pubmed?term=Mondal BC%5BAuthor%5D&cauthor=true&cauthor_uid=18287869), [Ghosh M](http://www.ncbi.nlm.nih.gov/pubmed?term=Ghosh M%5BAuthor%5D&cauthor=true&cauthor_uid=18287869), [Dey S](http://www.ncbi.nlm.nih.gov/pubmed?term=Dey S%5BAuthor%5D&cauthor=true&cauthor_uid=18287869), [Mukhopadhyay A](http://www.ncbi.nlm.nih.gov/pubmed?term=Mukhopadhyay A%5BAuthor%5D&cauthor=true&cauthor_uid=18287869) et al. (2008) Association of cytochrome P450, glutathione S-transferase and N-acetyl transferase 2 gene polymorphisms with incidence of acute myeloid leukemia. [Eur J Cancer Prev](http://www.ncbi.nlm.nih.gov/pubmed/?term=Association+of+cytochrome+P450%2C+glutathione+S-transferase+and+N-acetyl+transferase+2+gene+polymorphisms+with+incidence+of+acute+myeloid+leukemia) 17:125-132.
109. [Swinney RM](http://www.ncbi.nlm.nih.gov/pubmed?term=Swinney RM%5BAuthor%5D&cauthor=true&cauthor_uid=21586621), [Beuten J](http://www.ncbi.nlm.nih.gov/pubmed?term=Beuten J%5BAuthor%5D&cauthor=true&cauthor_uid=21586621), [Collier AB 3rd](http://www.ncbi.nlm.nih.gov/pubmed?term=Collier AB 3rd%5BAuthor%5D&cauthor=true&cauthor_uid=21586621), [Chen TT](http://www.ncbi.nlm.nih.gov/pubmed?term=Chen TT%5BAuthor%5D&cauthor=true&cauthor_uid=21586621), [Winick NJ](http://www.ncbi.nlm.nih.gov/pubmed?term=Winick NJ%5BAuthor%5D&cauthor=true&cauthor_uid=21586621) et al. (2011) Polymorphisms in CYP1A1 and ethnic-specific susceptibility to acute lymphoblastic leukemia in children. [Cancer Epidemiol Biomarkers Prev](http://www.ncbi.nlm.nih.gov/pubmed/?term=Polymorphisms+in+CYP1A1+and+ethnic-specific+susceptibility+to+acute+lymphoblastic+leukemia+in+children) 20:1537-1542.
110. [Taspinar M](http://www.ncbi.nlm.nih.gov/pubmed?term=Taspinar M%5BAuthor%5D&cauthor=true&cauthor_uid=18224491), [Aydos SE](http://www.ncbi.nlm.nih.gov/pubmed?term=Aydos SE%5BAuthor%5D&cauthor=true&cauthor_uid=18224491), [Comez O](http://www.ncbi.nlm.nih.gov/pubmed?term=Comez O%5BAuthor%5D&cauthor=true&cauthor_uid=18224491), [Elhan AH](http://www.ncbi.nlm.nih.gov/pubmed?term=Elhan AH%5BAuthor%5D&cauthor=true&cauthor_uid=18224491), [Karabulut HG](http://www.ncbi.nlm.nih.gov/pubmed?term=Karabulut HG%5BAuthor%5D&cauthor=true&cauthor_uid=18224491) et al. (2008) CYP1A1, GST gene polymorphisms and risk of chronic myeloid leukemia. [Swiss Med Wkly](http://www.ncbi.nlm.nih.gov/pubmed/?term=CYP1A1%2C+GST+gene+polymorphisms+and+risk+of+chronic+myeloid+leukemia) 138:12-17.
111. [Yamaguti GG](http://www.ncbi.nlm.nih.gov/pubmed?term=Yamaguti GG%5BAuthor%5D&cauthor=true&cauthor_uid=20962519), [Lourenço GJ](http://www.ncbi.nlm.nih.gov/pubmed?term=Lourenço GJ%5BAuthor%5D&cauthor=true&cauthor_uid=20962519), [Silveira VS](http://www.ncbi.nlm.nih.gov/pubmed?term=Silveira VS%5BAuthor%5D&cauthor=true&cauthor_uid=20962519), [Tone LG](http://www.ncbi.nlm.nih.gov/pubmed?term=Tone LG%5BAuthor%5D&cauthor=true&cauthor_uid=20962519), [Lopes LF](http://www.ncbi.nlm.nih.gov/pubmed?term=Lopes LF%5BAuthor%5D&cauthor=true&cauthor_uid=20962519) et al. (2010) Increased risk for acute lymphoblastic leukemia in children with cytochrome P450A1 (CYP1A1)- and NAD(P)H:quinone oxidoreductase 1 (NQO1)-inherited gene variants. [Acta Haematol](http://www.ncbi.nlm.nih.gov/pubmed?term=increased risk for acute lymphoblastic leukemia in children with cytochrome p450a1 (CYP1A1) and NAD (P) H quinone oxidoreductase 1 (NQO1) inherited gene variants&cmd=correctspelling) 124:182-184.
112. [Yamaguti GG](http://www.ncbi.nlm.nih.gov/pubmed?term=Yamaguti GG%5BAuthor%5D&cauthor=true&cauthor_uid=19456854), [Lourenço GJ](http://www.ncbi.nlm.nih.gov/pubmed?term=Lourenço GJ%5BAuthor%5D&cauthor=true&cauthor_uid=19456854), [Costa FF](http://www.ncbi.nlm.nih.gov/pubmed?term=Costa FF%5BAuthor%5D&cauthor=true&cauthor_uid=19456854), [Lima CS](http://www.ncbi.nlm.nih.gov/pubmed?term=Lima CS%5BAuthor%5D&cauthor=true&cauthor_uid=19456854) (2009) High risk of 'de novo' acute myeloid leukaemia in individuals with cytochrome P450 A1 (CYP1A1) and NAD(P)H:quinone oxidoreductase 1 (NQO1) gene defects. [Eur J Haematol](http://www.ncbi.nlm.nih.gov/pubmed/?term=High+risk+of+'de+novo'+acute+myeloid+leukaemia+in+individuals+with+cytochrome+P450+A1+(CYP1A1)+and+NAD(P)H%3Aquinone+oxidoreductase+1+(NQO1)+gene+defects) 83:270-272.
113. [Alexandrie AK](http://www.ncbi.nlm.nih.gov/pubmed?term=Alexandrie AK%5BAuthor%5D&cauthor=true&cauthor_uid=7923570), [Sundberg MI](http://www.ncbi.nlm.nih.gov/pubmed?term=Sundberg MI%5BAuthor%5D&cauthor=true&cauthor_uid=7923570), [Seidegård J](http://www.ncbi.nlm.nih.gov/pubmed?term=Seidegård J%5BAuthor%5D&cauthor=true&cauthor_uid=7923570), [Tornling G](http://www.ncbi.nlm.nih.gov/pubmed?term=Tornling G%5BAuthor%5D&cauthor=true&cauthor_uid=7923570), [Rannug A](http://www.ncbi.nlm.nih.gov/pubmed?term=Rannug A%5BAuthor%5D&cauthor=true&cauthor_uid=7923570) (1994) Genetic susceptibility to lung cancer with special emphasis on CYP1A1 and GSTM1: a study on host factors in relation to age at onset, gender and histological cancer types. [Carcinogenesis](http://www.ncbi.nlm.nih.gov/pubmed/?term=Genetic+susceptibility+to+lung+cancer+with+special+emphasis+on+CYP1A1+and+GSTM1%3A+a+study+on+host+factors+in+relation+to+age+at+onset%2C+gender+and+histological+cancer+types) 15:1785-1790.
114. Ch[en S](http://www.ncbi.nlm.nih.gov/pubmed?term=Chen S%5BAuthor%5D&cauthor=true&cauthor_uid=11406420), [Xue K](http://www.ncbi.nlm.nih.gov/pubmed?term=Xue K%5BAuthor%5D&cauthor=true&cauthor_uid=11406420), [Xu L](http://www.ncbi.nlm.nih.gov/pubmed?term=Xu L%5BAuthor%5D&cauthor=true&cauthor_uid=11406420), [Ma G](http://www.ncbi.nlm.nih.gov/pubmed?term=Ma G%5BAuthor%5D&cauthor=true&cauthor_uid=11406420), [Wu J](http://www.ncbi.nlm.nih.gov/pubmed?term=Wu J%5BAuthor%5D&cauthor=true&cauthor_uid=11406420) (2001) Polymorphisms of the CYP1A1 and GSTM1 genes in relation to individual susceptibility to lung carcinoma in Chinese population. [Mutat Res](http://www.ncbi.nlm.nih.gov/pubmed/11406420) 458:41-47.
115. [Cote ML](http://www.ncbi.nlm.nih.gov/pubmed?term=Cote ML%5BAuthor%5D&cauthor=true&cauthor_uid=17174438), [Wenzlaff AS](http://www.ncbi.nlm.nih.gov/pubmed?term=Wenzlaff AS%5BAuthor%5D&cauthor=true&cauthor_uid=17174438), [Bock CH](http://www.ncbi.nlm.nih.gov/pubmed?term=Bock CH%5BAuthor%5D&cauthor=true&cauthor_uid=17174438), [Land SJ](http://www.ncbi.nlm.nih.gov/pubmed?term=Land SJ%5BAuthor%5D&cauthor=true&cauthor_uid=17174438), [Santer SK](http://www.ncbi.nlm.nih.gov/pubmed?term=Santer SK%5BAuthor%5D&cauthor=true&cauthor_uid=17174438) et al. (2007) Combinations of cytochrome P-450 genotypes and risk of early-onset lung cancer in Caucasians and African Americans: a population-based study. [Lung Cancer](http://www.ncbi.nlm.nih.gov/pubmed/?term=Combinations+of+cytochrome+P-450+genotypes+and+risk+of+early-onset+lung+cancer+in+Caucasians+and+African+Americans%3A+a+population-based+study) 55:255-262.
116. [Cote ML](http://www.ncbi.nlm.nih.gov/pubmed?term=Cote ML%5BAuthor%5D&cauthor=true&cauthor_uid=19174490), [Yoo W](http://www.ncbi.nlm.nih.gov/pubmed?term=Yoo W%5BAuthor%5D&cauthor=true&cauthor_uid=19174490), [Wenzlaff AS](http://www.ncbi.nlm.nih.gov/pubmed?term=Wenzlaff AS%5BAuthor%5D&cauthor=true&cauthor_uid=19174490), [Prysak GM](http://www.ncbi.nlm.nih.gov/pubmed?term=Prysak GM%5BAuthor%5D&cauthor=true&cauthor_uid=19174490), [Santer SK](http://www.ncbi.nlm.nih.gov/pubmed?term=Santer SK%5BAuthor%5D&cauthor=true&cauthor_uid=19174490) et al. (2009) Tobacco and estrogen metabolic polymorphisms and risk of non-small cell lung cancer in women. [Carcinogenesis](http://www.ncbi.nlm.nih.gov/pubmed/?term=Tobacco+and+estrogen+metabolic+polymorphisms+and+risk+of+non-small+cell+lung+cancer+in+women) 30:626-635.
117. [Demir A](http://www.ncbi.nlm.nih.gov/pubmed?term=Demir A%5BAuthor%5D&cauthor=true&cauthor_uid=15765281), [Altin S](http://www.ncbi.nlm.nih.gov/pubmed?term=Altin S%5BAuthor%5D&cauthor=true&cauthor_uid=15765281), [Demir I](http://www.ncbi.nlm.nih.gov/pubmed?term=Demir I%5BAuthor%5D&cauthor=true&cauthor_uid=15765281), [Köksal V](http://www.ncbi.nlm.nih.gov/pubmed?term=Köksal V%5BAuthor%5D&cauthor=true&cauthor_uid=15765281), [Cetinçelik U](http://www.ncbi.nlm.nih.gov/pubmed?term=Cetinçelik U%5BAuthor%5D&cauthor=true&cauthor_uid=15765281) et al. (2005) The role of CYP1A1 Msp1 gene polymorphisms on lung cancer development in Turkey. [Tuberk Toraks](http://www.ncbi.nlm.nih.gov/pubmed/?term=The+role+of+CYP1A1+Msp1+gene+polymorphisms+on+lung+cancer+development+in+Turkey) 53:5-9.
118. [Dolzan V](http://www.ncbi.nlm.nih.gov/pubmed?term=Dolzan V%5BAuthor%5D&cauthor=true&cauthor_uid=10653131), [Rudolf Z](http://www.ncbi.nlm.nih.gov/pubmed?term=Rudolf Z%5BAuthor%5D&cauthor=true&cauthor_uid=10653131), [Breskvar K](http://www.ncbi.nlm.nih.gov/pubmed?term=Breskvar K%5BAuthor%5D&cauthor=true&cauthor_uid=10653131) (2000) Genetic polymorphism of xenobiotic metabolising enzymes in Slovenian lung cancer patients. [Pflugers Arch](http://www.ncbi.nlm.nih.gov/pubmed/?term=Genetic+polymorphism+of+xenobiotic+metabolising+enzymes+in+Slovenian+lung+cancer+patients) 439:R29-30.
119. [Drakoulis N](http://www.ncbi.nlm.nih.gov/pubmed?term=Drakoulis N%5BAuthor%5D&cauthor=true&cauthor_uid=7912124), [Cascorbi I](http://www.ncbi.nlm.nih.gov/pubmed?term=Cascorbi I%5BAuthor%5D&cauthor=true&cauthor_uid=7912124), [Brockmöller J](http://www.ncbi.nlm.nih.gov/pubmed?term=Brockmöller J%5BAuthor%5D&cauthor=true&cauthor_uid=7912124), [Gross CR](http://www.ncbi.nlm.nih.gov/pubmed?term=Gross CR%5BAuthor%5D&cauthor=true&cauthor_uid=7912124), [Roots I](http://www.ncbi.nlm.nih.gov/pubmed?term=Roots I%5BAuthor%5D&cauthor=true&cauthor_uid=7912124) (1994) Polymorphisms in the human CYP1A1 gene as susceptibility factors for lung cancer: exon-7 mutation (4889 A to G), and a T to C mutation in the 3'-flanking region. [Clin Investig](http://www.ncbi.nlm.nih.gov/pubmed/?term=Polymorphisms+in+the+human+CYP1A1+gene+as+susceptibility+factors+for+lung+cancer%3A+exon-7+mutation+(4889+A+to+G)%2C+and+a+T+to+C+mutation+in+the+3'-flanking+region) 72:240-248.
120. [Gallegos-Arreola MP](http://www.ncbi.nlm.nih.gov/pubmed?term=Gallegos-Arreola MP%5BAuthor%5D&cauthor=true&cauthor_uid=18409147), [Figuera-Villanueva LE](http://www.ncbi.nlm.nih.gov/pubmed?term=Figuera-Villanueva LE%5BAuthor%5D&cauthor=true&cauthor_uid=18409147), [Troyo-Sanroman R](http://www.ncbi.nlm.nih.gov/pubmed?term=Troyo-Sanroman R%5BAuthor%5D&cauthor=true&cauthor_uid=18409147), [Morgán-Villela G](http://www.ncbi.nlm.nih.gov/pubmed?term=Morgán-Villela G%5BAuthor%5D&cauthor=true&cauthor_uid=18409147), [Puebla-Pérez AM](http://www.ncbi.nlm.nih.gov/pubmed?term=Puebla-Pérez AM%5BAuthor%5D&cauthor=true&cauthor_uid=18409147) et al. (2008) CYP1A1 *2B and *4 polymorphisms are associated with lung cancer susceptibility in Mexican patients. [Int J Biol Markers](http://www.ncbi.nlm.nih.gov/pubmed/?term=CYP1A1+*2B+and+*4+polymorphisms+are+associated+with+lung+cancer+susceptibility+in+Mexican+patients) 23:24-30.
121. [Garcia-Closas M](http://www.ncbi.nlm.nih.gov/pubmed?term=Garcia-Closas M%5BAuthor%5D&cauthor=true&cauthor_uid=9242469), [Kelsey KT](http://www.ncbi.nlm.nih.gov/pubmed?term=Kelsey KT%5BAuthor%5D&cauthor=true&cauthor_uid=9242469), [Wiencke JK](http://www.ncbi.nlm.nih.gov/pubmed?term=Wiencke JK%5BAuthor%5D&cauthor=true&cauthor_uid=9242469), [Xu X](http://www.ncbi.nlm.nih.gov/pubmed?term=Xu X%5BAuthor%5D&cauthor=true&cauthor_uid=9242469), [Wain JC](http://www.ncbi.nlm.nih.gov/pubmed?term=Wain JC%5BAuthor%5D&cauthor=true&cauthor_uid=9242469) et al. (1997) A case-control study of cytochrome P450 1A1, glutathione S-transferase M1, cigarette smoking and lung cancer susceptibility (Massachusetts, United States). [Cancer Causes Control](http://www.ncbi.nlm.nih.gov/pubmed/?term=A+case-control+study+of+cytochrome+P450+1A1%2C+glutathione+S-transferase+M1%2C+cigarette+smoking+and+lung+cancer+susceptibility+(Massachusetts%2C+United+States)) 8:544-553.
122. [Hirvonen A](http://www.ncbi.nlm.nih.gov/pubmed?term=Hirvonen A%5BAuthor%5D&cauthor=true&cauthor_uid=7908263), [Husgafvel-Pursiainen K](http://www.ncbi.nlm.nih.gov/pubmed?term=Husgafvel-Pursiainen K%5BAuthor%5D&cauthor=true&cauthor_uid=7908263), [Anttila S](http://www.ncbi.nlm.nih.gov/pubmed?term=Anttila S%5BAuthor%5D&cauthor=true&cauthor_uid=7908263), [Karjalainen A](http://www.ncbi.nlm.nih.gov/pubmed?term=Karjalainen A%5BAuthor%5D&cauthor=true&cauthor_uid=7908263), [Vainio H](http://www.ncbi.nlm.nih.gov/pubmed?term=Vainio H%5BAuthor%5D&cauthor=true&cauthor_uid=7908263) (1993) Polymorphism in CYP1A1 and CYP2D6 genes: possible association with susceptibility to lung cancer. [Environ Health Perspect](http://www.ncbi.nlm.nih.gov/pubmed/?term=Polymorphism+in+CYP1A1+and+CYP2D6+genes%3A+possible+association+with+susceptibility+to+lung+cancer) 101:109-112.
123. [Hong YS](http://www.ncbi.nlm.nih.gov/pubmed?term=Hong YS%5BAuthor%5D&cauthor=true&cauthor_uid=9894148), [Chang JH](http://www.ncbi.nlm.nih.gov/pubmed?term=Chang JH%5BAuthor%5D&cauthor=true&cauthor_uid=9894148), [Kwon OJ](http://www.ncbi.nlm.nih.gov/pubmed?term=Kwon OJ%5BAuthor%5D&cauthor=true&cauthor_uid=9894148), [Ham YA](http://www.ncbi.nlm.nih.gov/pubmed?term=Ham YA%5BAuthor%5D&cauthor=true&cauthor_uid=9894148), [Choi JH](http://www.ncbi.nlm.nih.gov/pubmed?term=Choi JH%5BAuthor%5D&cauthor=true&cauthor_uid=9894148) (1998) Polymorphism of the CYP1A1 and glutathione-S-transferase gene in Korean lung cancer patients. [Exp Mol Med](http://www.ncbi.nlm.nih.gov/pubmed/9894148) 30:192-198.
124. [Honma HN](http://www.ncbi.nlm.nih.gov/pubmed?term=Honma HN%5BAuthor%5D&cauthor=true&cauthor_uid=19750329), [De Capitani EM](http://www.ncbi.nlm.nih.gov/pubmed?term=De Capitani EM%5BAuthor%5D&cauthor=true&cauthor_uid=19750329), [Barbeiro Ade S](http://www.ncbi.nlm.nih.gov/pubmed?term=Barbeiro Ade S%5BAuthor%5D&cauthor=true&cauthor_uid=19750329), [Costa DB](http://www.ncbi.nlm.nih.gov/pubmed?term=Costa DB%5BAuthor%5D&cauthor=true&cauthor_uid=19750329), [Morcillo A](http://www.ncbi.nlm.nih.gov/pubmed?term=Morcillo A%5BAuthor%5D&cauthor=true&cauthor_uid=19750329) et al. (2009) Polymorphism of the CYP1A1*2A gene and susceptibility to lung cancer in a Brazilian population. [J Bras Pneumol](http://www.ncbi.nlm.nih.gov/pubmed/?term=Polymorphism+of+the+CYP1A1*2A+gene+and+susceptibility+to+lung+cancer+in+a+Brazilian+population) 35:767-772.
125. [Ihsan R](http://www.ncbi.nlm.nih.gov/pubmed?term=Ihsan R%5BAuthor%5D&cauthor=true&cauthor_uid=22206016), [Chauhan PS](http://www.ncbi.nlm.nih.gov/pubmed?term=Chauhan PS%5BAuthor%5D&cauthor=true&cauthor_uid=22206016), [Mishra AK](http://www.ncbi.nlm.nih.gov/pubmed?term=Mishra AK%5BAuthor%5D&cauthor=true&cauthor_uid=22206016), [Yadav DS](http://www.ncbi.nlm.nih.gov/pubmed?term=Yadav DS%5BAuthor%5D&cauthor=true&cauthor_uid=22206016), [Kaushal M](http://www.ncbi.nlm.nih.gov/pubmed?term=Kaushal M%5BAuthor%5D&cauthor=true&cauthor_uid=22206016) et al. (2011) Multiple analytical approaches reveal distinct gene-environment interactions in smokers and non smokers in lung cancer. [PLoS One](http://www.ncbi.nlm.nih.gov/pubmed/?term=Multiple+analytical+approaches+reveal+distinct+gene-environment+interactions+in+smokers+and+non+smokers+in+lung+cancer) 6:e29431.
126. [Kelsey KT](http://www.ncbi.nlm.nih.gov/pubmed?term=Kelsey KT%5BAuthor%5D&cauthor=true&cauthor_uid=8020143), [Wiencke JK](http://www.ncbi.nlm.nih.gov/pubmed?term=Wiencke JK%5BAuthor%5D&cauthor=true&cauthor_uid=8020143), [Spitz MR](http://www.ncbi.nlm.nih.gov/pubmed?term=Spitz MR%5BAuthor%5D&cauthor=true&cauthor_uid=8020143) (1994) A race-specific genetic polymorphism in the CYP1A1 gene is not associated with lung cancer in African Americans. [Carcinogenesis](http://www.ncbi.nlm.nih.gov/pubmed/8020143) 15:1121-1124.
127. [Kihara M](http://www.ncbi.nlm.nih.gov/pubmed?term=Kihara M%5BAuthor%5D&cauthor=true&cauthor_uid=7586131), [Kihara M](http://www.ncbi.nlm.nih.gov/pubmed?term=Kihara M%5BAuthor%5D&cauthor=true&cauthor_uid=7586131), [Noda K](http://www.ncbi.nlm.nih.gov/pubmed?term=Noda K%5BAuthor%5D&cauthor=true&cauthor_uid=7586131) (1995) Risk of smoking for squamous and small cell carcinomas of the lung modulated by combinations of CYP1A1 and GSTM1 gene polymorphisms in a Japanese population. [Carcinogenesis](http://www.ncbi.nlm.nih.gov/pubmed/?term=Risk+of+smoking+for+squamous+and+small+cell+carcinomas+of+the+lung+modulated+by+combinations+of+CYP1A1+and+GSTM1+gene+polymorphisms+in+a+Japanese+population) 16:2331-2336.
128. [Kiyohara C](http://www.ncbi.nlm.nih.gov/pubmed?term=Kiyohara C%5BAuthor%5D&cauthor=true&cauthor_uid=12925969), [Wakai K](http://www.ncbi.nlm.nih.gov/pubmed?term=Wakai K%5BAuthor%5D&cauthor=true&cauthor_uid=12925969), [Mikami H](http://www.ncbi.nlm.nih.gov/pubmed?term=Mikami H%5BAuthor%5D&cauthor=true&cauthor_uid=12925969), [Sido K](http://www.ncbi.nlm.nih.gov/pubmed?term=Sido K%5BAuthor%5D&cauthor=true&cauthor_uid=12925969), [Ando M](http://www.ncbi.nlm.nih.gov/pubmed?term=Ando M%5BAuthor%5D&cauthor=true&cauthor_uid=12925969) et al. (2003) Risk modification by CYP1A1 and GSTM1 polymorphisms in the association of environmental tobacco smoke and lung cancer: a case-control study in Japanese nonsmoking women. [Int J Cancer](http://www.ncbi.nlm.nih.gov/pubmed/?term=Risk+modification+by+CYP1A1+and+GSTM1+polymorphisms+in+the+association+of+environmental+tobacco+smoke+and+lung+cancer%3A+a+case-control+study+in+Japanese+nonsmoking+women) 107:139-144.
129. [Kiyohara C](http://www.ncbi.nlm.nih.gov/pubmed?term=Kiyohara C%5BAuthor%5D&cauthor=true&cauthor_uid=22525558), [Horiuchi T](http://www.ncbi.nlm.nih.gov/pubmed?term=Horiuchi T%5BAuthor%5D&cauthor=true&cauthor_uid=22525558), [Takayama K](http://www.ncbi.nlm.nih.gov/pubmed?term=Takayama K%5BAuthor%5D&cauthor=true&cauthor_uid=22525558), [Nakanishi Y](http://www.ncbi.nlm.nih.gov/pubmed?term=Nakanishi Y%5BAuthor%5D&cauthor=true&cauthor_uid=22525558) (2012) Genetic polymorphisms involved in carcinogen metabolism and DNA repair and lung cancer risk in a Japanese population. [J Thorac Oncol](http://www.ncbi.nlm.nih.gov/pubmed/?term=Genetic+polymorphisms+involved+in+carcinogen+metabolism+and+DNA+repair+and+lung+cancer+risk+in+a+Japanese+population) 7:954-962.
130. [Kohno T](http://www.ncbi.nlm.nih.gov/pubmed?term=Kohno T%5BAuthor%5D&cauthor=true&cauthor_uid=21623257), [Kunitoh H](http://www.ncbi.nlm.nih.gov/pubmed?term=Kunitoh H%5BAuthor%5D&cauthor=true&cauthor_uid=21623257), [Mimaki S](http://www.ncbi.nlm.nih.gov/pubmed?term=Mimaki S%5BAuthor%5D&cauthor=true&cauthor_uid=21623257), [Shiraishi K](http://www.ncbi.nlm.nih.gov/pubmed?term=Shiraishi K%5BAuthor%5D&cauthor=true&cauthor_uid=21623257), [Kuchiba A](http://www.ncbi.nlm.nih.gov/pubmed?term=Kuchiba A%5BAuthor%5D&cauthor=true&cauthor_uid=21623257) et al. (2011) Contribution of the TP53, OGG1, CHRNA3, and HLA-DQA1 genes to the risk for lung squamous cell carcinoma. [J Thorac Oncol](http://www.ncbi.nlm.nih.gov/pubmed/?term=Contribution+of+the+TP53%2C+OGG1%2C+CHRNA3%2C+and+HLA-DQA1+genes+to+the+risk+for+lung+squamous+cell+carcinoma) 6:813-817.
131. [Kumar M](http://www.ncbi.nlm.nih.gov/pubmed?term=Kumar M%5BAuthor%5D&cauthor=true&cauthor_uid=19009239), [Agarwal SK](http://www.ncbi.nlm.nih.gov/pubmed?term=Agarwal SK%5BAuthor%5D&cauthor=true&cauthor_uid=19009239), [Goel SK](http://www.ncbi.nlm.nih.gov/pubmed?term=Goel SK%5BAuthor%5D&cauthor=true&cauthor_uid=19009239) (2009) Lung cancer risk in north Indian population: role of genetic polymorphisms and smoking. [Mol Cell Biochem](http://www.ncbi.nlm.nih.gov/pubmed/19009239) 322:73-79.
132. [Landi S](http://www.ncbi.nlm.nih.gov/pubmed?term=Landi S%5BAuthor%5D&cauthor=true&cauthor_uid=16006997), [Gemignani F](http://www.ncbi.nlm.nih.gov/pubmed?term=Gemignani F%5BAuthor%5D&cauthor=true&cauthor_uid=16006997), [Moreno V](http://www.ncbi.nlm.nih.gov/pubmed?term=Moreno V%5BAuthor%5D&cauthor=true&cauthor_uid=16006997), [Gioia-Patricola L](http://www.ncbi.nlm.nih.gov/pubmed?term=Gioia-Patricola L%5BAuthor%5D&cauthor=true&cauthor_uid=16006997), [Chabrier A](http://www.ncbi.nlm.nih.gov/pubmed?term=Chabrier A%5BAuthor%5D&cauthor=true&cauthor_uid=16006997) et al. (2005) A comprehensive analysis of phase I and phase II metabolism gene polymorphisms and risk of colorectal cancer. [Pharmacogenet Genomics](http://www.ncbi.nlm.nih.gov/pubmed/16006997) 15:535-546.
133. [Le Marchand L](http://www.ncbi.nlm.nih.gov/pubmed?term=Le Marchand L%5BAuthor%5D&cauthor=true&cauthor_uid=9809991), [Sivaraman L](http://www.ncbi.nlm.nih.gov/pubmed?term=Sivaraman L%5BAuthor%5D&cauthor=true&cauthor_uid=9809991), [Pierce L](http://www.ncbi.nlm.nih.gov/pubmed?term=Pierce L%5BAuthor%5D&cauthor=true&cauthor_uid=9809991), [Seifried A](http://www.ncbi.nlm.nih.gov/pubmed?term=Seifried A%5BAuthor%5D&cauthor=true&cauthor_uid=9809991), [Lum A](http://www.ncbi.nlm.nih.gov/pubmed?term=Lum A%5BAuthor%5D&cauthor=true&cauthor_uid=9809991) et al. (1998) Associations of CYP1A1, GSTM1, and CYP2E1 polymorphisms with lung cancer suggest cell type specificities to tobacco carcinogens. [Cancer Res](http://www.ncbi.nlm.nih.gov/pubmed/?term=Associations+of+CYP1A1%2C+GSTM1%2C+and+CYP2E1+polymorphisms+with+lung+cancer+suggest+cell+type+specificities+to+tobacco+carcinogens) 58:4858-4863.
134. [Li W](http://www.ncbi.nlm.nih.gov/pubmed?term=Li W%5BAuthor%5D&cauthor=true&cauthor_uid=22109568), [Yue W](http://www.ncbi.nlm.nih.gov/pubmed?term=Yue W%5BAuthor%5D&cauthor=true&cauthor_uid=22109568), [Zhang L](http://www.ncbi.nlm.nih.gov/pubmed?term=Zhang L%5BAuthor%5D&cauthor=true&cauthor_uid=22109568), [Zhao X](http://www.ncbi.nlm.nih.gov/pubmed?term=Zhao X%5BAuthor%5D&cauthor=true&cauthor_uid=22109568), [Ma L](http://www.ncbi.nlm.nih.gov/pubmed?term=Ma L%5BAuthor%5D&cauthor=true&cauthor_uid=22109568) et al. (2012) Polymorphisms in GSTM1, CYP1A1, CYP2E1, and CYP2D6 are associated with susceptibility and chemotherapy response in non-small-cell lung cancer patients. [Lung](http://www.ncbi.nlm.nih.gov/pubmed/?term=Polymorphisms+in+GSTM1%2C+CYP1A1%2C+CYP2E1%2C+and+CYP2D6+are+associated+with+susceptibility+and+chemotherapy+response+in+non-small-cell+lung+cancer+patients) 190:91-98.
135. [López-Cima MF](http://www.ncbi.nlm.nih.gov/pubmed?term=López-Cima MF%5BAuthor%5D&cauthor=true&cauthor_uid=23013535), [Alvarez-Avellón SM](http://www.ncbi.nlm.nih.gov/pubmed?term=Alvarez-Avellón SM%5BAuthor%5D&cauthor=true&cauthor_uid=23013535), [Pascual T](http://www.ncbi.nlm.nih.gov/pubmed?term=Pascual T%5BAuthor%5D&cauthor=true&cauthor_uid=23013535), [Fernández-Somoano A](http://www.ncbi.nlm.nih.gov/pubmed?term=Fernández-Somoano A%5BAuthor%5D&cauthor=true&cauthor_uid=23013535), [Tardón A](http://www.ncbi.nlm.nih.gov/pubmed?term=Tardón A%5BAuthor%5D&cauthor=true&cauthor_uid=23013535) (2012) Genetic polymorphisms in CYP1A1, GSTM1, GSTP1 and GSTT1 metabolic genes and risk of lung cancer in Asturias. [BMC Cancer](http://www.ncbi.nlm.nih.gov/pubmed/?term=Genetic+polymorphisms+in+CYP1A1%2C+GSTM1%2C+GSTP1+and+GSTT1+metabolic+genes+and+risk+of+lung+cancer+in+Asturias) 12:433.
136. [Nakachi K](http://www.ncbi.nlm.nih.gov/pubmed?term=Nakachi K%5BAuthor%5D&cauthor=true&cauthor_uid=8319207), [Imai K](http://www.ncbi.nlm.nih.gov/pubmed?term=Imai K%5BAuthor%5D&cauthor=true&cauthor_uid=8319207), [Hayashi S](http://www.ncbi.nlm.nih.gov/pubmed?term=Hayashi S%5BAuthor%5D&cauthor=true&cauthor_uid=8319207), [Kawajiri K](http://www.ncbi.nlm.nih.gov/pubmed?term=Kawajiri K%5BAuthor%5D&cauthor=true&cauthor_uid=8319207) (1993) Polymorphisms of the CYP1A1 and glutathione S-transferase genes associated with susceptibility to lung cancer in relation to cigarette dose in a Japanese population. [Cancer Res](http://www.ncbi.nlm.nih.gov/pubmed/?term=Polymorphisms+of+the+CYP1A1+and+glutathione+S-transferase+genes+associated+with+susceptibility+to+lung+cancer+in+relation+to+cigarette+dose+in+a+Japanese+population) 53:2994-2999.
137. [Ng DP](http://www.ncbi.nlm.nih.gov/pubmed?term=Ng DP%5BAuthor%5D&cauthor=true&cauthor_uid=15953982), [Tan KW](http://www.ncbi.nlm.nih.gov/pubmed?term=Tan KW%5BAuthor%5D&cauthor=true&cauthor_uid=15953982), [Zhao B](http://www.ncbi.nlm.nih.gov/pubmed?term=Zhao B%5BAuthor%5D&cauthor=true&cauthor_uid=15953982), [Seow A](http://www.ncbi.nlm.nih.gov/pubmed?term=Seow A%5BAuthor%5D&cauthor=true&cauthor_uid=15953982) (2005) CYP1A1 polymorphisms and risk of lung cancer in non-smoking Chinese women: influence of environmental tobacco smoke exposure and GSTM1/T1 genetic variation. [Cancer Causes Control](http://www.ncbi.nlm.nih.gov/pubmed/?term=CYP1A1+polymorphisms+and+risk+of+lung+cancer+in+non-smoking+Chinese+women%3A+influence+of+environmental+tobacco+smoke+exposure+and+GSTM1%2FT1+genetic+variation) 16:399-405.
138. [Oztürk O](http://www.ncbi.nlm.nih.gov/pubmed?term=Oztürk O%5BAuthor%5D&cauthor=true&cauthor_uid=14758730), [Isbir T](http://www.ncbi.nlm.nih.gov/pubmed?term=Isbir T%5BAuthor%5D&cauthor=true&cauthor_uid=14758730), [Yaylim I](http://www.ncbi.nlm.nih.gov/pubmed?term=Yaylim I%5BAuthor%5D&cauthor=true&cauthor_uid=14758730), [Kocatürk CI](http://www.ncbi.nlm.nih.gov/pubmed?term=Kocatürk CI%5BAuthor%5D&cauthor=true&cauthor_uid=14758730), [Gürses A](http://www.ncbi.nlm.nih.gov/pubmed?term=Gürses A%5BAuthor%5D&cauthor=true&cauthor_uid=14758730) (2003) GST M1 and CYP1A1 gene polymorphism and daily fruit consumption in Turkish patients with non-small cell lung carcinomas. [In Vivo](http://www.ncbi.nlm.nih.gov/pubmed/?term=GST+M1+and+CYP1A1+gene+polymorphism+and+daily+fruit+consumption+in+Turkish+patients+with+non-small+cell+lung+carcinomas) 17:625-632.
139. [Persson I](http://www.ncbi.nlm.nih.gov/pubmed?term=Persson I%5BAuthor%5D&cauthor=true&cauthor_uid=10209943), [Johansson I](http://www.ncbi.nlm.nih.gov/pubmed?term=Johansson I%5BAuthor%5D&cauthor=true&cauthor_uid=10209943), [Lou YC](http://www.ncbi.nlm.nih.gov/pubmed?term=Lou YC%5BAuthor%5D&cauthor=true&cauthor_uid=10209943), [Yue QY](http://www.ncbi.nlm.nih.gov/pubmed?term=Yue QY%5BAuthor%5D&cauthor=true&cauthor_uid=10209943), [Duan LS](http://www.ncbi.nlm.nih.gov/pubmed?term=Duan LS%5BAuthor%5D&cauthor=true&cauthor_uid=10209943) et al. (1999) Genetic polymorphism of xenobiotic metabolizing enzymes among Chinese lung cancer patients. [Int J Cancer](http://www.ncbi.nlm.nih.gov/pubmed/?term=Genetic+polymorphism+of+xenobiotic+metabolizing+enzymes+among+Chinese+lung+cancer+patients) 81:325-329.
140. [Pisani P](http://www.ncbi.nlm.nih.gov/pubmed?term=Pisani P%5BAuthor%5D&cauthor=true&cauthor_uid=16614107), [Srivatanakul P](http://www.ncbi.nlm.nih.gov/pubmed?term=Srivatanakul P%5BAuthor%5D&cauthor=true&cauthor_uid=16614107), [Randerson-Moor J](http://www.ncbi.nlm.nih.gov/pubmed?term=Randerson-Moor J%5BAuthor%5D&cauthor=true&cauthor_uid=16614107), [Vipasrinimit S](http://www.ncbi.nlm.nih.gov/pubmed?term=Vipasrinimit S%5BAuthor%5D&cauthor=true&cauthor_uid=16614107), [Lalitwongsa S](http://www.ncbi.nlm.nih.gov/pubmed?term=Lalitwongsa S%5BAuthor%5D&cauthor=true&cauthor_uid=16614107) et al. (2006) GSTM1 and CYP1A1 polymorphisms, tobacco, air pollution, and lung cancer: a study in rural Thailand. [Cancer Epidemiol Biomarkers Prev](http://www.ncbi.nlm.nih.gov/pubmed/?term=GSTM1+and+CYP1A1+polymorphisms%2C+tobacco%2C+air+pollution%2C+and+lung+cancer%3A+a+study+in+rural+Thailand) 15:667-674.
141. [Quiñones L](http://www.ncbi.nlm.nih.gov/pubmed?term=Quiñones L%5BAuthor%5D&cauthor=true&cauthor_uid=11675150), [Lucas D](http://www.ncbi.nlm.nih.gov/pubmed?term=Lucas D%5BAuthor%5D&cauthor=true&cauthor_uid=11675150), [Godoy J](http://www.ncbi.nlm.nih.gov/pubmed?term=Godoy J%5BAuthor%5D&cauthor=true&cauthor_uid=11675150), [Cáceres D](http://www.ncbi.nlm.nih.gov/pubmed?term=Cáceres D%5BAuthor%5D&cauthor=true&cauthor_uid=11675150), [Berthou F](http://www.ncbi.nlm.nih.gov/pubmed?term=Berthou F%5BAuthor%5D&cauthor=true&cauthor_uid=11675150) et al. (2001) CYP1A1, CYP2E1 and GSTM1 genetic polymorphisms. The effect of single and combined genotypes on lung cancer susceptibility in Chilean people. [Cancer Lett](http://www.ncbi.nlm.nih.gov/pubmed/?term=CYP1A1%2C+CYP2E1+and+GSTM1+genetic+polymorphisms.+The+effect+of+single+and+combined+genotypes+on+lung+cancer+susceptibility+in+Chilean+people) 174:35-44.
142. [Ratnasinghe D](http://www.ncbi.nlm.nih.gov/pubmed?term=Ratnasinghe D%5BAuthor%5D&cauthor=true&cauthor_uid=11396202), [Tangrea JA](http://www.ncbi.nlm.nih.gov/pubmed?term=Tangrea JA%5BAuthor%5D&cauthor=true&cauthor_uid=11396202), [Stewart C](http://www.ncbi.nlm.nih.gov/pubmed?term=Stewart C%5BAuthor%5D&cauthor=true&cauthor_uid=11396202), [Bhat NK](http://www.ncbi.nlm.nih.gov/pubmed?term=Bhat NK%5BAuthor%5D&cauthor=true&cauthor_uid=11396202), [Virtamo J](http://www.ncbi.nlm.nih.gov/pubmed?term=Virtamo J%5BAuthor%5D&cauthor=true&cauthor_uid=11396202) et al. (2001) Influence of antioxidants and the CYP1A1 isoleucine to valine polymorphism on the smoking--lung cancer association. [Anticancer Res](http://www.ncbi.nlm.nih.gov/pubmed?term=influence of antioxidants and the cyp1a1 isoleucine to valine polymorphism on the smoking lung cancer association&cmd=correctspelling) 21:1295-1299.
143. [Shields PG](http://www.ncbi.nlm.nih.gov/pubmed?term=Shields PG%5BAuthor%5D&cauthor=true&cauthor_uid=8220094), [Caporaso NE](http://www.ncbi.nlm.nih.gov/pubmed?term=Caporaso NE%5BAuthor%5D&cauthor=true&cauthor_uid=8220094), [Falk RT](http://www.ncbi.nlm.nih.gov/pubmed?term=Falk RT%5BAuthor%5D&cauthor=true&cauthor_uid=8220094), [Sugimura H](http://www.ncbi.nlm.nih.gov/pubmed?term=Sugimura H%5BAuthor%5D&cauthor=true&cauthor_uid=8220094), [Trivers GE](http://www.ncbi.nlm.nih.gov/pubmed?term=Trivers GE%5BAuthor%5D&cauthor=true&cauthor_uid=8220094) et al. (1993) Lung cancer, race, and a CYP1A1 genetic polymorphism. [Cancer Epidemiol Biomarkers Prev](http://www.ncbi.nlm.nih.gov/pubmed/8220094) 2:481-485.
144. [Sobti RC](http://www.ncbi.nlm.nih.gov/pubmed?term=Sobti RC%5BAuthor%5D&cauthor=true&cauthor_uid=14602525), [Sharma S](http://www.ncbi.nlm.nih.gov/pubmed?term=Sharma S%5BAuthor%5D&cauthor=true&cauthor_uid=14602525), [Joshi A](http://www.ncbi.nlm.nih.gov/pubmed?term=Joshi A%5BAuthor%5D&cauthor=true&cauthor_uid=14602525), [Jindal SK](http://www.ncbi.nlm.nih.gov/pubmed?term=Jindal SK%5BAuthor%5D&cauthor=true&cauthor_uid=14602525), [Janmeja A](http://www.ncbi.nlm.nih.gov/pubmed?term=Janmeja A%5BAuthor%5D&cauthor=true&cauthor_uid=14602525) (2003) CYP1A1 and CYP2D6 polymorphism and risk of lung cancer in a North Indian population. [Biomarkers](http://www.ncbi.nlm.nih.gov/pubmed/?term=CYP1A1+and+CYP2D6+polymorphism+and+risk+of+lung+cancer+in+a+North+Indian+population) 8:415-428.
145. [Song N](http://www.ncbi.nlm.nih.gov/pubmed?term=Song N%5BAuthor%5D&cauthor=true&cauthor_uid=11159735), [Tan W](http://www.ncbi.nlm.nih.gov/pubmed?term=Tan W%5BAuthor%5D&cauthor=true&cauthor_uid=11159735), [Xing D](http://www.ncbi.nlm.nih.gov/pubmed?term=Xing D%5BAuthor%5D&cauthor=true&cauthor_uid=11159735), [Lin D](http://www.ncbi.nlm.nih.gov/pubmed?term=Lin D%5BAuthor%5D&cauthor=true&cauthor_uid=11159735) (2001) CYP 1A1 polymorphism and risk of lung cancer in relation to tobacco smoking: a case-control study in China. [Carcinogenesis](http://www.ncbi.nlm.nih.gov/pubmed/11159735) 22:11-16.
146. [Sreeja L](http://www.ncbi.nlm.nih.gov/pubmed?term=Sreeja L%5BAuthor%5D&cauthor=true&cauthor_uid=16228113), [Syamala V](http://www.ncbi.nlm.nih.gov/pubmed?term=Syamala V%5BAuthor%5D&cauthor=true&cauthor_uid=16228113), [Hariharan S](http://www.ncbi.nlm.nih.gov/pubmed?term=Hariharan S%5BAuthor%5D&cauthor=true&cauthor_uid=16228113), [Madhavan J](http://www.ncbi.nlm.nih.gov/pubmed?term=Madhavan J%5BAuthor%5D&cauthor=true&cauthor_uid=16228113), [Devan SC](http://www.ncbi.nlm.nih.gov/pubmed?term=Devan SC%5BAuthor%5D&cauthor=true&cauthor_uid=16228113) et al. (2005) Possible risk modification by CYP1A1, GSTM1 and GSTT1 gene polymorphisms in lung cancer susceptibility in a South Indian population. [J Hum Genet](http://www.ncbi.nlm.nih.gov/pubmed/?term=Possible+risk+modification+by+CYP1A1%2C+GSTM1+and+GSTT1+gene+polymorphisms+in+lung+cancer+susceptibility+in+a+South+Indian+population) 50:618-627.
147. [Sunaga N](http://www.ncbi.nlm.nih.gov/pubmed?term=Sunaga N%5BAuthor%5D&cauthor=true&cauthor_uid=12163326), [Kohno T](http://www.ncbi.nlm.nih.gov/pubmed?term=Kohno T%5BAuthor%5D&cauthor=true&cauthor_uid=12163326), [Yanagitani N](http://www.ncbi.nlm.nih.gov/pubmed?term=Yanagitani N%5BAuthor%5D&cauthor=true&cauthor_uid=12163326), [Sugimura H](http://www.ncbi.nlm.nih.gov/pubmed?term=Sugimura H%5BAuthor%5D&cauthor=true&cauthor_uid=12163326), [Kunitoh H](http://www.ncbi.nlm.nih.gov/pubmed?term=Kunitoh H%5BAuthor%5D&cauthor=true&cauthor_uid=12163326) et al. (2002) Contribution of the NQO1 and GSTT1 polymorphisms to lung adenocarcinoma susceptibility. [Cancer Epidemiol Biomarkers Prev](http://www.ncbi.nlm.nih.gov/pubmed/?term=Contribution+of+the+NQO1+and+GSTT1+polymorphisms+to+lung+adenocarcinoma+susceptibility) 11:730-738.
148. [Taioli E](http://www.ncbi.nlm.nih.gov/pubmed?term=Taioli E%5BAuthor%5D&cauthor=true&cauthor_uid=12690010), [Gaspari L](http://www.ncbi.nlm.nih.gov/pubmed?term=Gaspari L%5BAuthor%5D&cauthor=true&cauthor_uid=12690010), [Benhamou S](http://www.ncbi.nlm.nih.gov/pubmed?term=Benhamou S%5BAuthor%5D&cauthor=true&cauthor_uid=12690010), [Boffetta P](http://www.ncbi.nlm.nih.gov/pubmed?term=Boffetta P%5BAuthor%5D&cauthor=true&cauthor_uid=12690010), [Brockmoller J](http://www.ncbi.nlm.nih.gov/pubmed?term=Brockmoller J%5BAuthor%5D&cauthor=true&cauthor_uid=12690010) et al. (2003) Polymorphisms in CYP1A1, GSTM1, GSTT1 and lung cancer below the age of 45 years. [Int J Epidemiol](http://www.ncbi.nlm.nih.gov/pubmed/?term=Polymorphisms+in+CYP1A1%2C+GSTM1%2C+GSTT1+and+lung+cancer+below+the+age+of+45+years) 32:60-63.
149. [Taioli E](http://www.ncbi.nlm.nih.gov/pubmed?term=Taioli E%5BAuthor%5D&cauthor=true&cauthor_uid=9635868), [Ford J](http://www.ncbi.nlm.nih.gov/pubmed?term=Ford J%5BAuthor%5D&cauthor=true&cauthor_uid=9635868), [Trachman J](http://www.ncbi.nlm.nih.gov/pubmed?term=Trachman J%5BAuthor%5D&cauthor=true&cauthor_uid=9635868), [Li Y](http://www.ncbi.nlm.nih.gov/pubmed?term=Li Y%5BAuthor%5D&cauthor=true&cauthor_uid=9635868), [Demopoulos R](http://www.ncbi.nlm.nih.gov/pubmed?term=Demopoulos R%5BAuthor%5D&cauthor=true&cauthor_uid=9635868) et al. (1998) Lung cancer risk and CYP1A1 genotype in African Americans. [Carcinogenesis](http://www.ncbi.nlm.nih.gov/pubmed/9635868) 19:813-817.
150. [Tefre T](http://www.ncbi.nlm.nih.gov/pubmed?term=Tefre T%5BAuthor%5D&cauthor=true&cauthor_uid=1726950), [Ryberg D](http://www.ncbi.nlm.nih.gov/pubmed?term=Ryberg D%5BAuthor%5D&cauthor=true&cauthor_uid=1726950), [Haugen A](http://www.ncbi.nlm.nih.gov/pubmed?term=Haugen A%5BAuthor%5D&cauthor=true&cauthor_uid=1726950), [Nebert DW](http://www.ncbi.nlm.nih.gov/pubmed?term=Nebert DW%5BAuthor%5D&cauthor=true&cauthor_uid=1726950), [Skaug V](http://www.ncbi.nlm.nih.gov/pubmed?term=Skaug V%5BAuthor%5D&cauthor=true&cauthor_uid=1726950) et al. (1991) Human CYP1A1 (cytochrome P(1)450) gene: lack of association between the Msp I restriction fragment length polymorphism and incidence of lung cancer in a Norwegian population. [Pharmacogenetics](http://www.ncbi.nlm.nih.gov/pubmed/?term=Human+CYP1A1+(cytochrome+P(1)450)+gene%3A+lack+of+association+between+the+Msp+I+restriction+fragment+length+polymorphism+and+incidence+of+lung+cancer+in+a+Norwegian+population) 1:20-25.
151. [Wang J](http://www.ncbi.nlm.nih.gov/pubmed?term=Wang J%5BAuthor%5D&cauthor=true&cauthor_uid=12824892), [Deng Y](http://www.ncbi.nlm.nih.gov/pubmed?term=Deng Y%5BAuthor%5D&cauthor=true&cauthor_uid=12824892), [Li L](http://www.ncbi.nlm.nih.gov/pubmed?term=Li L%5BAuthor%5D&cauthor=true&cauthor_uid=12824892), [Kuriki K](http://www.ncbi.nlm.nih.gov/pubmed?term=Kuriki K%5BAuthor%5D&cauthor=true&cauthor_uid=12824892), [Ding J](http://www.ncbi.nlm.nih.gov/pubmed?term=Ding J%5BAuthor%5D&cauthor=true&cauthor_uid=12824892) et al. (2003) Association of GSTM1, CYP1A1 and CYP2E1 genetic polymorphisms with susceptibility to lung adenocarcinoma: a case-control study in Chinese population. [Cancer Sci](http://www.ncbi.nlm.nih.gov/pubmed/?term=Association+of+GSTM1%2C+CYP1A1+and+CYP2E1+genetic+polymorphisms+with+susceptibility+to+lung+adenocarcinoma%3A+a+case-control+study+in+Chinese+population) 94:448-452.
152. [Wenzlaff AS](http://www.ncbi.nlm.nih.gov/pubmed?term=Wenzlaff AS%5BAuthor%5D&cauthor=true&cauthor_uid=16051642), [Cote ML](http://www.ncbi.nlm.nih.gov/pubmed?term=Cote ML%5BAuthor%5D&cauthor=true&cauthor_uid=16051642), [Bock CH](http://www.ncbi.nlm.nih.gov/pubmed?term=Bock CH%5BAuthor%5D&cauthor=true&cauthor_uid=16051642), [Land SJ](http://www.ncbi.nlm.nih.gov/pubmed?term=Land SJ%5BAuthor%5D&cauthor=true&cauthor_uid=16051642), [Santer SK](http://www.ncbi.nlm.nih.gov/pubmed?term=Santer SK%5BAuthor%5D&cauthor=true&cauthor_uid=16051642) et al. (2005) CYP1A1 and CYP1B1 polymorphisms and risk of lung cancer among never smokers: a population-based study. [Carcinogenesis](http://www.ncbi.nlm.nih.gov/pubmed/?term=CYP1A1+and+CYP1B1+polymorphisms+and+risk+of+lung+cancer+among+never+smokers%3A+a+population-based+study) 26:2207-2212.
153. [Wright CM](http://www.ncbi.nlm.nih.gov/pubmed?term=Wright CM%5BAuthor%5D&cauthor=true&cauthor_uid=19608585), [Larsen JE](http://www.ncbi.nlm.nih.gov/pubmed?term=Larsen JE%5BAuthor%5D&cauthor=true&cauthor_uid=19608585), [Colosimo ML](http://www.ncbi.nlm.nih.gov/pubmed?term=Colosimo ML%5BAuthor%5D&cauthor=true&cauthor_uid=19608585), [Barr JJ](http://www.ncbi.nlm.nih.gov/pubmed?term=Barr JJ%5BAuthor%5D&cauthor=true&cauthor_uid=19608585), [Chen L](http://www.ncbi.nlm.nih.gov/pubmed?term=Chen L%5BAuthor%5D&cauthor=true&cauthor_uid=19608585) et al. (2010) Genetic association study of CYP1A1 polymorphisms identifies risk haplotypes in nonsmall cell lung cancer. [Eur Respir J](http://www.ncbi.nlm.nih.gov/pubmed?term=genetic association study of cyp1a1 polymorphisms identified risk haplotype in nonsmall cell lung cancer&cmd=correctspelling) 35:152-159.
154. [Xu X](http://www.ncbi.nlm.nih.gov/pubmed?term=Xu X%5BAuthor%5D&cauthor=true&cauthor_uid=8877059), [Kelsey KT](http://www.ncbi.nlm.nih.gov/pubmed?term=Kelsey KT%5BAuthor%5D&cauthor=true&cauthor_uid=8877059), [Wiencke JK](http://www.ncbi.nlm.nih.gov/pubmed?term=Wiencke JK%5BAuthor%5D&cauthor=true&cauthor_uid=8877059), [Wain JC](http://www.ncbi.nlm.nih.gov/pubmed?term=Wain JC%5BAuthor%5D&cauthor=true&cauthor_uid=8877059), [Christiani DC](http://www.ncbi.nlm.nih.gov/pubmed?term=Christiani DC%5BAuthor%5D&cauthor=true&cauthor_uid=8877059) (1996) Cytochrome P450 CYP1A1 MspI polymorphism and lung cancer susceptibility. [Cancer Epidemiol Biomarkers Prev](http://www.ncbi.nlm.nih.gov/pubmed/8877059) 5:687-692.
155. [Yang M](http://www.ncbi.nlm.nih.gov/pubmed?term=Yang M%5BAuthor%5D&cauthor=true&cauthor_uid=17428572), [Choi Y](http://www.ncbi.nlm.nih.gov/pubmed?term=Choi Y%5BAuthor%5D&cauthor=true&cauthor_uid=17428572), [Hwangbo B](http://www.ncbi.nlm.nih.gov/pubmed?term=Hwangbo B%5BAuthor%5D&cauthor=true&cauthor_uid=17428572), [Lee JS](http://www.ncbi.nlm.nih.gov/pubmed?term=Lee JS%5BAuthor%5D&cauthor=true&cauthor_uid=17428572) (2007) Combined effects of genetic polymorphisms in six selected genes on lung cancer susceptibility. [Lung Cancer](http://www.ncbi.nlm.nih.gov/pubmed/?term=Combined+effects+of+genetic+polymorphisms+in+six+selected+genes+on+lung+cancer+susceptibility) 57:135-142.
156. [Yang XR](http://www.ncbi.nlm.nih.gov/pubmed?term=Yang XR%5BAuthor%5D&cauthor=true&cauthor_uid=15363546), [Wacholder S](http://www.ncbi.nlm.nih.gov/pubmed?term=Wacholder S%5BAuthor%5D&cauthor=true&cauthor_uid=15363546), [Xu Z](http://www.ncbi.nlm.nih.gov/pubmed?term=Xu Z%5BAuthor%5D&cauthor=true&cauthor_uid=15363546), [Dean M](http://www.ncbi.nlm.nih.gov/pubmed?term=Dean M%5BAuthor%5D&cauthor=true&cauthor_uid=15363546), [Clark V](http://www.ncbi.nlm.nih.gov/pubmed?term=Clark V%5BAuthor%5D&cauthor=true&cauthor_uid=15363546) et al. (2004) CYP1A1 and GSTM1 polymorphisms in relation to lung cancer risk in Chinese women. [Cancer Lett](http://www.ncbi.nlm.nih.gov/pubmed/15363546) 214:197-204.
157. [Yin L](http://www.ncbi.nlm.nih.gov/pubmed?term=Yin L%5BAuthor%5D&cauthor=true&cauthor_uid=11551408), [Pu Y](http://www.ncbi.nlm.nih.gov/pubmed?term=Pu Y%5BAuthor%5D&cauthor=true&cauthor_uid=11551408), [Liu TY](http://www.ncbi.nlm.nih.gov/pubmed?term=Liu TY%5BAuthor%5D&cauthor=true&cauthor_uid=11551408), [Tung YH](http://www.ncbi.nlm.nih.gov/pubmed?term=Tung YH%5BAuthor%5D&cauthor=true&cauthor_uid=11551408), [Chen KW](http://www.ncbi.nlm.nih.gov/pubmed?term=Chen KW%5BAuthor%5D&cauthor=true&cauthor_uid=11551408) et al. (2001) Genetic polymorphisms of NAD(P)H quinone oxidoreductase, CYP1A1 and microsomal epoxide hydrolase and lung cancer risk in Nanjing, China. [Lung Cancer](http://www.ncbi.nlm.nih.gov/pubmed/?term=Genetic+polymorphisms+of+NAD(P)H+quinone+oxidoreductase%2C+CYP1A1+and+microsomal+epoxide+hydrolase+and+lung+cancer+risk+in+Nanjing%2C+China) 33:133-141.
158. [Yoon KA](http://www.ncbi.nlm.nih.gov/pubmed?term=Yoon KA%5BAuthor%5D&cauthor=true&cauthor_uid=17980933), [Kim JH](http://www.ncbi.nlm.nih.gov/pubmed?term=Kim JH%5BAuthor%5D&cauthor=true&cauthor_uid=17980933), [Gil HJ](http://www.ncbi.nlm.nih.gov/pubmed?term=Gil HJ%5BAuthor%5D&cauthor=true&cauthor_uid=17980933), [Hwang H](http://www.ncbi.nlm.nih.gov/pubmed?term=Hwang H%5BAuthor%5D&cauthor=true&cauthor_uid=17980933), [Hwangbo B](http://www.ncbi.nlm.nih.gov/pubmed?term=Hwangbo B%5BAuthor%5D&cauthor=true&cauthor_uid=17980933) et al. (2008) CYP1B1, CYP1A1, MPO, and GSTP1 polymorphisms and lung cancer risk in never-smoking Korean women. [Lung Cancer](http://www.ncbi.nlm.nih.gov/pubmed/?term=CYP1B1%2C+CYP1A1%2C+MPO%2C+and+GSTP1+polymorphisms+and+lung+cancer+risk+in+never-smoking+Korean+women) 60:40-46.
159. [Al-Dayel F](http://www.ncbi.nlm.nih.gov/pubmed?term=Al-Dayel F%5BAuthor%5D&cauthor=true&cauthor_uid=18203021), [Al-Rasheed M](http://www.ncbi.nlm.nih.gov/pubmed?term=Al-Rasheed M%5BAuthor%5D&cauthor=true&cauthor_uid=18203021), [Ibrahim M](http://www.ncbi.nlm.nih.gov/pubmed?term=Ibrahim M%5BAuthor%5D&cauthor=true&cauthor_uid=18203021), [Bu R](http://www.ncbi.nlm.nih.gov/pubmed?term=Bu R%5BAuthor%5D&cauthor=true&cauthor_uid=18203021), [Bavi P](http://www.ncbi.nlm.nih.gov/pubmed?term=Bavi P%5BAuthor%5D&cauthor=true&cauthor_uid=18203021) et al. (2008) Polymorphisms of drug-metabolizing enzymes CYP1A1, GSTT and GSTP contribute to the development of diffuse large B-cell lymphoma risk in the Saudi Arabian population. [Leuk Lymphoma](http://www.ncbi.nlm.nih.gov/pubmed/?term=Polymorphisms+of+drug-metabolizing+enzymes+CYP1A1%2C+GSTT+and+GSTP+contribute+to+the+development+of+diffuse+large+B-cell+lymphoma+risk+in+the+Saudi+Arabian+population) 49:122-129.
160. [Kim HN](http://www.ncbi.nlm.nih.gov/pubmed?term=Kim HN%5BAuthor%5D&cauthor=true&cauthor_uid=19899130), [Kim NY](http://www.ncbi.nlm.nih.gov/pubmed?term=Kim NY%5BAuthor%5D&cauthor=true&cauthor_uid=19899130), [Yu L](http://www.ncbi.nlm.nih.gov/pubmed?term=Yu L%5BAuthor%5D&cauthor=true&cauthor_uid=19899130), [Kim YK](http://www.ncbi.nlm.nih.gov/pubmed?term=Kim YK%5BAuthor%5D&cauthor=true&cauthor_uid=19899130), [Lee IK](http://www.ncbi.nlm.nih.gov/pubmed?term=Lee IK%5BAuthor%5D&cauthor=true&cauthor_uid=19899130) et al. (2009) Polymorphisms of drug-metabolizing genes and risk of non-Hodgkin lymphoma. [Am J Hematol](http://www.ncbi.nlm.nih.gov/pubmed/19899130) 84:821-825.
161. [Sarmanová J](http://www.ncbi.nlm.nih.gov/pubmed?term=Sarmanová J%5BAuthor%5D&cauthor=true&cauthor_uid=11406608), [Benesová K](http://www.ncbi.nlm.nih.gov/pubmed?term=Benesová K%5BAuthor%5D&cauthor=true&cauthor_uid=11406608), [Gut I](http://www.ncbi.nlm.nih.gov/pubmed?term=Gut I%5BAuthor%5D&cauthor=true&cauthor_uid=11406608), [Nedelcheva-Kristensen V](http://www.ncbi.nlm.nih.gov/pubmed?term=Nedelcheva-Kristensen V%5BAuthor%5D&cauthor=true&cauthor_uid=11406608), [Tynková L](http://www.ncbi.nlm.nih.gov/pubmed?term=Tynková L%5BAuthor%5D&cauthor=true&cauthor_uid=11406608) et al. (2001) Genetic polymorphisms of biotransformation enzymes in patients with Hodgkin's and non-Hodgkin's lymphomas. Hum Mol Genet 10:1265-1273.
162. [Hahn M](http://www.ncbi.nlm.nih.gov/pubmed?term=Hahn M%5BAuthor%5D&cauthor=true&cauthor_uid=12110344), [Hagedorn G](http://www.ncbi.nlm.nih.gov/pubmed?term=Hagedorn G%5BAuthor%5D&cauthor=true&cauthor_uid=12110344), [Kuhlisch E](http://www.ncbi.nlm.nih.gov/pubmed?term=Kuhlisch E%5BAuthor%5D&cauthor=true&cauthor_uid=12110344), [Schackert HK](http://www.ncbi.nlm.nih.gov/pubmed?term=Schackert HK%5BAuthor%5D&cauthor=true&cauthor_uid=12110344), [Eckelt U](http://www.ncbi.nlm.nih.gov/pubmed?term=Eckelt U%5BAuthor%5D&cauthor=true&cauthor_uid=12110344) (2002) Genetic polymorphisms of drug-metabolizing enzymes and susceptibility to oral cavity cancer. [Oral Oncol](http://www.ncbi.nlm.nih.gov/pubmed/12110344) 38:486-490.
163. [Katoh T](http://www.ncbi.nlm.nih.gov/pubmed?term=Katoh T%5BAuthor%5D&cauthor=true&cauthor_uid=10521794), [Kaneko S](http://www.ncbi.nlm.nih.gov/pubmed?term=Kaneko S%5BAuthor%5D&cauthor=true&cauthor_uid=10521794), [Kohshi K](http://www.ncbi.nlm.nih.gov/pubmed?term=Kohshi K%5BAuthor%5D&cauthor=true&cauthor_uid=10521794), [Munaka M](http://www.ncbi.nlm.nih.gov/pubmed?term=Munaka M%5BAuthor%5D&cauthor=true&cauthor_uid=10521794), [Kitagawa K](http://www.ncbi.nlm.nih.gov/pubmed?term=Kitagawa K%5BAuthor%5D&cauthor=true&cauthor_uid=10521794) et al. (1999) Genetic polymorphisms of tobacco- and alcohol-related metabolizing enzymes and oral cavity cancer. [Int J Cancer](http://www.ncbi.nlm.nih.gov/pubmed?term=genetic polymorphisms of tobacco and alcohol-related metabolizing enzymes and oral cavity cancer&cmd=correctspelling) 83:606-609.
164. M[arques CF](http://www.ncbi.nlm.nih.gov/pubmed?term=Marques CF%5BAuthor%5D&cauthor=true&cauthor_uid=16488179), [Koifman S](http://www.ncbi.nlm.nih.gov/pubmed?term=Koifman S%5BAuthor%5D&cauthor=true&cauthor_uid=16488179), [Koifman RJ](http://www.ncbi.nlm.nih.gov/pubmed?term=Koifman RJ%5BAuthor%5D&cauthor=true&cauthor_uid=16488179), [Boffetta P](http://www.ncbi.nlm.nih.gov/pubmed?term=Boffetta P%5BAuthor%5D&cauthor=true&cauthor_uid=16488179), [Brennan P](http://www.ncbi.nlm.nih.gov/pubmed?term=Brennan P%5BAuthor%5D&cauthor=true&cauthor_uid=16488179) et al. (2006) Influence of CYP1A1, CYP2E1, GSTM3 and NAT2 genetic polymorphisms in oral cancer susceptibility: results from a case-control study in Rio de Janeiro. [Oral Oncol](http://www.ncbi.nlm.nih.gov/pubmed/?term=Influence+of+CYP1A1%2C+CYP2E1%2C+GSTM3+and+NAT2+genetic+polymorphisms+in+oral+cancer+susceptibility%3A+results+from+a+case-control+study+in+Rio+de+Janeiro) 42:632-637.
165. [Sato M](http://www.ncbi.nlm.nih.gov/pubmed?term=Sato M%5BAuthor%5D&cauthor=true&cauthor_uid=10506106), [Sato T](http://www.ncbi.nlm.nih.gov/pubmed?term=Sato T%5BAuthor%5D&cauthor=true&cauthor_uid=10506106), [Izumo T](http://www.ncbi.nlm.nih.gov/pubmed?term=Izumo T%5BAuthor%5D&cauthor=true&cauthor_uid=10506106), [Amagasa T](http://www.ncbi.nlm.nih.gov/pubmed?term=Amagasa T%5BAuthor%5D&cauthor=true&cauthor_uid=10506106) (1999) Genetic polymorphism of drug-metabolizing enzymes and susceptibility to oral cancer. [Carcinogenesis](http://www.ncbi.nlm.nih.gov/pubmed/10506106) 20:1927-1931.
166. [Sato M](http://www.ncbi.nlm.nih.gov/pubmed?term=Sato M%5BAuthor%5D&cauthor=true&cauthor_uid=10793329), [Sato T](http://www.ncbi.nlm.nih.gov/pubmed?term=Sato T%5BAuthor%5D&cauthor=true&cauthor_uid=10793329), [Izumo T](http://www.ncbi.nlm.nih.gov/pubmed?term=Izumo T%5BAuthor%5D&cauthor=true&cauthor_uid=10793329), [Amagasa T](http://www.ncbi.nlm.nih.gov/pubmed?term=Amagasa T%5BAuthor%5D&cauthor=true&cauthor_uid=10793329) (2000) Genetically high susceptibility to oral squamous cell carcinoma in terms of combined genotyping of CYP1A1 and GSTM1 genes. [Oral Oncol](http://www.ncbi.nlm.nih.gov/pubmed/?term=Genetically+high+susceptibility+to+oral+squamous+cell+carcinoma+in+terms+of+combined+genotyping+of+CYP1A1+and+GSTM1+genes) 36:267-271.
167. [Sugimura T](http://www.ncbi.nlm.nih.gov/pubmed?term=Sugimura T%5BAuthor%5D&cauthor=true&cauthor_uid=16393248), [Kumimoto H](http://www.ncbi.nlm.nih.gov/pubmed?term=Kumimoto H%5BAuthor%5D&cauthor=true&cauthor_uid=16393248), [Tohnai I](http://www.ncbi.nlm.nih.gov/pubmed?term=Tohnai I%5BAuthor%5D&cauthor=true&cauthor_uid=16393248), [Fukui T](http://www.ncbi.nlm.nih.gov/pubmed?term=Fukui T%5BAuthor%5D&cauthor=true&cauthor_uid=16393248), [Matsuo K](http://www.ncbi.nlm.nih.gov/pubmed?term=Matsuo K%5BAuthor%5D&cauthor=true&cauthor_uid=16393248) et al. (2006) Gene-environment interaction involved in oral carcinogenesis: molecular epidemiological study for metabolic and DNA repair gene polymorphisms. [J Oral Pathol Med](http://www.ncbi.nlm.nih.gov/pubmed/?term=Gene-environment+interaction+involved+in+oral+carcinogenesis%3A+molecular+epidemiological+study+for+metabolic+and+DNA+repair+gene+polymorphisms) 35:11-18.
168. [Tanimoto K](http://www.ncbi.nlm.nih.gov/pubmed?term=Tanimoto K%5BAuthor%5D&cauthor=true&cauthor_uid=10435155), [Hayashi S](http://www.ncbi.nlm.nih.gov/pubmed?term=Hayashi S%5BAuthor%5D&cauthor=true&cauthor_uid=10435155), [Yoshiga K](http://www.ncbi.nlm.nih.gov/pubmed?term=Yoshiga K%5BAuthor%5D&cauthor=true&cauthor_uid=10435155), [Ichikawa T](http://www.ncbi.nlm.nih.gov/pubmed?term=Ichikawa T%5BAuthor%5D&cauthor=true&cauthor_uid=10435155) (1999) Polymorphisms of the CYP1A1 and GSTM1 gene involved in oral squamous cell carcinoma in association with a cigarette dose. [Oral Oncol](http://www.ncbi.nlm.nih.gov/pubmed/?term=Polymorphisms+of+the+CYP1A1+and+GSTM1+gene+involved+in+oral+squamous+cell+carcinoma+in+association+with+a+cigarette+dose) 35:191-196.
169. [Xie H](http://www.ncbi.nlm.nih.gov/pubmed?term=Xie H%5BAuthor%5D&cauthor=true&cauthor_uid=15206494), [Hou L](http://www.ncbi.nlm.nih.gov/pubmed?term=Hou L%5BAuthor%5D&cauthor=true&cauthor_uid=15206494), [Shields PG](http://www.ncbi.nlm.nih.gov/pubmed?term=Shields PG%5BAuthor%5D&cauthor=true&cauthor_uid=15206494), [Winn DM](http://www.ncbi.nlm.nih.gov/pubmed?term=Winn DM%5BAuthor%5D&cauthor=true&cauthor_uid=15206494), [Gridley G](http://www.ncbi.nlm.nih.gov/pubmed?term=Gridley G%5BAuthor%5D&cauthor=true&cauthor_uid=15206494) et al. (2004) Metabolic polymorphisms, smoking, and oral cancer in Puerto Rico. [Oncol Res](http://www.ncbi.nlm.nih.gov/pubmed/?term=Metabolic+polymorphisms%2C+smoking%2C+and+oral+cancer+in+Puerto+Rico) 14:315-320.
170. [Heubner M](http://www.ncbi.nlm.nih.gov/pubmed?term=Heubner M%5BAuthor%5D&cauthor=true&cauthor_uid=20377136), [Wimberger P](http://www.ncbi.nlm.nih.gov/pubmed?term=Wimberger P%5BAuthor%5D&cauthor=true&cauthor_uid=20377136), [Riemann K](http://www.ncbi.nlm.nih.gov/pubmed?term=Riemann K%5BAuthor%5D&cauthor=true&cauthor_uid=20377136), [Kasimir-Bauer S](http://www.ncbi.nlm.nih.gov/pubmed?term=Kasimir-Bauer S%5BAuthor%5D&cauthor=true&cauthor_uid=20377136), [Otterbach F](http://www.ncbi.nlm.nih.gov/pubmed?term=Otterbach F%5BAuthor%5D&cauthor=true&cauthor_uid=20377136) et al. (2010) The CYP1A1 Ile462Val polymorphism and platinum resistance of epithelial ovarian neoplasms. [Oncol Res](http://www.ncbi.nlm.nih.gov/pubmed/?term=The+CYP1A1+Ile462Val+polymorphism+and+platinum+resistance+of+epithelial+ovarian+neoplasms) 18:343-347.
171. [Holt SK](http://www.ncbi.nlm.nih.gov/pubmed?term=Holt SK%5BAuthor%5D&cauthor=true&cauthor_uid=17372243), [Rossing MA](http://www.ncbi.nlm.nih.gov/pubmed?term=Rossing MA%5BAuthor%5D&cauthor=true&cauthor_uid=17372243), [Malone KE](http://www.ncbi.nlm.nih.gov/pubmed?term=Malone KE%5BAuthor%5D&cauthor=true&cauthor_uid=17372243), [Schwartz SM](http://www.ncbi.nlm.nih.gov/pubmed?term=Schwartz SM%5BAuthor%5D&cauthor=true&cauthor_uid=17372243), [Weiss NS](http://www.ncbi.nlm.nih.gov/pubmed?term=Weiss NS%5BAuthor%5D&cauthor=true&cauthor_uid=17372243) et al. (2007) Ovarian cancer risk and polymorphisms involved in estrogen catabolism. [Cancer Epidemiol Biomarkers Prev](http://www.ncbi.nlm.nih.gov/pubmed/17372243) 16:481-489.
172. [Matei MC](http://www.ncbi.nlm.nih.gov/pubmed?term=Matei MC%5BAuthor%5D&cauthor=true&cauthor_uid=22395499), [Negură L](http://www.ncbi.nlm.nih.gov/pubmed?term=Negură L%5BAuthor%5D&cauthor=true&cauthor_uid=22395499), [Liliac L](http://www.ncbi.nlm.nih.gov/pubmed?term=Liliac L%5BAuthor%5D&cauthor=true&cauthor_uid=22395499), [Negură A](http://www.ncbi.nlm.nih.gov/pubmed?term=Negură A%5BAuthor%5D&cauthor=true&cauthor_uid=22395499), [Azoicăi D](http://www.ncbi.nlm.nih.gov/pubmed?term=Azoicăi D%5BAuthor%5D&cauthor=true&cauthor_uid=22395499) (2012) Validation of PCR-RFLP techniques for the evaluation of codon 72 of p53 and CYP1A1 gene's polymorphisms in relation with ovarian cancer in a Romanian population. [Rom J Morphol Embryol](http://www.ncbi.nlm.nih.gov/pubmed/?term=Validation+of+PCR-RFLP+techniques+for+the+evaluation+of+codon+72+of+p53+and+CYP1A1+gene's+polymorphisms+in+relation+with+ovarian+cancer+in+a+Romanian+population) 53:47-54.
173. [Terry KL](http://www.ncbi.nlm.nih.gov/pubmed?term=Terry KL%5BAuthor%5D&cauthor=true&cauthor_uid=12646505), [Titus-Ernstoff L](http://www.ncbi.nlm.nih.gov/pubmed?term=Titus-Ernstoff L%5BAuthor%5D&cauthor=true&cauthor_uid=12646505), [Garner EO](http://www.ncbi.nlm.nih.gov/pubmed?term=Garner EO%5BAuthor%5D&cauthor=true&cauthor_uid=12646505), [Vitonis AF](http://www.ncbi.nlm.nih.gov/pubmed?term=Vitonis AF%5BAuthor%5D&cauthor=true&cauthor_uid=12646505), [Cramer DW](http://www.ncbi.nlm.nih.gov/pubmed?term=Cramer DW%5BAuthor%5D&cauthor=true&cauthor_uid=12646505) (2003) Interaction between CYP1A1 polymorphic variants and dietary exposures influencing ovarian cancer risk. [Cancer Epidemiol Biomarkers Prev](http://www.ncbi.nlm.nih.gov/pubmed/?term=Interaction+between+CYP1A1+polymorphic+variants+and+dietary+exposures+influencing+ovarian+cancer+risk.) 12:187-190.
174. [Li D](http://www.ncbi.nlm.nih.gov/pubmed?term=Li D%5BAuthor%5D&cauthor=true&cauthor_uid=11719088), [Firozi PF](http://www.ncbi.nlm.nih.gov/pubmed?term=Firozi PF%5BAuthor%5D&cauthor=true&cauthor_uid=11719088), [Zhang W](http://www.ncbi.nlm.nih.gov/pubmed?term=Zhang W%5BAuthor%5D&cauthor=true&cauthor_uid=11719088), [Shen J](http://www.ncbi.nlm.nih.gov/pubmed?term=Shen J%5BAuthor%5D&cauthor=true&cauthor_uid=11719088), [DiGiovanni J](http://www.ncbi.nlm.nih.gov/pubmed?term=DiGiovanni J%5BAuthor%5D&cauthor=true&cauthor_uid=11719088) et al. (2002) DNA adducts, genetic polymorphisms, and K-ras mutation in human pancreatic cancer. [Mutat Res](http://www.ncbi.nlm.nih.gov/pubmed/11719088) 513:37-48.
175. [Acevedo C](http://www.ncbi.nlm.nih.gov/pubmed?term=Acevedo C%5BAuthor%5D&cauthor=true&cauthor_uid=12949934), [Opazo JL](http://www.ncbi.nlm.nih.gov/pubmed?term=Opazo JL%5BAuthor%5D&cauthor=true&cauthor_uid=12949934), [Huidobro C](http://www.ncbi.nlm.nih.gov/pubmed?term=Huidobro C%5BAuthor%5D&cauthor=true&cauthor_uid=12949934), [Cabezas J](http://www.ncbi.nlm.nih.gov/pubmed?term=Cabezas J%5BAuthor%5D&cauthor=true&cauthor_uid=12949934), [Iturrieta J](http://www.ncbi.nlm.nih.gov/pubmed?term=Iturrieta J%5BAuthor%5D&cauthor=true&cauthor_uid=12949934) et al. (2003) Positive correlation between single or combined genotypes of CYP1A1 and GSTM1 in relation to prostate cancer in Chilean people. [Prostate](http://www.ncbi.nlm.nih.gov/pubmed/12949934) 57:111-117.
176. [Aktas D](http://www.ncbi.nlm.nih.gov/pubmed?term=Aktas D%5BAuthor%5D&cauthor=true&cauthor_uid=15381379), [Hascicek M](http://www.ncbi.nlm.nih.gov/pubmed?term=Hascicek M%5BAuthor%5D&cauthor=true&cauthor_uid=15381379), [Sozen S](http://www.ncbi.nlm.nih.gov/pubmed?term=Sozen S%5BAuthor%5D&cauthor=true&cauthor_uid=15381379), [Ozen H](http://www.ncbi.nlm.nih.gov/pubmed?term=Ozen H%5BAuthor%5D&cauthor=true&cauthor_uid=15381379), [Tuncbilek E](http://www.ncbi.nlm.nih.gov/pubmed?term=Tuncbilek E%5BAuthor%5D&cauthor=true&cauthor_uid=15381379) et al. (2004) CYP1A1 and GSTM1 polymorphic genotypes in patients with prostate cancer in a Turkish population. [Cancer Genet Cytogenet](http://www.ncbi.nlm.nih.gov/pubmed/?term=CYP1A1+and+GSTM1+polymorphic+genotypes+in+patients+with+prostate+cancer+in+a+Turkish+population) 154:81-85.
177. [Beer TM](http://www.ncbi.nlm.nih.gov/pubmed?term=Beer TM%5BAuthor%5D&cauthor=true&cauthor_uid=15195126), [Evans AJ](http://www.ncbi.nlm.nih.gov/pubmed?term=Evans AJ%5BAuthor%5D&cauthor=true&cauthor_uid=15195126), [Hough KM](http://www.ncbi.nlm.nih.gov/pubmed?term=Hough KM%5BAuthor%5D&cauthor=true&cauthor_uid=15195126), [Lowe BA](http://www.ncbi.nlm.nih.gov/pubmed?term=Lowe BA%5BAuthor%5D&cauthor=true&cauthor_uid=15195126), [McWilliams JE](http://www.ncbi.nlm.nih.gov/pubmed?term=McWilliams JE%5BAuthor%5D&cauthor=true&cauthor_uid=15195126) et al. (2002) Polymorphisms of GSTP1 and related genes and prostate cancer risk. [Prostate Cancer Prostatic Dis](http://www.ncbi.nlm.nih.gov/pubmed/15195126) 5:22-27.
178. [Cáceres DD](http://www.ncbi.nlm.nih.gov/pubmed?term=Cáceres DD%5BAuthor%5D&cauthor=true&cauthor_uid=15756908), [Iturrieta J](http://www.ncbi.nlm.nih.gov/pubmed?term=Iturrieta J%5BAuthor%5D&cauthor=true&cauthor_uid=15756908), [Acevedo C](http://www.ncbi.nlm.nih.gov/pubmed?term=Acevedo C%5BAuthor%5D&cauthor=true&cauthor_uid=15756908), [Huidobro C](http://www.ncbi.nlm.nih.gov/pubmed?term=Huidobro C%5BAuthor%5D&cauthor=true&cauthor_uid=15756908), [Varela N](http://www.ncbi.nlm.nih.gov/pubmed?term=Varela N%5BAuthor%5D&cauthor=true&cauthor_uid=15756908) et al. (2005) Relationship among metabolizing genes, smoking and alcohol used as modifier factors on prostate cancer risk: exploring some gene-gene and gene-environment interactions. [Eur J Epidemiol](http://www.ncbi.nlm.nih.gov/pubmed/?term=Relationship+among+metabolizing+genes%2C+smoking+and+alcohol+used+as+modifier+factors+on+prostate+cancer+risk%3A+exploring+some+gene-gene+and+gene-environment+interactions) 20:79-88.
179. [Chang BL](http://www.ncbi.nlm.nih.gov/pubmed?term=Chang BL%5BAuthor%5D&cauthor=true&cauthor_uid=12845676), [Zheng SL](http://www.ncbi.nlm.nih.gov/pubmed?term=Zheng SL%5BAuthor%5D&cauthor=true&cauthor_uid=12845676), [Isaacs SD](http://www.ncbi.nlm.nih.gov/pubmed?term=Isaacs SD%5BAuthor%5D&cauthor=true&cauthor_uid=12845676), [Turner A](http://www.ncbi.nlm.nih.gov/pubmed?term=Turner A%5BAuthor%5D&cauthor=true&cauthor_uid=12845676), [Hawkins GA](http://www.ncbi.nlm.nih.gov/pubmed?term=Hawkins GA%5BAuthor%5D&cauthor=true&cauthor_uid=12845676) et al. (2003) Polymorphisms in the CYP1A1 gene are associated with prostate cancer risk. [Int J Cancer](http://www.ncbi.nlm.nih.gov/pubmed/12845676) 106:375-378.
180. [Kumar V](http://www.ncbi.nlm.nih.gov/pubmed?term=Kumar V%5BAuthor%5D&cauthor=true&cauthor_uid=20817259), [Yadav CS](http://www.ncbi.nlm.nih.gov/pubmed?term=Yadav CS%5BAuthor%5D&cauthor=true&cauthor_uid=20817259), [Singh S](http://www.ncbi.nlm.nih.gov/pubmed?term=Singh S%5BAuthor%5D&cauthor=true&cauthor_uid=20817259), [Goel S](http://www.ncbi.nlm.nih.gov/pubmed?term=Goel S%5BAuthor%5D&cauthor=true&cauthor_uid=20817259), [Ahmed RS](http://www.ncbi.nlm.nih.gov/pubmed?term=Ahmed RS%5BAuthor%5D&cauthor=true&cauthor_uid=20817259) et al. (2010) CYP 1A1 polymorphism and organochlorine pesticides levels in the etiology of prostate cancer. [Chemosphere](http://www.ncbi.nlm.nih.gov/pubmed/?term=CYP+1A1+polymorphism+and+organochlorine+pesticides+levels+in+the+etiology+of+prostate+cancer) 81:464-468.
181. [Li M](http://www.ncbi.nlm.nih.gov/pubmed?term=Li M%5BAuthor%5D&cauthor=true&cauthor_uid=18304461), [Guan TY](http://www.ncbi.nlm.nih.gov/pubmed?term=Guan TY%5BAuthor%5D&cauthor=true&cauthor_uid=18304461), [Li Y](http://www.ncbi.nlm.nih.gov/pubmed?term=Li Y%5BAuthor%5D&cauthor=true&cauthor_uid=18304461), [Na YQ](http://www.ncbi.nlm.nih.gov/pubmed?term=Na YQ%5BAuthor%5D&cauthor=true&cauthor_uid=18304461) (2008) Polymorphisms of GSTM1 and CYP1A1 genes and their genetic susceptibility to prostate cancer in Chinese men. [Chin Med J (Engl)](http://www.ncbi.nlm.nih.gov/pubmed/?term=Polymorphisms+of+GSTM1+and+CYP1A1+genes+and+their+genetic+susceptibility+to+prostate+cancer+in+Chinese+men) 121:305-308.
182. [Lima MM Jr](http://www.ncbi.nlm.nih.gov/pubmed?term=Lima MM Jr%5BAuthor%5D&cauthor=true&cauthor_uid=18647550), [Oliveira MN](http://www.ncbi.nlm.nih.gov/pubmed?term=Oliveira MN%5BAuthor%5D&cauthor=true&cauthor_uid=18647550), [Granja F](http://www.ncbi.nlm.nih.gov/pubmed?term=Granja F%5BAuthor%5D&cauthor=true&cauthor_uid=18647550), [Trindade AC](http://www.ncbi.nlm.nih.gov/pubmed?term=Trindade AC%5BAuthor%5D&cauthor=true&cauthor_uid=18647550), [De Castro Santos LE](http://www.ncbi.nlm.nih.gov/pubmed?term=De Castro Santos LE%5BAuthor%5D&cauthor=true&cauthor_uid=18647550) et al. (2008) Lack of association of GSTT1, GSTM1, GSTO1, GSTP1 and CYP1A1 polymorphisms for susceptibility and outcome in Brazilian prostate cancer patients. [Folia Biol (Praha)](http://www.ncbi.nlm.nih.gov/pubmed/?term=Lack+of+association+of+GSTT1%2C+GSTM1%2C+GSTO1%2C+GSTP1+and+CYP1A1+polymorphisms+for+susceptibility+and+outcome+in+Brazilian+prostate+cancer+patients) 54:102-108.
183. [Mittal RD](http://www.ncbi.nlm.nih.gov/pubmed?term=Mittal RD%5BAuthor%5D&cauthor=true&cauthor_uid=17919073), [Srivastava DL](http://www.ncbi.nlm.nih.gov/pubmed?term=Srivastava DL%5BAuthor%5D&cauthor=true&cauthor_uid=17919073) (2007) Cytochrome P4501A1 and microsomal epoxide hydrolase gene polymorphisms: gene-environment interaction and risk of prostate cancer. [DNA Cell Biol](http://www.ncbi.nlm.nih.gov/pubmed/?term=Cytochrome+P4501A1+and+microsomal+epoxide+hydrolase+gene+polymorphisms%3A+gene-environment+interaction+and+risk+of+prostate+cancer) 26:791-798.
184. [Murata M](http://www.ncbi.nlm.nih.gov/pubmed?term=Murata M%5BAuthor%5D&cauthor=true&cauthor_uid=11275366), [Watanabe M](http://www.ncbi.nlm.nih.gov/pubmed?term=Watanabe M%5BAuthor%5D&cauthor=true&cauthor_uid=11275366), [Yamanaka M](http://www.ncbi.nlm.nih.gov/pubmed?term=Yamanaka M%5BAuthor%5D&cauthor=true&cauthor_uid=11275366), [Kubota Y](http://www.ncbi.nlm.nih.gov/pubmed?term=Kubota Y%5BAuthor%5D&cauthor=true&cauthor_uid=11275366), [Ito H](http://www.ncbi.nlm.nih.gov/pubmed?term=Ito H%5BAuthor%5D&cauthor=true&cauthor_uid=11275366) et al. (2001) Genetic polymorphisms in cytochrome P450 (CYP) 1A1, CYP1A2, CYP2E1, glutathione S-transferase (GST) M1 and GSTT1 and susceptibility to prostate cancer in the Japanese population. [Cancer Lett](http://www.ncbi.nlm.nih.gov/pubmed/?term=Genetic+polymorphisms+in+cytochrome+P450+(CYP)+1A1%2C+CYP1A2%2C+CYP2E1%2C+glutathione+S-transferase+(GST)+M1+and+GSTT1+and+susceptibility+to+prostate+cancer+in+the+Japanese+population) 165:171-177.
185. [Murata M](http://www.ncbi.nlm.nih.gov/pubmed?term=Murata M%5BAuthor%5D&cauthor=true&cauthor_uid=9861231), [Shiraishi T](http://www.ncbi.nlm.nih.gov/pubmed?term=Shiraishi T%5BAuthor%5D&cauthor=true&cauthor_uid=9861231), [Fukutome K](http://www.ncbi.nlm.nih.gov/pubmed?term=Fukutome K%5BAuthor%5D&cauthor=true&cauthor_uid=9861231), [Watanabe M](http://www.ncbi.nlm.nih.gov/pubmed?term=Watanabe M%5BAuthor%5D&cauthor=true&cauthor_uid=9861231), [Nagao M](http://www.ncbi.nlm.nih.gov/pubmed?term=Nagao M%5BAuthor%5D&cauthor=true&cauthor_uid=9861231) et al. (1998) Cytochrome P4501A1 and glutathione S-transferase M1 genotypes as risk factors for prostate cancer in Japan. [Jpn J Clin Oncol](http://www.ncbi.nlm.nih.gov/pubmed/?term=Cytochrome+P4501A1+and+glutathione+S-transferase+M1+genotypes+as+risk+factors+for+prostate+cancer+in+Japan) 28:657-660.
186. [Quiñones LA](http://www.ncbi.nlm.nih.gov/pubmed?term=Quiñones LA%5BAuthor%5D&cauthor=true&cauthor_uid=16625286), [Irarrázabal CE](http://www.ncbi.nlm.nih.gov/pubmed?term=Irarrázabal CE%5BAuthor%5D&cauthor=true&cauthor_uid=16625286), [Rojas CR](http://www.ncbi.nlm.nih.gov/pubmed?term=Rojas CR%5BAuthor%5D&cauthor=true&cauthor_uid=16625286), [Orellana CE](http://www.ncbi.nlm.nih.gov/pubmed?term=Orellana CE%5BAuthor%5D&cauthor=true&cauthor_uid=16625286), [Acevedo C](http://www.ncbi.nlm.nih.gov/pubmed?term=Acevedo C%5BAuthor%5D&cauthor=true&cauthor_uid=16625286) et al. (2006) Joint effect among p53, CYP1A1, GSTM1 polymorphism combinations and smoking on prostate cancer risk: an exploratory genotype-environment interaction study. [Asian J Androl](http://www.ncbi.nlm.nih.gov/pubmed/?term=Joint+effect+among+p53%2C+CYP1A1%2C+GSTM1+polymorphism+combinations+and+smoking+on+prostate+cancer+risk%3A+an+exploratory+genotype-environment+interaction+study) 8:349-355.
187. [Souiden Y](http://www.ncbi.nlm.nih.gov/pubmed?term=Souiden Y%5BAuthor%5D&cauthor=true&cauthor_uid=22304463), [Mahdouani M](http://www.ncbi.nlm.nih.gov/pubmed?term=Mahdouani M%5BAuthor%5D&cauthor=true&cauthor_uid=22304463), [Chaieb K](http://www.ncbi.nlm.nih.gov/pubmed?term=Chaieb K%5BAuthor%5D&cauthor=true&cauthor_uid=22304463), [Bakhrouf A](http://www.ncbi.nlm.nih.gov/pubmed?term=Bakhrouf A%5BAuthor%5D&cauthor=true&cauthor_uid=22304463), [Mahdouani K](http://www.ncbi.nlm.nih.gov/pubmed?term=Mahdouani K%5BAuthor%5D&cauthor=true&cauthor_uid=22304463) (2012) Lack of association of CYP1A1 polymorphism with prostate cancer susceptibility of Tunisian men. [Genet Test Mol Biomarkers](http://www.ncbi.nlm.nih.gov/pubmed/?term=Lack+of+association+of+CYP1A1+polymorphism+with+prostate+cancer+susceptibility+of+Tunisian+men) 16:661-666.
188. [Suzuki K](http://www.ncbi.nlm.nih.gov/pubmed?term=Suzuki K%5BAuthor%5D&cauthor=true&cauthor_uid=12767526), [Matsui H](http://www.ncbi.nlm.nih.gov/pubmed?term=Matsui H%5BAuthor%5D&cauthor=true&cauthor_uid=12767526), [Nakazato H](http://www.ncbi.nlm.nih.gov/pubmed?term=Nakazato H%5BAuthor%5D&cauthor=true&cauthor_uid=12767526), [Koike H](http://www.ncbi.nlm.nih.gov/pubmed?term=Koike H%5BAuthor%5D&cauthor=true&cauthor_uid=12767526), [Okugi H](http://www.ncbi.nlm.nih.gov/pubmed?term=Okugi H%5BAuthor%5D&cauthor=true&cauthor_uid=12767526) et al. (2003) Association of the genetic polymorphism in cytochrome P450 (CYP) 1A1 with risk of familial prostate cancer in a Japanese population: a case-control study. [Cancer Lett](http://www.ncbi.nlm.nih.gov/pubmed/?term=Association+of+the+genetic+polymorphism+in+cytochrome+P450+(CYP)+1A1+with+risk+of+familial+prostate+cancer+in+a+Japanese+population%3A+a+case-control+study) 195:177-183.
189. [Vijayalakshmi K](http://www.ncbi.nlm.nih.gov/pubmed?term=Vijayalakshmi K%5BAuthor%5D&cauthor=true&cauthor_uid=17192049), [Vettriselvi V](http://www.ncbi.nlm.nih.gov/pubmed?term=Vettriselvi V%5BAuthor%5D&cauthor=true&cauthor_uid=17192049), [Krishnan M](http://www.ncbi.nlm.nih.gov/pubmed?term=Krishnan M%5BAuthor%5D&cauthor=true&cauthor_uid=17192049), [Shroff S](http://www.ncbi.nlm.nih.gov/pubmed?term=Shroff S%5BAuthor%5D&cauthor=true&cauthor_uid=17192049), [Jayanth VR](http://www.ncbi.nlm.nih.gov/pubmed?term=Jayanth VR%5BAuthor%5D&cauthor=true&cauthor_uid=17192049) et al. (2005) Cytochrome p4501A1 gene variants as susceptibility marker for prostate cancer. [Cancer Biomark](http://www.ncbi.nlm.nih.gov/pubmed/?term=Cytochrome+p4501A1+gene+variants+as+susceptibility+marker+for+prostate+cancer) 1:251-258.
190. [Yang J](http://www.ncbi.nlm.nih.gov/pubmed?term=Yang J%5BAuthor%5D&cauthor=true&cauthor_uid=16834659), [Qian LX](http://www.ncbi.nlm.nih.gov/pubmed?term=Qian LX%5BAuthor%5D&cauthor=true&cauthor_uid=16834659), [Wu HF](http://www.ncbi.nlm.nih.gov/pubmed?term=Wu HF%5BAuthor%5D&cauthor=true&cauthor_uid=16834659), [Xu ZQ](http://www.ncbi.nlm.nih.gov/pubmed?term=Xu ZQ%5BAuthor%5D&cauthor=true&cauthor_uid=16834659), [Sui YG](http://www.ncbi.nlm.nih.gov/pubmed?term=Sui YG%5BAuthor%5D&cauthor=true&cauthor_uid=16834659) et al. (2006) Genetic polymorphisms in the cytochrome P450 1A1 and 2E1 genes, smoking, drinking and prostate cancer susceptibility: a case-control study in a Han nationality population in Southern China. [Int J Urol](http://www.ncbi.nlm.nih.gov/pubmed/?term=Genetic+polymorphisms+in+the+cytochrome+P450+1A1+and+2E1+genes%2C+smoking%2C+drinking+and+prostate+cancer+susceptibility%3A+a+case-control+study+in+a+Han+nationality+population+in+Southern+China) 13:773-780.
191. [Katoh T](http://www.ncbi.nlm.nih.gov/pubmed?term=Katoh T%5BAuthor%5D&cauthor=true&cauthor_uid=7697828), [Inatomi H](http://www.ncbi.nlm.nih.gov/pubmed?term=Inatomi H%5BAuthor%5D&cauthor=true&cauthor_uid=7697828), [Nagaoka A](http://www.ncbi.nlm.nih.gov/pubmed?term=Nagaoka A%5BAuthor%5D&cauthor=true&cauthor_uid=7697828), [Sugita A](http://www.ncbi.nlm.nih.gov/pubmed?term=Sugita A%5BAuthor%5D&cauthor=true&cauthor_uid=7697828) (1995) Cytochrome P4501A1 gene polymorphism and homozygous deletion of the glutathione S-transferase M1 gene in urothelial cancer patients. [Carcinogenesis](http://www.ncbi.nlm.nih.gov/pubmed/?term=Cytochrome+P4501A1+gene+polymorphism+and+homozygous+deletion+of+the+glutathione+S-transferase+M1+gene+in+urothelial+cancer+patients) 16:655-657.
192. [Wang G](http://www.ncbi.nlm.nih.gov/pubmed?term=Wang G%5BAuthor%5D&cauthor=true&cauthor_uid=22000673), [Hou J](http://www.ncbi.nlm.nih.gov/pubmed?term=Hou J%5BAuthor%5D&cauthor=true&cauthor_uid=22000673), [Ma L](http://www.ncbi.nlm.nih.gov/pubmed?term=Ma L%5BAuthor%5D&cauthor=true&cauthor_uid=22000673), [Xie J](http://www.ncbi.nlm.nih.gov/pubmed?term=Xie J%5BAuthor%5D&cauthor=true&cauthor_uid=22000673), [Yin J](http://www.ncbi.nlm.nih.gov/pubmed?term=Yin J%5BAuthor%5D&cauthor=true&cauthor_uid=22000673) et al. (2012) Risk factor for clear cell renal cell carcinoma in Chinese population: a case-control study. [Cancer Epidemiol](http://www.ncbi.nlm.nih.gov/pubmed/22000673) 36:177-182.
193. [Lira MG](http://www.ncbi.nlm.nih.gov/pubmed?term=Lira MG%5BAuthor%5D&cauthor=true&cauthor_uid=17083362), [Provezza L](http://www.ncbi.nlm.nih.gov/pubmed?term=Provezza L%5BAuthor%5D&cauthor=true&cauthor_uid=17083362), [Malerba G](http://www.ncbi.nlm.nih.gov/pubmed?term=Malerba G%5BAuthor%5D&cauthor=true&cauthor_uid=17083362), [Naldi L](http://www.ncbi.nlm.nih.gov/pubmed?term=Naldi L%5BAuthor%5D&cauthor=true&cauthor_uid=17083362), [Remuzzi G](http://www.ncbi.nlm.nih.gov/pubmed?term=Remuzzi G%5BAuthor%5D&cauthor=true&cauthor_uid=17083362) et al. (2006) Glutathione S-transferase and CYP1A1 gene polymorphisms and non-melanoma skin cancer risk in Italian transplanted patients. [Exp Dermatol](http://www.ncbi.nlm.nih.gov/pubmed/?term=Glutathione+S-transferase+and+CYP1A1+gene+polymorphisms+and+non-melanoma+skin+cancer+risk+in+Italian+transplanted+patients) 15:958-965.
194. [Yengi L](http://www.ncbi.nlm.nih.gov/pubmed?term=Yengi L%5BAuthor%5D&cauthor=true&cauthor_uid=8616834), [Inskip A](http://www.ncbi.nlm.nih.gov/pubmed?term=Inskip A%5BAuthor%5D&cauthor=true&cauthor_uid=8616834), [Gilford J](http://www.ncbi.nlm.nih.gov/pubmed?term=Gilford J%5BAuthor%5D&cauthor=true&cauthor_uid=8616834), [Alldersea J](http://www.ncbi.nlm.nih.gov/pubmed?term=Alldersea J%5BAuthor%5D&cauthor=true&cauthor_uid=8616834), [Bailey L](http://www.ncbi.nlm.nih.gov/pubmed?term=Bailey L%5BAuthor%5D&cauthor=true&cauthor_uid=8616834) et al. (1996) Polymorphism at the glutathione S-transferase locus GSTM3: interactions with cytochrome P450 and glutathione S-transferase genotypes as risk factors for multiple cutaneous basal cell carcinoma. [Cancer Res](http://www.ncbi.nlm.nih.gov/pubmed/?term=Polymorphism+at+the+glutathione+S-transferase+locus+GSTM3%3A+interactions+with+cytochrome+P450+and+glutathione+S-transferase+genotypes+as+risk+factors+for+multiple+cutaneous+basal+cell+carcinoma) 56:1974-1977.
195. [Ferlin A](http://www.ncbi.nlm.nih.gov/pubmed?term=Ferlin A%5BAuthor%5D&cauthor=true&cauthor_uid=19776291), [Ganz F](http://www.ncbi.nlm.nih.gov/pubmed?term=Ganz F%5BAuthor%5D&cauthor=true&cauthor_uid=19776291), [Pengo M](http://www.ncbi.nlm.nih.gov/pubmed?term=Pengo M%5BAuthor%5D&cauthor=true&cauthor_uid=19776291), [Selice R](http://www.ncbi.nlm.nih.gov/pubmed?term=Selice R%5BAuthor%5D&cauthor=true&cauthor_uid=19776291), [Frigo AC](http://www.ncbi.nlm.nih.gov/pubmed?term=Frigo AC%5BAuthor%5D&cauthor=true&cauthor_uid=19776291) et al. (2010) Association of testicular germ cell tumor with polymorphisms in estrogen receptor and steroid metabolism genes. [Endocr Relat Cancer](http://www.ncbi.nlm.nih.gov/pubmed/?term=Association+of+testicular+germ+cell+tumor+with+polymorphisms+in+estrogen+receptor+and+steroid+metabolism+genes) 17:17-25.
196. [Figueroa JD](http://www.ncbi.nlm.nih.gov/pubmed?term=Figueroa JD%5BAuthor%5D&cauthor=true&cauthor_uid=18415690), [Sakoda LC](http://www.ncbi.nlm.nih.gov/pubmed?term=Sakoda LC%5BAuthor%5D&cauthor=true&cauthor_uid=18415690), [Graubard BI](http://www.ncbi.nlm.nih.gov/pubmed?term=Graubard BI%5BAuthor%5D&cauthor=true&cauthor_uid=18415690), [Chanock S](http://www.ncbi.nlm.nih.gov/pubmed?term=Chanock S%5BAuthor%5D&cauthor=true&cauthor_uid=18415690), [Rubertone MV](http://www.ncbi.nlm.nih.gov/pubmed?term=Rubertone MV%5BAuthor%5D&cauthor=true&cauthor_uid=18415690) et al. (2008) Genetic variation in hormone metabolizing genes and risk of testicular germ cell tumors. [Cancer Causes Control](http://www.ncbi.nlm.nih.gov/pubmed/?term=Genetic+variation+in+hormone+metabolizing+genes+and+risk+of+testicular+germ+cell+tumors) 19:917-929.
197. [Kristiansen W](http://www.ncbi.nlm.nih.gov/pubmed?term=Kristiansen W%5BAuthor%5D&cauthor=true&cauthor_uid=20345875), [Haugen TB](http://www.ncbi.nlm.nih.gov/pubmed?term=Haugen TB%5BAuthor%5D&cauthor=true&cauthor_uid=20345875), [Witczak O](http://www.ncbi.nlm.nih.gov/pubmed?term=Witczak O%5BAuthor%5D&cauthor=true&cauthor_uid=20345875), [Andersen JM](http://www.ncbi.nlm.nih.gov/pubmed?term=Andersen JM%5BAuthor%5D&cauthor=true&cauthor_uid=20345875), [Fosså SD](http://www.ncbi.nlm.nih.gov/pubmed?term=Fosså SD%5BAuthor%5D&cauthor=true&cauthor_uid=20345875) et al. (2011) CYP1A1, CYP3A5 and CYP3A7 polymorphisms and testicular cancer susceptibility. [Int J Androl](http://www.ncbi.nlm.nih.gov/pubmed/?term=CYP1A1%2C+CYP3A5+and+CYP3A7+polymorphisms+and+testicular+cancer+susceptibility) 34:77-83.
198. [Siraj AK](http://www.ncbi.nlm.nih.gov/pubmed?term=Siraj AK%5BAuthor%5D&cauthor=true&cauthor_uid=18601742), [Ibrahim M](http://www.ncbi.nlm.nih.gov/pubmed?term=Ibrahim M%5BAuthor%5D&cauthor=true&cauthor_uid=18601742), [Al-Rasheed M](http://www.ncbi.nlm.nih.gov/pubmed?term=Al-Rasheed M%5BAuthor%5D&cauthor=true&cauthor_uid=18601742), [Abubaker J](http://www.ncbi.nlm.nih.gov/pubmed?term=Abubaker J%5BAuthor%5D&cauthor=true&cauthor_uid=18601742), [Bu R](http://www.ncbi.nlm.nih.gov/pubmed?term=Bu R%5BAuthor%5D&cauthor=true&cauthor_uid=18601742) et al. (2008) Polymorphisms of selected xenobiotic genes contribute to the development of papillary thyroid cancer susceptibility in Middle Eastern population. [BMC Med Genet](http://www.ncbi.nlm.nih.gov/pubmed/?term=Polymorphisms+of+selected+xenobiotic+genes+contribute+to+the+development+of+papillary+thyroid+cancer+susceptibility+in+Middle+Eastern+population) 9:61.
