# Supplementary material for: MspI and Ile462Val Polymorphisms in CYP1A1 and Overall Cancer Risk: A Meta-Analysis
Source: PLoS One. 2013 Dec 31;8(12):e85166. doi: 10.1371/journal.pone.0085166 (PMC3877352; doi:10.1371/journal.pone.0085166)
Supplement: Table S1 — Stratification analyses of the P value and 95% confidence interval for MspI polymorphism. (DOC) [file pone.0085166.s003.doc]

| **Table S1. Stratification analyses of the CAP1A1 MspI C/T polymorphism on cancer** | | | | | | | | |  |  |  |  |  |
| --- | --- | --- | --- | --- | --- | --- | --- | --- | --- | --- | --- | --- | --- |
|  | | | | | | | | |  |  |  |  |  |
| **Variables** | **Sample size** | | | **CvsT** | | **CCvsTT** | | **CCvsCT** | | **CCvsCTTT** | | **CCCTvsTT** |  |
|  | **n**^a^ | **case** | **con** | **OR(95% CI)** | ***P***^b^ | **OR(95% CI)** | ***P***^b^ | **OR(95% CI)** | ***P***^b^ | **OR(95% CI)** | ***P***^b^ | **OR(95% CI)** | ***P***^b^ |
| **Total** | 148 | 37783 | 50536 | **1.15(1.09-1.22)** | <0.001 | **1.33(1.17-1.51)** | <0.001 | **1.14(1.03-1.27)** | <0.001 | **1.24(1.11-1.39)** | <0.001 | **1.17(1.10-1.24)** | <0.001 |
| **Tumor type** |  |  |  |  |  |  |  |  |  |  |  |  |  |
| Prostate cancer | 10 | 1226 | 1304 | 1.08(0.81-1.45) | <0.001 | 1.07(0.68-1.69) | 0.086 | 0.74(0.52-1.06) | 0.241 | 0.87(0.56-1.34) | 0.042 | 1.26(0.90-1.75) | 0.001 |
| Ovarian cancer | 5 | 911 | 1278 | 1.04(0.80-1.35) | 0.172 | 0.92(0.44-1.95) | 0.518 | 0.99(0.46-2.13) | 0.664 | 0.94(0.44-1.97) | 0.575 | 1.04(0.78-1.40) | 0.162 |
| Breast cancer | 29 | 11912 | 15961 | 1.13(1.01-1.27) | <0.001 | 1.28(0.99-1.66) | <0.001 | 1.18(0.98-1.43) | 0.005 | 1.24(0.99-1.54) | <0.001 | 1.12(0.98-1.27) | <0.001 |
| Lung cancer | 32 | 7061 | 8392 | 1.18(1.09-1.27) | 0.019 | 1.43(1.16-1.78) | 0.017 | 1.20(0.98-1.48) | 0.023 | 1.32(1.07-1.62) | 0.008 | 1.21(1.10-1.32) | 0.102 |
| Leukemia | 15 | 2731 | 5003 | 1.37(1.07-1.75) | <0.001 | 1.87(1.03-3.40) | <0.001 | 1.48(0.82-2.66) | <0.001 | 1.72(0.95-3.12) | <0.001 | 1.36(1.08-1.70) | <0.001 |
| Colorectal cancer | 9 | 4801 | 5629 | 0.98(0.86-1.11) | 0.043 | 0.82(0.53-1.26) | 0.014 | 0.93(0.60-1.45) | 0.009 | 0.88(0.60-1.28) | 0.034 | 0.96(0.80-1.16) | 0.005 |
| Gastric cancer | 4 | 534 | 1321 | 0.88(0.67-1.14) | 0.168 | 0.81(0.32-2.07) | 0.145 | 0.77(0.36-1.66) | 0.251 | 0.79(0.34-1.84) | 0.178 | 0.88(0.65-1.18) | 0.249 |
| Head and neck cancer | 10 | 2266 | 2166 | 1.30(1.08-1.58) | 0.007 | 1.33(0.64-2.79) | <0.001 | 1.07(0.58-1.99) | 0.016 | 1.26(0.65-2.44) | 0.003 | 1.36(1.13-1.65) | 0.065 |
| Esophageal carcinoma | 4 | 476 | 967 | 1.08(0.81-1.42) | 0.079 | 1.04(0.69-1.57) | 0.888 | 1.03(0.69-1.54) | 0.935 | 1.04(0.71-1.53) | 0.963 | 1.14(0.75-1.72) | 0.033 |
| Endometrial cancer | 7 | 1390 | 2159 | 0.90(0.64-1.27) | <0.001 | 0.82(0.30-2.31) | 0.048 | 1.07(0.48-2.37) | 0.234 | 0.90(0.35-2.30) | 0.090 | 0.87(0.60-1.26) | 0.001 |
| Lymphoma | 3 | 1089 | 2628 | 0.88(0.67-1.15) | 0.066 | 1.00(0.77-1.29) | 0.421 | 0.90(0.70-1.15) | 0.903 | 0.94(0.74-1.19) | 0.616 | 0.88(0.63-1.24) | 0.043 |
| Cervical cancer | 6 | 659 | 680 | 1.50(0.94-2.40) | <0.001 | 3.12(1.39-6.99) | 0.058 | 1.79(1.11-2.88) | 0.859 | 2.48(1.41-4.36) | 0.272 | 1.54(0.86-2.76) | <0.001 |
| Hepatocellular cancer | 3 | 431 | 680 | 1.20(0.92-1.57) | 0.168 | 1.35(0.83-2.20) | 0.236 | 0.94(1.03-1.27) | 0.972 | 1.05(0.76-1.45) | 0.752 | 1.17(1.10-1.24) | 0.091 |
| Others | 11 | 2296 | 2368 | 1.25(0.98-1.60) | <0.001 | 1.58(1.02-2.44) | 0.054 | 1.27(0.93-1.74) | 0.380 | 1.43(1.00-2.05) | 0.167 | 1.29(0.96-1.74) | <0.001 |
| **Ethnicity** |  |  |  |  |  |  |  |  |  |  |  |  |  |
| Caucasian | 55 | 16070 | 21538 | 1.07(0.98-1.17) | <0.001 | 0.90(0.70-1.17) | 0.051 | 0.83(0.64-1.09) | 0.025 | 0.88(0.68-1.15) | 0.017 | 1.10(0.99-1.21) | <0.001 |
| Asian | 59 | 11445 | 16107 | 1.21(1.12-1.32) | <0.001 | 1.45(1.24-1.69) | <0.001 | 1.17(1.04-1.32) | <0.001 | 1.30(1.14-1.49) | <0.001 | 1.26(1.13-1.39) | <0.001 |
| African | 7 | 864 | 1136 | 0.96(0.76-1.22) | 0.096 | 1.09(0.65-1.83) | 0.238 | 1.11(0.73-1.70) | 0.763 | 1.24(1.11-1.39) | 0.410 | 0.93(0.73-1.19) | 0.223 |
| Mixed | 27 | 9404 | 11755 | 1.17(1.01-1.36) | <0.001 | 1.53(1.06-2.21) | <0.001 | 1.35(1.00-1.83) | <0.001 | 1.44(1.03-2.02) | <0.001 | 1.16(1.01-1.35) | <0.001 |
| **Control source** | |  |  |  |  |  |  |  |  |  |  |  |  |
| Hospital based | 74 | 14657 | 17204 | 1.15(1.06-1.24) | <0.001 | 1.28(1.08-1.53) | <0.001 | 1.10(0.96-1.26) | <0.001 | 1.19(1.02-1.39) | <0.001 | 1.18(1.07-1.29) | <0.001 |
| Population based | 70 | 21667 | 31667 | 1.14(1.05-1.24) | <0.001 | 1.32(1.09-1.60) | <0.001 | 1.15(0.98-1.36) | <0.001 | 1.24(1.04-1.48) | <0.001 | 1.14(1.05-1.25) | <0.001 |
| Mixed | 4 | 1459 | 1665 | 1.37(1.19-1.58) | 0.675 | 1.95(1.32-2.87) | 0.358 | 1.41(1.01-1.97) | 0.358 | 1.67(1.13-2.46) | 0.261 | 1.43(1.19-1.71) | 0.790 |
| **Sample size(both cases and controls)** | | | | |  |  |  |  |  |  |  |  |  |
| <500 | 102 | 12464 | 15678 | 1.21(1.11-1.31) | <0.001 | 1.44(1.21-1.72) | <0.001 | 1.22(1.05-1.41) | <0.001 | 1.33(1.14-1.56) | <0.001 | 1.23(1.12-1.36) | <0.001 |
| ≥500^c^ | 46 | 25319 | 34858 | 1.07(0.99-1.15) | <0.001 | 1.17(0.97-1.41) | <0.001 | 1.04(0.90-1.20) | 0.001 | 1.11(0.94-1.31) | <0.001 | 1.08(1.10-1.24) | <0.001 |
| ^a^Number of studies.  ***P***^b^ The value of heterogeneity test.  ^c^Stratified according to subjects ≥500 in both case and control groups or not. | | | | | | | | | | | | | |
