# Supplementary material for: MspI and Ile462Val Polymorphisms in CYP1A1 and Overall Cancer Risk: A Meta-Analysis
Source: PLoS One. 2013 Dec 31;8(12):e85166. doi: 10.1371/journal.pone.0085166 (PMC3877352; doi:10.1371/journal.pone.0085166)
Supplement: Table S2 — Stratification analyses of the P value and 95% confidence interval for Ile462Val polymorphism. (DOC) [file pone.0085166.s004.doc]

| **Table S2. Stratification analyses of the CYP1A1 Ile462Val G/A polymorphism on cancer** | | | | | | | | | | | | | | | |
| --- | --- | --- | --- | --- | --- | --- | --- | --- | --- | --- | --- | --- | --- | --- | --- |
|  | | | | | | | | | | | | | | | |
| **Variables** | | **Sample size** | | | | **GvsA** | | **GGvsAA** | | **GGvsGA** | | **GGvsGAAA** | | **GGGAvsAA** | |
|  | | **n** | **case** | | **control** | **OR(95% CI)** | ***P***^b^ | **OR(95% CI)** | ***P***^b^ | **OR(95% CI)** | ***P***^b^ | **OR(95% CI)** | ***P***^b^ | **OR(95% CI)** | ***P***^b^ |
| **Total** | | 134 | 34466 | | 44371 | **1.18(1.12-1.25)** | <0.001 | **1.52(1.34-1.72)** | <0.001 | **1.28(1.17-1.39)** | 0.093 | **1.42(1.27-1.60)** | 0.002 | **1.18(1.11-1.26)** | <0.001 |
| **Tumor type** | |  |  | |  |  |  |  |  |  |  |  |  |  |  |
| Esophageal carcinoma | | 10 | 1294 | | 1976 | 1.44(1.27-1.62) | 0.468 | 2.20(1.62-2.99) | 0.518 | 1.51(1.13-2.01) | 0.661 | 1.87(1.41-2.47) | 0.642 | 1.50(1.28-1.75) | 0.706 |
| Ovarian cancer | | 4 | 617 | | 642 | 0.94(0.49-1.80) | 0.077 | 2.12(0.37-12.02) | 0.704 | 3.20(0.55-18.66) | 0.921 | 2.20(0.39-12.47) | 0.742 | 0.88(0.43-1.81) | 0.069 |
| Lung cancer | | 26 | 6273 | | 7478 | 1.21(1.08-1.36) | <0.001 | 1.75(1.29-2.38) | 0.011 | 1.47(1.20-1.80) | 0.045 | 1.62(1.21-2.18) | 0.019 | 1.19(1.05-1.35) | 0.002 |
| Leukemia | | 8 | 1230 | | 2680 | 1.52(1.08-2.13) | <0.001 | 1.82(1.08-3.06) | 0.094 | 1.53(1.08-2.18) | 0.589 | 1.70(1.10-2.63) | 0.222 | 1.56(1.03-2.34) | <0.001 |
| Breast cancer | | 25 | 11361 | | 13557 | 1.02(0.91-1.15) | <0.001 | 1.20(0.87-1.64) | 0.003 | 1.00(0.83-1.20) | 0.195 | 1.15(0.85-1.54) | 0.010 | 1.01(0.89-1.14) | 0.001 |
| Head and neck cancer | | 12 | 2865 | | 2659 | 1.16(0.99-1.36) | 0.190 | 1.43(0.86-2.40) | 0.346 | 1.30(0.83-2.03) | 0.335 | 1.41(0.86-2.29) | 0.378 | 1.17(0.98-1.40) | 0.159 |
| Colorectal cancer | | 8 | 2656 | | 3089 | 1.33(0.97-1.83) | <0.001 | 1.58(0.98-2.54) | 0.111 | 1.59(1.21-2.09) | 0.476 | 1.49(1.09-2.04) | 0.350 | 1.43(0.90-2.26) | <0.001 |
| Prostate cancer | | 9 | 1248 | | 1483 | 1.34(1.16-1.54) | 0.350 | 1.96(1.41-2.73) | 0.645 | 1.51(1.09-2.10) | 0.529 | 1.73(1.27-2.37) | 0.597 | 1.32(1.09-1.60) | 0.295 |
| Endometrial cancer | | 6 | 1222 | | 1922 | 1.12(0.73-1.72) | 0.019 | 1.45(0.47-4.47) | 0.803 | 1.27(0.43-3.76) | 0.891 | 1.37(0.45-4.21) | 0.831 | 1.11(0.71-1.73) | 0.025 |
| Hepatocellular cancer | | 3 | 1354 | | 1593 | 1.10(0.97-1.24) | 0.868 | 1.34(0.97-1.85) | 0.632 | 1.29(0.92-1.79) | 0.635 | 1.32(0.96-1.81) | 0.608 | 1.08(0.92-1.26) | 0.972 |
| Gastric cancer | | 4 | 598 | | 1966 | 1.01(0.74-1.37) | 0.103 | 1.64(0.58-4.65) | 0.049 | 1.73(1.01-2.97) | 0.047 | 1.79(0.62-5.12) | 0.041 | 0.88(0.69-1.12) | 0.580 |
| Cervical cancer | | 3 | 265 | | 317 | 1.50(0.72-3.12) | 0.017 | 1.59(0.08-31.34) | 0.058 | 1.17(0.40-3.42) | 0.146 | 1.39(0.08-23.27) | 0.071 | 1.67(0.86-3.27) | 0.068 |
| Oral cancer | | 6 | 813 | | 977 | 1.10(0.80-1.51) | 0.016 | 1.47(0.69-3.14) | 0.061 | 1.45(0.89-2.37) | 0.353 | 1.44(0.74-2.8) | 0.127 | 1.07(0.77-1.49) | 0.047 |
| Others | | 10 | 2670 | | 4032 | 1.11(0.93-1.33) | 0.014 | 0.96(0.73-1.26) | 0.983 | 0.84(0.64-1.11) | 0.887 | 0.91(0.70-1.19) | 0.982 | 1.19(0.94-1.50) | 0.002 |
| **Ethnicity** | |  |  | |  |  |  |  |  |  |  |  |  |  |  |
| Caucasian | | 45 | 14019 | | 16965 | 1.18(1.05-1.32) | <0.001 | 1.90(1.45-2.51) | 0.674 | 1.53(1.19-1.99) | 0.707 | 1.74(1.34-2.26) | 0.782 | 1.17(1.03-1.33) | <0.001 |
| Asian | | 67 | 15280 | | 20403 | 1.19(1.11-1.27) | <0.001 | 1.46(1.26-1.68) | <0.001 | 1.24(1.12-1.36) | 0.059 | 1.38(1.21-1.57) | 0.001 | 1.19(1.10-1.28) | <0.001 |
| African | | 3 | 105 | | 257 | 1.41(0.38-5.30) | 0.556 |  |  |  |  |  |  | 1.42(0.37-5.40) | 0.542 |
| Mixed | | 19 | 5062 | | 6746 | 1.12(0.91-1.39) | <0.001 | 1.57(0.91-2.70) | 0.009 | 1.37(1.02-1.83) | 0.128 | 1.42(0.87-2.33) | 0.032 | 1.15(0.89-1.49) | <0.001 |
| **Control source** | |  |  | |  |  |  |  |  |  |  |  |  |  |  |
| Hospital based | | 79 | 18126 | | 19987 | 1.17(1.10-1.25) | <0.001 | 1.50(1.31-1.71) | 0.113 | 1.35(1.21-1.51) | 0.485 | 1.43(1.27-1.61) | 0.246 | 1.15(1.07-1.25) | <0.001 |
| Population based | | 52 | 15023 | | 22606 | 1.21(1.09-1.34) | <0.001 | 1.63(1.25-2.12) | <0.001 | 1.18(1.02-1.36) | 0.025 | 1.48(1.17-1.88) | <0.001 | 1.22(1.08-1.38) | <0.001 |
| Mixed | | 3 | 1317 | | 1778 | 1.10(0.88-1.39) | 0.250 | 1.13(0.54-2.35) | 0.311 | 0.97(0.52-1.82) | 0.395 | 1.10(0.52-2.32) | 0.298 | 1.11(0.90-1.38) | 0.378 |
| **Sample size(both cases and controls)** | | | | |  | |  |  |  |  |  |  |  |  |  |
| <500 | 92 | | | 11192 | 14075 | 1.30(1.19-1.42) | <0.001 | 1.83(1.53-2.17) | 0.002 | 1.49(1.31-1.69) | 0.270 | 1.67(1.43-1.95) | 0.024 | 1.30(1.17-1.45) | <0.001 |
| ≥500 | 42 | | | 23274 | 30296 | 1.05(0.99-1.10) | 0.013 | 1.17(1.02-1.36) | 0.139 | 1.12(0.99-1.26) | 0.258 | 1.15(1.00-1.32) | 0.170 | 1.04(0.98-1.11) | 0.030 |
| ^a^Number of studies.  ***P***^b^ The value of heterogeneity test.  ^c^Stratified according to subjects ≥500 in both case and control groups or not. | | | | | | | | | | | | | | | |
