# Supplementary material for: MspI and Ile462Val Polymorphisms in CYP1A1 and Overall Cancer Risk: A Meta-Analysis
Source: PLoS One. 2013 Dec 31;8(12):e85166. doi: 10.1371/journal.pone.0085166 (PMC3877352; doi:10.1371/journal.pone.0085166)
Supplement: Table S3 — Characteristics of studies included in the meta-analysis for MspI polymorphism. A generalized distribution of MspI genotype frequencies for each included study is listed. (DOC) [file pone.0085166.s005.doc]

| **Table S3.Characteristics of studies included in the meta-analysis for the MspI polymorphism** | | | | | | | | |
| --- | --- | --- | --- | --- | --- | --- | --- | --- |
|  | | | | | | | | |
| First author | Year | Ethnicity | Country | Cancer types | Methods | Source of con | Case | Con |
| Wang | 2012 | Asian | China | renal carcinoma | PCR-RFLP | HB | 207 | 236 |
| Souiden | 2012 | Caucasian | Tunisia | prostate cancer | PCR-RFLP | HB | 138 | 138 |
| Matei | 2012 | Caucasian | Romania | ovarian cancer | PCR-RFLP | HB | 21 | 21 |
| Khvostova | 2012 | Caucasian | Russia | breast cancer | PCR-RFLP | HB | 276 | 274 |
| Lopez-Cima | 2012 | Caucasian | Spain | lung cancer | PCR-RFLP | HB | 789 | 789 |
| Li | 2012 | Asian | China | lung cancer | PCR-RFLP | HB | 217 | 198 |
| Kiyohara | 2012 | Asian | Japan | lung cancer | PCR | HB | 462 | 379 |
| Kim | 2012 | Asian | Korea | leukemia | real-time PCR | PB | 415 | 1700 |
| Bonaventure | 2012 | Caucasian | France | leukemia | infinim platform | PB | 430 | 548 |
| Bonaventure | 2012 | Caucasian | France | leukemia | infinim platform | PB | 50 | 548 |
| Swinney | 2011 | Caucasian | USA | leukemia | Golden Gate Assay | PB | 60 | 173 |
| Rudolph | 2011 | Caucasian | Germany | colorectal cancer | KASPar assays | PB | 679 | 679 |
| Naushad | 2011 | Asian | India | breast cancer | PCR-RFLP | PB | 342 | 253 |
| Luo | 2011 | Asian | China | gastric cancer | PCR-RFLP | PB | 123 | 129 |
| Kristiansen | 2011 | Caucasian | Norway | testicular cancer | real-time PCR | PB | 651 | 199 |
| Kiruthiga | 2011 | Asian | India | breast cancer | PCR-RFLP | PB | 50 | 50 |
| Ihsan | 2011 | Asian | India | lung cancer | PCR-RFLP | PB | 188 | 290 |
| Yamaguti | 2010 | Caucasian | Brazil | leukemia | PCR | PB | 99 | 99 |
| Wright | 2010 | Caucasian | Australia | lung cancer | PCR-RFLP | PB and HB | 1040 | 784 |
| Moreno-Galvan | 2010 | mixed | Mexico | breast cancer | PCR-RFLP | HB | 91 | 94 |
| Syamala | 2010 | Asian | India | breast cancer | PCR-RFLP | PB | 219 | 367 |
| Sharma | 2010 | Asian | India | head and neck cancer | PCR-RFLP | PB | 203 | 201 |
| Nisa | 2010 | Asian | Japan | colorectal cancer | PCR-RFLP | PB | 685 | 778 |
| Malik | 2010 | Asian | India | esophageal carcinoma | PCR | HB | 135 | 195 |
| Kumar | 2010 | Caucasian | India | prostate cancer | PCR-RFLP and allele-specific PCR | HB | 70 | 71 |
| Cleary | 2010 | Caucasian | Canada | colorectal cancer | TaqMan | PB | 1163 | 1290 |
| Ashton | 2010 | Caucasian | Australia | endometrial cancer | PCR-RFLP | PB | 191 | 287 |
| MARIE-GENICA | 2010 | Caucasian | Germany | breast cancer | MassARRAY platform | PB | 3148 | 5482 |
| Yamaguti | 2009 | mixed | Brazil | leukemia | PCR-RFLP | PB | 133 | 133 |
| Singh | 2009 | Asian | India | head and neck cancer | PCR-RFLP | PB | 200 | 200 |
| Shimada | 2009 | Mixed | Japan | breast cancer | TaqMan | HB | 873 | 873 |
| Sangrajrang | 2009 | Asian | Thailand | breast cancer | TaqMan | HB | 568 | 496 |
| Malik | 2009 | Caucasian | India | gastric cancer | PCR-RFLP | HB | 108 | 195 |
| Ociepa-Zawal | 2009 | Caucasian | Poland | breast cancer | PCR-RFLP | HB | 71 | 100 |
| Lee | 2009 | Asian | Korea | leukemia | SNaPshot | HB | 160 | 162 |
| Kim | 2009 | Asian | Korea | lymphoma | simplex Pyrosequencing assays | PB | 711 | 1700 |
| Kato | 2009 | African | USA | breast cancer | TaqMan | PB | 194 | 189 |
| Honma | 2009 | Mixed | Brazil | lung cancer | PCR-RFLP | HB | 200 | 264 |
| Gutman | 2009 | Asian | Israel | cervical cancer | PCR-RFLP | HB | 43 | 121 |
| Cote | 2009 | mixed | USA | lung cancer | PCR-RFLP and TaqMan | PB | 496 | 519 |
| Yuan | 2008 | Asian | China | hepatocellular carcinoma | PCR-RFLP | HB | 296 | 185 |
| Srivastava | 2008 | Asian | India | bladder cancer | PCR-RFLP | HB | 106 | 160 |
| Siraj | 2008 | Asian | Saudi Arabia | thyroid cancer | Hotstart Taq polymerase | PB | 47 | 509 |
| Sam | 2008 | Asian | India | head and neck cancer | PCR-RFLP | HB | 408 | 220 |
| Pandey | 2008 | Asian | India | gallbladder cancer | PCR-RFLP | PB and HB | 142 | 171 |
| Nishino | 2008 | Asian | Japan | cervical cancer | TaqMan | HB | 124 | 117 |
| Majumdar | 2008 | Asian | India | leukemia | PCR-RFLP | PB | 110 | 126 |
| Lima | 2008 | Mixed | Brazil | prostate cancer | sequencing | HB | 125 | 100 |
| Justenhoven | 2008 | Caucasian | Germany | breast cancer | MALDI-TOF MS | PB | 600 | 618 |
| Hirata | 2008 | Caucasian | Japan | endometrial cancer | PCR-RFLP | PB | 150 | 165 |
| Harth | 2008 | Caucasian | Germany | head and neck cancer | PCR-RFLP | HB | 312 | 300 |
| Gulyaeva | 2008 | Caucasian | Russia | breast cancer | PCR-RFLP | PB | 118 | 188 |
| Gulyaeva | 2008 | Caucasian | Russia | ovarian cancer | PCR-RFLP | PB | 96 | 188 |
| Gulyaeva | 2008 | Caucasian | Russia | endometrial cancer | PCR-RFLP | PB | 154 | 188 |
| Gallegos-Arreola | 2008 | Mixed | Mexico | leukemia | PCR | PB | 210 | 228 |
| Figueroa | 2008 | Mixed | USA | testicular cancer | TaqMan | HB | 508 | 603 |
| Al-Dayel | 2008 | Asian | Saudi Arabia | lymphoma | PCR-RFLP | PB | 152 | 509 |
| Yoshida | 2007 | Asian | Japan | colorectal cancer | PCR-RFLP | HB | 66 | 121 |
| Singh | 2007 | Asian | India | breast cancer | PCR-RFLP | PB | 146 | 162 |
| Jain | 2007 | Asian | India | esophageal carcinoma | PCR-RFLP | PB | 161 | 201 |
| Singh | 2007 | Asian | India | breast cancer | PCR-RFLP | HB | 105 | 116 |
| Mittal | 2007 | Caucasian | India | prostate cancer | PCR-RFLP | HB | 130 | 140 |
| McGrath | 2007 | mixed | USA | endometrial cancer | TaqMan | HB | 406 | 1008 |
| Juarez-Cedillo | 2007 | mixed | Mexico | cervical cancer | PCR-RFLP | HB | 155 | 155 |
| Holt | 2007 | mixed | USA | ovarian cancer | TaqMan | PB | 310 | 573 |
| Cote. | 2007 | mixed | USA | lung cancer | PCR-RFLP | PB | 354 | 440 |
| Bolufer | 2007 | Caucasian | Spain | leukemia | real-time PCR | PB | 291 | 403 |
| Yang | 2006 | Asian | China | prostate cancer | PCR-RFLP and allele-specific PCR | HB | 225 | 250 |
| Shen | 2006 | Asian | China | breast cancer | PCR-RFLP | PB | 250 | 268 |
| Quinones | 2006 | Caucasian | Chile | prostate cancer | PCR-RFLP | HB | 60 | 117 |
| Pisani | 2006 | Asian | Thailand | lung cancer | PCR | PB and HB | 168 | 286 |
| Ma | 2006 | Asian | China | gastric cancer | PCR-RFLP | HB | 60 | 57 |
| Little | 2006 | Caucasian | UK | colorectal cancer | PCR | PB | 232 | 378 |
| Lira | 2006 | Caucasian | Italy | skin cancer | PCR | HB | 106 | 132 |
| Joseph | 2006 | Asian | India | cervical cancer | PCR-RFLP | HB | 147 | 165 |
| Aydin-Sayitoglu | 2006 | Caucasian | Turkey | leukemia | PCR-RFLP | PB | 155 | 140 |
| Agudo | 2006 | Caucasian | Spain | gastric cancer | PCR | PB | 243 | 940 |
| Wenzlaff | 2005 | mixed | USA | lung cancer | PCR-RFLP | PB | 128 | 150 |
| Vijayalakshmi | 2005 | Caucasian | India | prostate cancer | PCR-RFLP | HB | 50 | 50 |
| Sreeja | 2005 | Asian | India | lung cancer | PCR-RFLP | HB | 146 | 146 |
| Okobia | 2005 | African | Nigeria | breast cancer | PCR | HB | 220 | 218 |
| Ng | 2005 | Asian | Singapore | lung cancer | allele-specific PCR | HB | 124 | 162 |
| Li | 2005 | Caucasian | USA | breast cancer | PCR-RFLP | PB | 413 | 415 |
| Li | 2005 | African | USA | breast cancer | PCR-RFLP | PB | 265 | 280 |
| Demir | 2005 | Caucasian | Turkey | lung cancer | PCR-RFLP | HB | 31 | 37 |
| Le Marchand | 2005 | Mixed | USA | breast cancer | PCR-RFLP | PB | 1339 | 1370 |
| Doherty | 2005 | Mixed | USA | endometrial cancer | PCR-RFLP | PB | 371 | 420 |
| Clavel | 2005 | Caucasian | France | leukemia | PCR-RFLP | HB | 217 | 105 |
| Caceres | 2005 | Mixed | Chile | prostate cancer | PCR-RFLP | PB | 102 | 130 |
| Boyapati | 2005 | Asian | USA | breast cancer | PCR-RFLP | PB | 1120 | 1196 |
| Yin | 2004 | Asian | Taiwan | hepatocellular carcinoma | PCR-RFLP | PB | 54 | 86 |
| Slattery | 2004 | Mixed | USA | colorectal cancer | PCR | HB | 1797 | 2164 |
| Joseph | 2004 | Asian | India | leukemia | PCR-RFLP | HB | 118 | 118 |
| Hefler | 2004 | Caucasian | Austria | breast cancer | PCR | PB | 404 | 1699 |
| Canalle | 2004 | Mixed | Brazil | leukemia | PCR-RFLP | HB | 113 | 221 |
| Wang | 2003 | Asian | China | lung cancer | PCR-RFLP | HB | 162 | 181 |
| Varzim | 2003 | Caucasian | Portugal | head and neck cancer | PCR-RFLP | HB | 88 | 178 |
| Terry | 2003 | Mixed | USA | ovarian cancer | PCR-RFLP | PB | 438 | 465 |
| Taioli | 2003 | Mixed | France | lung cancer | PCR | PB and HB | 109 | 424 |
| Sugawara | 2003 | Asian | Japan | cervical cancer | PCR | HB | 72 | 31 |
| Sugawara | 2003 | Asian | Japan | ovarian cancer | PCR | HB | 46 | 31 |
| Sugawara | 2003 | Asian | Japan | endometrial cancer | PCR | HB | 38 | 31 |
| Sobti | 2003 | Asian | India | lung cancer | PCR-RFLP and allele-specific PCR | PB | 100 | 76 |
| Kiyohara | 2003 | Asian | Japan | lung cancer | PCR-RFLP | HB | 158 | 259 |
| Gronau | 2003 | Caucasian | Germany | head and neck cancer | PCR-RFLP | HB | 187 | 139 |
| Cheng | 2003 | Asian | Taiwan | head and neck cancer | PCR-RFLP | PB | 172 | 218 |
| Chang | 2003 | Mixed | USA | prostate cancer | sequencing | HB | 224 | 180 |
| Acevedo | 2003 | Mixed | Chile | prostate cancer | PCR-RFLP | PB | 102 | 128 |
| Ye | 2002 | Caucasian | UK | colorectal cancer | PCR-RFLP | PB | 41 | 82 |
| Wu | 2002 | Asian | Taiwan | esophageal carcinoma | PCR-RFLP | HB | 146 | 324 |
| Miyoshi | 2002 | Asian | Japan | breast cancer | PCR | PB | 195 | 272 |
| Li | 2002 | Caucasian | USA | pancreatic cancer | PCR-RFLP | HB | 30 | 34 |
| Yin | 2001 | Asian | China | lung cancer | PCR | PB | 84 | 84 |
| Song | 2001 | Asian | China | lung cancer | PCR-RFLP | PB | 217 | 404 |
| Sarmanova | 2001 | Caucasian | Norway | lymphoma | PCR-RFLP | HB | 226 | 419 |
| Krajinovic | 2001 | Caucasian | Canada | breast cancer | allele-specific PCR | HB | 135 | 201 |
| Ko | 2001 | Caucasian | Germany | head and neck cancer | PCR-RFLP | PB | 312 | 300 |
| Inoue | 2000 | Asian | Japan | colorectal cancer | PCR-RFLP | HB | 95 | 90 |
| Yu | 1999 | Asian | Taiwan | hepatocellular carcinoma | PCR-RFLP | HB | 81 | 409 |
| van Lieshout | 1999 | Caucasian | Netherland | esophageal carcinoma | PCR-RFLP | HB | 34 | 247 |
| Taioli | 1999 | African | USA | breast cancer | PCR | PB | 25 | 118 |
| Tanimoto | 1999 | Asian | Japan | oral cancer | PCR-RFLP | HB | 100 | 100 |
| Sato | 1999 | Asian | Japan | oral cancer | PCR | HB | 142 | 142 |
| Persson | 1999 | Asian | Sweden | lung cancer | PCR | PB | 80 | 76 |
| Krajinovic | 1999 | Caucasian | Canada | leukemia | PCR-RFLP | PB | 170 | 299 |
| Huang | 1999 | Asian | Taiwan | breast cancer | PCR-RFLP | HB | 141 | 145 |
| Taioli | 1998 | Mixed | USA | lung cancer | PCR-RFLP and allele-specific PCR | HB | 98 | 289 |
| Hong | 1998 | Asian | Korea | lung cancer | PCR | HB | 85 | 63 |
| Matthias | 1998 | Caucasian | Germany | head and neck cancer | PCR | HB | 122 | 205 |
| Matthias | 1998 | Caucasian | Germany | head and neck cancer | PCR | HB | 262 | 205 |
| Fontana | 1998 | Caucasian | France | breast cancer | PCR | PB | 486 | 100 |
| Fontana | 1998 | Caucasian | France | cervical cancer | PCR | PB | 118 | 91 |
| Bailey | 1998 | African | USA | breast cancer | PCR-RFLP | HB | 59 | 59 |
| Garcia-Closas | 1997 | Mixed | USA | lung cancer | PCR | HB | 416 | 446 |
| Esteller | 1997 | Caucasian | Spain | endometrial cancer | PCR-RFLP | HB | 80 | 60 |
| Yengi | 1996 | Caucasian | Scotland | skin cancer | PCR | HB | 257 | 82 |
| Xu | 1996 | Mixed | USA | lung cancer | PCR | HB | 207 | 283 |
| Taioli | 1995 | Caucasian | USA | breast cancer | PCR | PB | 30 | 183 |
| Taioli | 1995 | African | USA | breast cancer | PCR | PB | 29 | 175 |
| Kihara | 1995 | Asian | Japan | lung cancer | PCR | HB | 97 | 258 |
| Sivaraman | 1994 | Mixed | USA | colorectal cancer | PCR-RFLP | PB | 43 | 47 |
| Kelsey | 1994 | African | USA | lung cancer | PCR-RFLP | PB | 72 | 97 |
| Alexandrie | 1994 | Caucasian | Sweden | lung cancer | PCR | PB | 296 | 329 |
| Drakoulis | 1994 | Caucasian | Germany | lung cancer | PCR-RFLP and allele-specific PCR | HB | 142 | 171 |
| Shields | 1993 | Mixed | USA | lung cancer | PCR | HB | 56 | 48 |
| Nakachi | 1993 | Asian | Japan | lung cancer | allele-specific PCR | PB | 31 | 127 |
| Hirvonen | 1993 | Caucasian | Finland | lung cancer | PCR-RFLP | HB | 87 | 121 |
| Tefre | 1991 | Caucasian | Norway | lung cancer | PCR-RFLP | PB | 221 | 212 |
