# Supplementary material for: MspI and Ile462Val Polymorphisms in CYP1A1 and Overall Cancer Risk: A Meta-Analysis
Source: PLoS One. 2013 Dec 31;8(12):e85166. doi: 10.1371/journal.pone.0085166 (PMC3877352; doi:10.1371/journal.pone.0085166)
Supplement: Table S4 — Characteristics of studies included in the meta-analysis for Ile462Val polymorphism. A generalized distribution of Ile462Val genotype frequencies for each included studies is listed. (DOC) [file pone.0085166.s006.doc]

| **Table S4.Characteristics of studies included in the meta-analysis for the Ile462Val polymorphism** | | | | | | | | |
| --- | --- | --- | --- | --- | --- | --- | --- | --- |
|  | | | | | | | | |
| First author | Year | Ethnicity | Country | Cancer types | Methods | Source of con | Case | Con |
| Wang | 2012 | Asian | China | renal carcinoma | PCR-RFLP | HB | 207 | 236 |
| Wang | 2012 | Asian | China | esophageal carcinoma | microarray | PB | 253 | 254 |
| Wang | 2012 | Asian | China | esophageal carcinoma | microarray | PB | 312 | 214 |
| Matei | 2012 | Caucasian | Romania | ovarian cancer | PCR-RFLP | HB | 21 | 21 |
| Kiyohara | 2012 | Asian | Japan | lung cancer | PCR | HB | 462 | 379 |
| Kim | 2012 | Asian | Korea | leukemia | real-time PCR | PB | 406 | 1696 |
| Wang | 2011 | Asian | China | breast cancer | sequencing | HB | 400 | 400 |
| Swinney | 2011 | Caucasian | USA | leukemia | Golden Gate Assay | PB | 71 | 204 |
| Naushad | 2011 | Asian | India | breast cancer | PCR-RFLP | PB | 342 | 253 |
| Ozturk | 2011 | Caucasian | Turkey | bladder cancer | PCR-RFLP | HB | 176 | 98 |
| Kohno | 2011 | Asian | Japan | lung cancer | PCR | HB | 377 | 325 |
| Kiruthiga | 2011 | Asian | India | breast cancer | PCR-RFLP | PB | 50 | 50 |
| Ihsan | 2011 | Asian | India | lung cancer | PCR-RFLP | PB | 188 | 290 |
| Yamaguti | 2010 | Caucasian | Brazil | leukemia | PCR | PB | 99 | 99 |
| Wright | 2010 | Caucasian | Australia | lung cancer | PCR-RFLP | PB and HB | 1040 | 784 |
| Heubner | 2010 | Caucasian | Germany | ovarian cancer | PCR-RFLP | HB | 111 | 119 |
| Tai | 2010 | Asian | China | head and neck cancer | PCR-RFLP | PB | 278 | 278 |
| Moreno-Galvan | 2010 | Mixed | Mexico | breast cancer | PCR-RFLP | HB | 91 | 94 |
| Nisa | 2010 | Asian | Japan | colorectal cancer | PCR-RFLP | PB | 685 | 778 |
| Kumar | 2010 | Caucasian | India | prostate cancer | PCR-RFLP and allele-specific PCR | HB | 70 | 61 |
| Ferlin | 2010 | Caucasian | Italy | testicular cancer | PCR | HB | 234 | 218 |
| Ashton | 2010 | Caucasian | Australia | endometrial cancer | PCR-RFLP | PB | 191 | 271 |
| MARIE-GENICA | 2010 | Caucasian | Germany | breast cancer | MassARRAY platform | PB | 3146 | 5484 |
| Yamaguti | 2009 | Mixed | Brazil | leukemia | PCR-RFLP | PB | 133 | 133 |
| Singh | 2009 | Asian | India | head and neck cancer | PCR-RFLP | PB | 200 | 200 |
| Shimada | 2009 | Mixed | Japan | breast cancer | TaqMan | HB | 866 | 873 |
| Sangrajrang | 2009 | Asian | Thailand | breast cancer | TaqMan | HB | 544 | 480 |
| Park | 2009 | Asian | USA | biliary tract cancers | TaqMan | PB | 406 | 773 |
| Li | 2009 | Asian | China | hepatocellular carcinoma | TaqMan | HB | 970 | 1000 |
| Ociepa-Zawal | 2009 | Caucasian | Poland | breast cancer | PCR-RFLP | HB | 70 | 100 |
| Lee | 2009 | Asian | Korea | leukemia | SNaPshot | HB | 160 | 159 |
| Kumar | 2009 | Asian | India | lung cancer | PCR-RFLP | PB | 93 | 253 |
| Kobayashi | 2009 | Asian | Japan | colorectal cancer | MassARRAY | HB | 105 | 225 |
| Kobayashi | 2009 | Asian | Japan | gastric cancer | MassARRAY | HB | 141 | 286 |
| Kim | 2009 | Asian | Korea | lymphoma | simplex Pyrosequencing assays | PB | 713 | 1700 |
| Gutman | 2009 | Asian | Israel | cervical cancer | PCR-RFLP | HB | 43 | 121 |
| Cote | 2009 | Mixed | USA | lung cancer | PCR-RFLP and TaqMan | PB | 502 | 523 |
| Yuan | 2008 | Asian | China | hepatocellular carcinoma | PCR-RFLP | HB | 303 | 184 |
| Yoon | 2008 | Asian | Korea | lung cancer | TaqMan | HB | 213 | 213 |
| Taspinar | 2008 | Caucasian | Turkey | leukemia | PCR-RFLP | PB | 107 | 135 |
| Sam | 2008 | Asian | India | head and neck cancer | PCR-RFLP | HB | 408 | 220 |
| Pereira Serafim | 2008 | Mixed | Brazil | colorectal cancer | PCR-RFLP | PB | 114 | 114 |
| Gallegos-Arreola | 2008 | Mixed | Mexico | lung cancer | PCR-RFLP | PB | 222 | 248 |
| Li | 2008 | Asian | China | prostate cancer | sequencing | HB | 208 | 230 |
| Kimura | 2008 | Caucasian | Japan | gallbladder cancer | PCR-RFLP | HB | 37 | 48 |
| Justenhoven | 2008 | Caucasian | Germany | breast cancer | MALDI-TOF MS | PB | 604 | 619 |
| Hirata | 2008 | Caucasian | Japan | endometrial cancer | PCR-RFLP | PB | 150 | 165 |
| Harth | 2008 | Caucasian | Germany | head and neck cancer | PCR-RFLP | HB | 312 | 300 |
| Figueroa | 2008 | Mixed | USA | testicular cancer | TaqMan | HB | 502 | 596 |
| Yoshida | 2007 | Asian | Japan | colorectal cancer | PCR-RFLP | HB | 66 | 121 |
| Singh | 2007 | Asian | India | breast cancer | PCR-RFLP | PB | 145 | 162 |
| Yeh | 2007 | Asian | Taiwan | colorectal cancer | PCR-RFLP | HB | 717 | 729 |
| Yang | 2007 | Asian | Korea | lung cancer | TaqMan | HB | 314 | 349 |
| Tsuchiya | 2007 | Asian | Japan | gallbladder cancer | PCR-RFLP | HB | 54 | 178 |
| Singh | 2007 | Asian | India | breast cancer | PCR-RFLP | HB | 105 | 116 |
| Sillanpaa | 2007 | Caucasian | Finland | breast cancer | PCR-RFLP | PB | 481 | 479 |
| Shin | 2007 | Asian | Korea | breast cancer | SnapShot assays | HB | 493 | 437 |
| McGrath | 2007 | Mixed | USA | endometrial cancer | TaqMan | HB | 392 | 975 |
| Yang | 2006 | Asian | China | prostate cancer | PCR-RFLP and allele-specific PCR | HB | 225 | 250 |
| Sugimura | 2006 | Asian | Japan | oral cancer | PCR-RFLP | HB | 122 | 241 |
| Pisani | 2006 | Asian | Thailand | lung cancer | PCR | HB and PB | 167 | 287 |
| Marques | 2006 | Mixed | Brazil | oral cancer | PCR-RFLP | HB | 231 | 212 |
| Little | 2006 | Caucasian | UK | colorectal cancer | PCR | PB | 251 | 396 |
| Joseph | 2006 | Asian | India | cervical cancer | PCR-RFLP | HB | 147 | 165 |
| Agudo | 2006 | Caucasian | Spain | gastric cancer | PCR | PB | 243 | 936 |
| Shen | 2005 | Asian | China | gastric cancer | PCR-RFLP | HB | 112 | 682 |
| Ng | 2005 | Asian | Singapore | lung cancer | allele-specific PCR | HB | 126 | 161 |
| Li | 2005 | Asian | China | gastric cancer | PCR | HB | 102 | 62 |
| Landi | 2005 | Caucasian | Spain | lung cancer | PCR | HB | 362 | 323 |
| Hou | 2005 | Caucasian | USA | colorectal cancer | TaqMan | HB | 675 | 679 |
| Doherty | 2005 | Mixed | USA | endometrial cancer | PCR-RFLP | PB | 371 | 420 |
| Chacko | 2005 | Asian | India | breast cancer | PCR-RFLP | HB | 112 | 112 |
| Boyapati | 2005 | Asian | USA | breast cancer | PCR-RFLP | PB | 1131 | 1209 |
| Yang | 2004 | Asian | USA | lung cancer | TaqMan | PB | 197 | 144 |
| Wang | 2004 | Asian | China | esophageal carcinoma | PCR | HB | 127 | 101 |
| Joseph | 2004 | Asian | India | leukemia | PCR-RFLP | HB | 118 | 118 |
| Gallegos-Arreola | 2004 | Caucasian | Mexico | leukemia | PCR | PB | 136 | 136 |
| Xie | 2004 | Mixed | USA | oral cancer | PCR | PB | 132 | 143 |
| Aktas | 2004 | Caucasian | Turkey | prostate cancer | allele-specific PCR | HB | 100 | 107 |
| Wang | 2003 | Asian | China | esophageal carcinoma | PCR | PB | 62 | 38 |
| Ozturk | 2003 | Caucasian | Turkey | lung cancer | PCR | HB | 55 | 65 |
| Terry | 2003 | Mixed | USA | ovarian cancer | PCR-RFLP | PB | 440 | 471 |
| Taioli | 2003 | Mixed | France | lung cancer | PCR | PB and HB | 110 | 707 |
| Suzuki | 2003 | Asian | Japan | prostate cancer | PCR-RFLP and allele-specific PCR | HB | 81 | 105 |
| Sugawara | 2003 | Asian | Japan | cervical cancer | PCR | HB | 75 | 31 |
| Sugawara | 2003 | Asian | Japan | ovarian cancer | PCR | HB | 45 | 31 |
| Sugawara | 2003 | Asian | Japan | endometrial cancer | PCR | HB | 38 | 31 |
| Gronau | 2003 | Caucasian | Germany | head and neck cancer | PCR-RFLP | HB | 187 | 139 |
| Chang | 2003 | Mixed | USA | prostate cancer | sequencing | HB | 224 | 180 |
| Wu | 2002 | Asian | Taiwan | esophageal carcinoma | PCR-RFLP | HB | 146 | 324 |
| Wang | 2002 | Asian | China | esophageal carcinoma | PCR | HB | 127 | 101 |
| Sunaga | 2002 | Asian | Japan | lung cancer | PCR-RFLP | HB | 198 | 152 |
| Miyoshi | 2002 | Asian | Japan | breast cancer | PCR | PB | 195 | 272 |
| Hahn | 2002 | Caucasian | Germany | oral cancer | PCR | PB | 94 | 92 |
| Beer | 2002 | Mixed | USA | prostate cancer | PCR-RFLP | PB | 110 | 146 |
| Ratnasinghe | 2001 | Caucasian | USA | lung cancer | PCR | HB | 282 | 324 |
| Quinones | 2001 | Caucasian | Chile | lung cancer | PCR-RFLP | PB | 60 | 120 |
| Murata | 2001 | Caucasian | Japan | prostate cancer | PCR-RFLP | HB | 115 | 200 |
| Ko | 2001 | Caucasian | Germany | head and neck cancer | PCR-RFLP | PB | 312 | 300 |
| Chen | 2001 | Asian | China | lung cancer | allele-specific PCR | PB | 106 | 106 |
| Basham | 2001 | Caucasian | UK | breast cancer | PCR | HB | 1948 | 1355 |
| Sato | 2000 | Asian | Japan | oral cancer | allele-specfic PCR | HB | 142 | 142 |
| Olshan | 2000 | Caucasian | USA | head and neck cancer | PCR | HB | 108 | 165 |
| McWilliams | 2000 | Mixed | USA | head and neck cancer | PCR-RFLP | PB | 139 | 121 |
| Dolzan | 2000 | Caucasian | Slovenia | lung cancer | PCR | HB | 195 | 100 |
| Yu | 1999 | Asian | Taiwan | hepatocellular carcinoma | PCR-RFLP | HB | 81 | 409 |
| van Lieshout | 1999 | Caucasian | Netherland | esophageal carcinoma | PCR-RFLP | HB | 34 | 247 |
| Taioli | 1999 | Caucasian | USA | breast cancer | PCR | PB | 29 | 185 |
| Taioli | 1999 | African | USA | breast cancer | PCR | PB | 26 | 115 |
| Morita | 1999 | Asian | Japan | head and neck cancer | PCR | HB | 145 | 164 |
| Katoh | 1999 | Asian | Japan | oral cancer | PCR | HB | 92 | 147 |
| Huang | 1999 | Asian | Taiwan | breast cancer | PCR-RFLP | HB | 143 | 145 |
| Taioli | 1998 | Mixed | USA | lung cancer | PCR-RFLP and allele-specific PCR | HB | 103 | 290 |
| Murata | 1998 | Asian | Japan | prostate cancer | allele-specific PCR | HB | 115 | 204 |
| Le Marchand | 1998 | Mixed | USA | lung cancer | PCR | PB | 337 | 453 |
| Matthias | 1998 | Caucasian | Germany | head and neck cancer | PCR | HB | 124 | 193 |
| Matthias | 1998 | Caucasian | Germany | head and neck cancer | PCR | HB | 264 | 193 |
| Matthias | 1998 | Caucasian | Germany | head and neck cancer | PCR | HB | 388 | 386 |
| Bailey | 1998 | Caucasian | USA | breast cancer | PCR-RFLP | HB | 164 | 162 |
| Bailey | 1998 | African | USA | breast cancer | PCR-RFLP | HB | 59 | 59 |
| Nimura | 1997 | Asian | Japan | esophageal carcinoma | PCR | HB | 89 | 137 |
| Morita | 1997 | Asian | Japan | esophageal carcinoma | PCR | HB | 53 | 132 |
| Hori | 1997 | Asian | Japan | esophageal carcinoma | PCR-RFLP | PB | 91 | 428 |
| Esteller | 1997 | Caucasian | Spain | endometrial cancer | PCR-RFLP | HB | 80 | 60 |
| Yengi | 1996 | Caucasian | Scotland | skin cancer | PCR | HB | 258 | 84 |
| Taioli | 1995 | Caucasian | USA | breast cancer | PCR | PB | 21 | 85 |
| Taioli | 1995 | African | USA | breast cancer | PCR | PB | 20 | 83 |
| Kihara | 1995 | Asian | Japan | lung cancer | PCR | HB | 95 | 255 |
| Katoh | 1995 | Asian | Japan | renal carcinoma | PCR-RFLP | PB | 83 | 101 |
| Ambrosone | 1995 | Caucasian | USA | breast cancer | PCR-RFLP | PB | 176 | 228 |
| Sivaraman | 1994 | Mixed | USA | colorectal cancer | PCR-RFLP | PB | 43 | 47 |
| Alexandrie | 1994 | Caucasian | Sweden | lung cancer | PCR | PB | 296 | 329 |
| Drakoulis | 1994 | Caucasian | Germany | lung cancer | PCR-RFLP and allele-specific PCR | HB | 142 | 171 |
| Nakachi | 1993 | Asian | Japan | lung cancer | allele-specific PCR | PB | 31 | 127 |
